# *Cortinarius mapuveronicae* from South America, a chemical and morphological link between European and Australian dermocyboid Cortinarii

**DOI:** 10.1007/s13659-025-00552-5

**Published:** 2026-02-02

**Authors:** Josefine Lange, Lesley Huymann, Sophie Schwarzkopf, Dilara Balci, Mehdi D. Davari, Arijana Turanovic, Clemens Gotsis, Götz Palfner, Bianka Siewert, Ursula Peintner, Norbert Arnold

**Affiliations:** 1 Department of Bioorganic Chemistry, Leibniz Institute of Plant Biochemistry, Weinberg 3, 06120 Halle (Saale), Germany; 2 Department of Microbiology, University of Innsbruck, Technikerstr. 25, 6020 Innsbruck, Austria; 3 Institute of Pharmacy, University of Innsbruck, Innrain 80-82, 6020 Innsbruck, Austria; 4 Departamento de Botanica, Facultad de Ciencias Naturales y Oceanograficas, Universidad de Concepción, Casilla, 160-C Concepción, Chile; 5 Present Address: Institute of Pharmacy, University of Hamburg, Bundesstr. 45, 20146 Hamburg, Germany

**Keywords:** *Cortinarius*, South America, Morphology, Phylogeny, Secondary metabolites, Anthraquinones, Photoantimicrobial activity

## Abstract

The new species *Cortinarius mapuveronicae* from Andean-Patagonian *Nothofagus*-forests is described in a polythetic approach combining chemical analysis of the anthraquinonoid secondary metabolites, microscopical and morphological characteristics, as well as molecular phylogeny. *C. mapuveronicae* exhibits an intense red color reaction of the basidiomata by treatment with KOH, whereas the basidiospores are turning purplish brown. As responsible compound, the new anthraquinonoid pigment clavorubin-8-*O*-methylether (**1**), together with the known monomeric and dimeric anthraquinones (+)-7,7-emodinphyscion (**2**), emodin (**3**), emodin-6,8-di-*O*-methylether (**4**), questin (**5**), (+)-(*S*)-skyrin (**6**), (+)-(*S*)-aurantioskyrin (**7**), hypericin (**8**), dermolutein (**9**), and endocrocin (**10**) could be identified, showing also remarkable activity in a (photo)antimicrobial and (photo)cytotoxic assay. Phylogenetic analysis (ITS, LSU, rpb1) demonstrates a sister group relationship with the holotype of *C. rubrobasalis*.

**Graphical Abstract:**

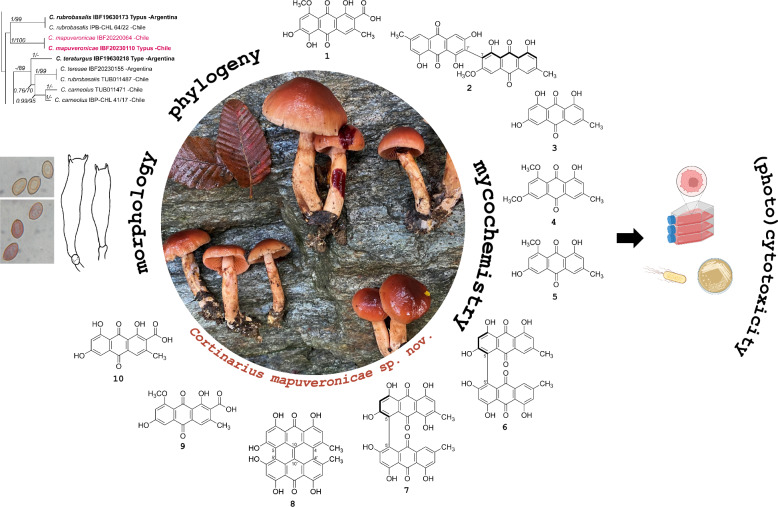

**Supplementary Information:**

The online version contains supplementary material available at 10.1007/s13659-025-00552-5.

## Introduction

South American *Nothofagaceae* forests are hot spots for the ectomycorrhizal fungal genus *Cortinarius*. More than 260 *Cortinarius* species have been described so far from Southern Chile and Argentina [1–8]. The systematics of *Cortinarius* have led to inconsistent definitions in the past. Especially the delimitation of the taxon *Dermocybe* within the Cortinarii has been debated for now 200 years and underwent many changes. *Dermocybe* was first described in 1821 by E. M. Fries [9] as a subgenus of *Cortinarius*. Influenced by different weighing of morphological/microscopical properties and pigment composition, *Dermocybe* was in a constant state of discrepancy as to whether this group should be considered as a separate genus or a subgenus of *Cortinarius* [10–15]. Modern approaches towards a more natural taxonomic characterization of species complexes in *Cortinarius* can be achieved through DNA-based phylogeny [6, 16–23] or the analysis of genomic data, which recognizes *Dermocybe* as a subgenus in *Cortinarius* [24]. Very recently, a molecular revision of central European dermocyboid *Cortinarius* species combined DNA- and pigment-based information [25]. For South American Cortinarii, this phylogenetic DNA-based approach has so far only been applied for a few species [2, 3, 5, 6, 8, 22], still leaving a broad field of research. In addition, secondary metabolites of the South American dermocyboid Cortinarii are still widely unexplored. In the past, comparative thin layer chromatography analysis revealed the presence of anthraquinones as a characteristic feature of South American dermocyboid taxa [26–28]. More recently, monomeric and dimeric (pre-) anthraquinones were isolated from *Dermocybe nahuelbutensis* [29] and their structures were elucidated by mass spectrometric and NMR spectroscopic methods.

During our ongoing studies of Chilean dermocyboid Cortinarii species in *Nothofagus* forests, we collected an unknown species phenotypically very similar to the New Zealand species *Cortinarius veronicae* Soop [30]. The present study describes this new species as *Cortinarius mapuveronicae* based on morphological and microscopical attributes, molecular phylogeny, and chemical analysis of secondary metabolites. Finally, the isolated compounds were tested for selected biological photoactivities. We are therefore able to present an integrative taxonomical approach to describe this interesting fungus from the Andean-Patagonian region of South America.

## Results

In the following we will present a polythetic taxonomical approach of a description of the new species. The classical phylogenetic and taxonomic description is complemented by a chemical approach and a test for bioactivity.

### Phylogenetic placement of *Cortinarius mapuveronicae*

Both phylogenetic analyses confirmed *Cortinarius mapuveronicae* as a distinct lineage within the genus *Cortinarius*. The alignment of the larger concatenated dataset (ITS, LSU, rpb1) contains 134 sequences and 2282 positions (ITS bp 1–815, LSU 816–1765, the rpb1 1766–2282). Here, *C. mapuveronicae* falls in a supported (1/77) clade with *C. rubrobasalis*. Tree topologies indicate a relationship of this lineage to the sections *Dermocybe* [29] and *Pauperae* (see Supplementary Information: Additional file 1, Fig. S50).

**Fig. 1:**
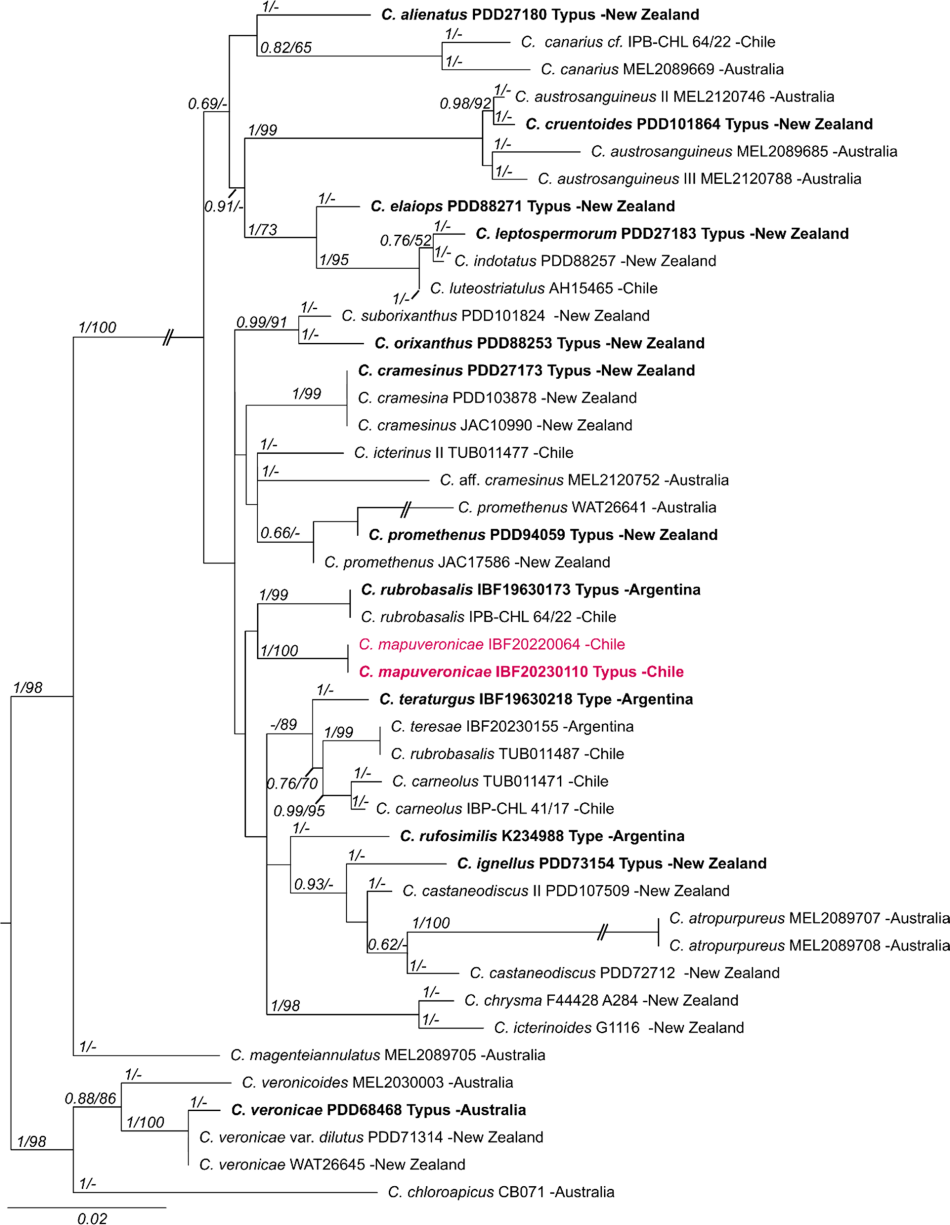
Maximum likelihood (RAxML) phylogenetic tree of selected *Cortinarius* taxa showing the distinct position of *Cortinarius mapuveronicae* compared to morphologically similar taxa. Shown is the best ML tree with bootstrap values (BS > 60) and Bayesian posterior probabilities (BPP > 0.60) provided above the branches. The dataset contains 44 samples and consists of concatenated nrITS and nrLSU sequences. Voucher numbers and geographical provenance are included in labels. Sequences originating from holotype vouchers are printed in bold

The second, smaller alignment included closely related taxa of *C. mapuveronicae* only (44 samples), and was based on rDNA sequences (ITS, LSU) and 1225 positions (ITS 1–658, LSU 659–1225) (Fig. 1). This phylogenetic analysis substantiates *C. mapuveronicae* as a distinct species with sister group relationship to *C. rubrobasalis,* although the placement is supported only by tree topologies. More importantly, it clearly shows that *C. mapuveronicae* is distinct from similar taxa with red basidiomata like *C. teresae* and *C. veronicae*. *C. rubrobasalis* can easily be misidentified as shown by a *C. teresae,* which was wrongly named *C. rubrobasalis* (Fig. 1: IBF20230155 *vs* TUB011471)*.*

### Taxonomy

**Fig. 2:**
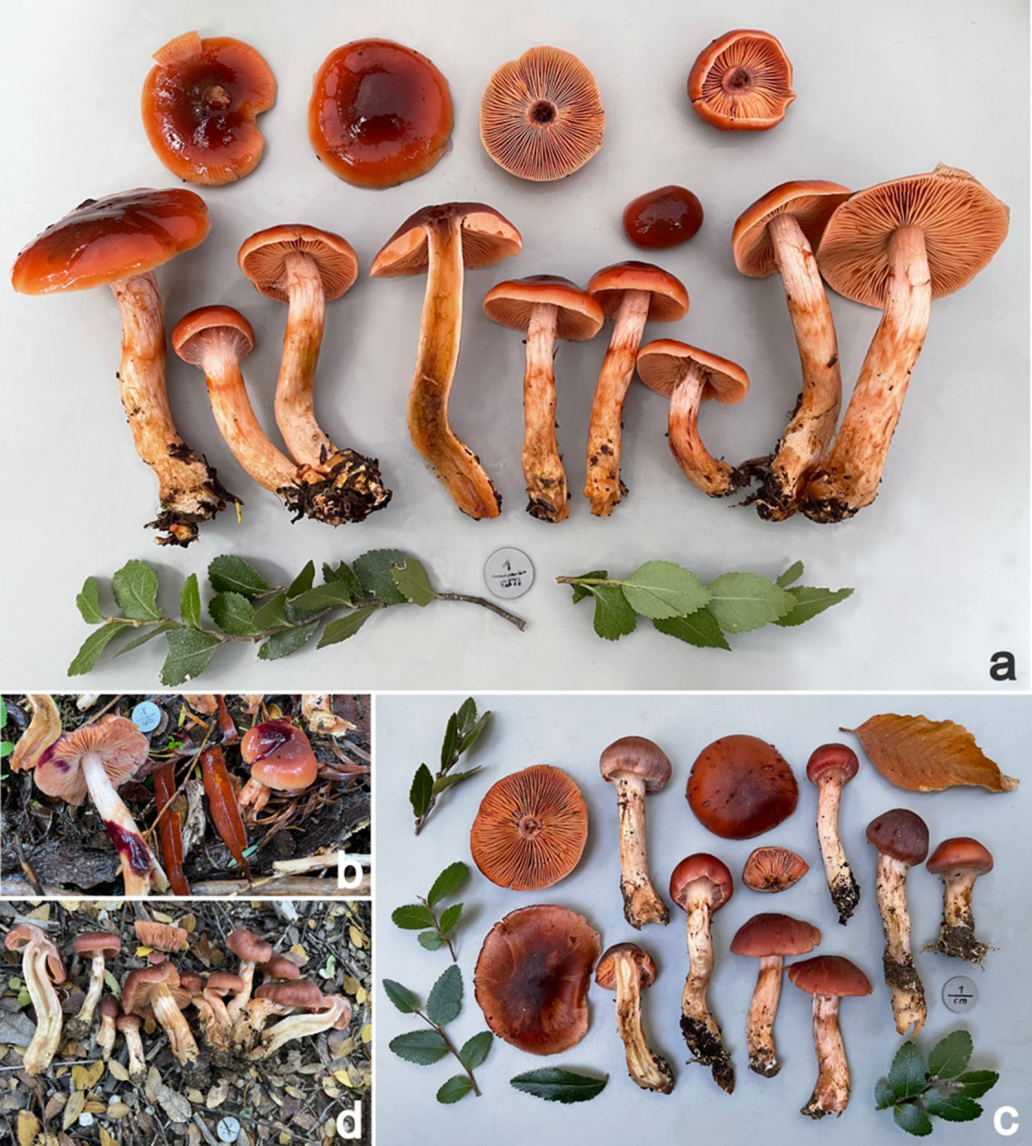
Basidiomata of *Cortinarius mapuveronicae*, **a**: fresh basidiomata of the holotype IBF20230110; **b**: positive alkaline reaction on pileus and stipe of the holotype collection IBF20230110; **c**: collection IBF20220064; **d**: collection IBF20220073

**Fig. 3:**
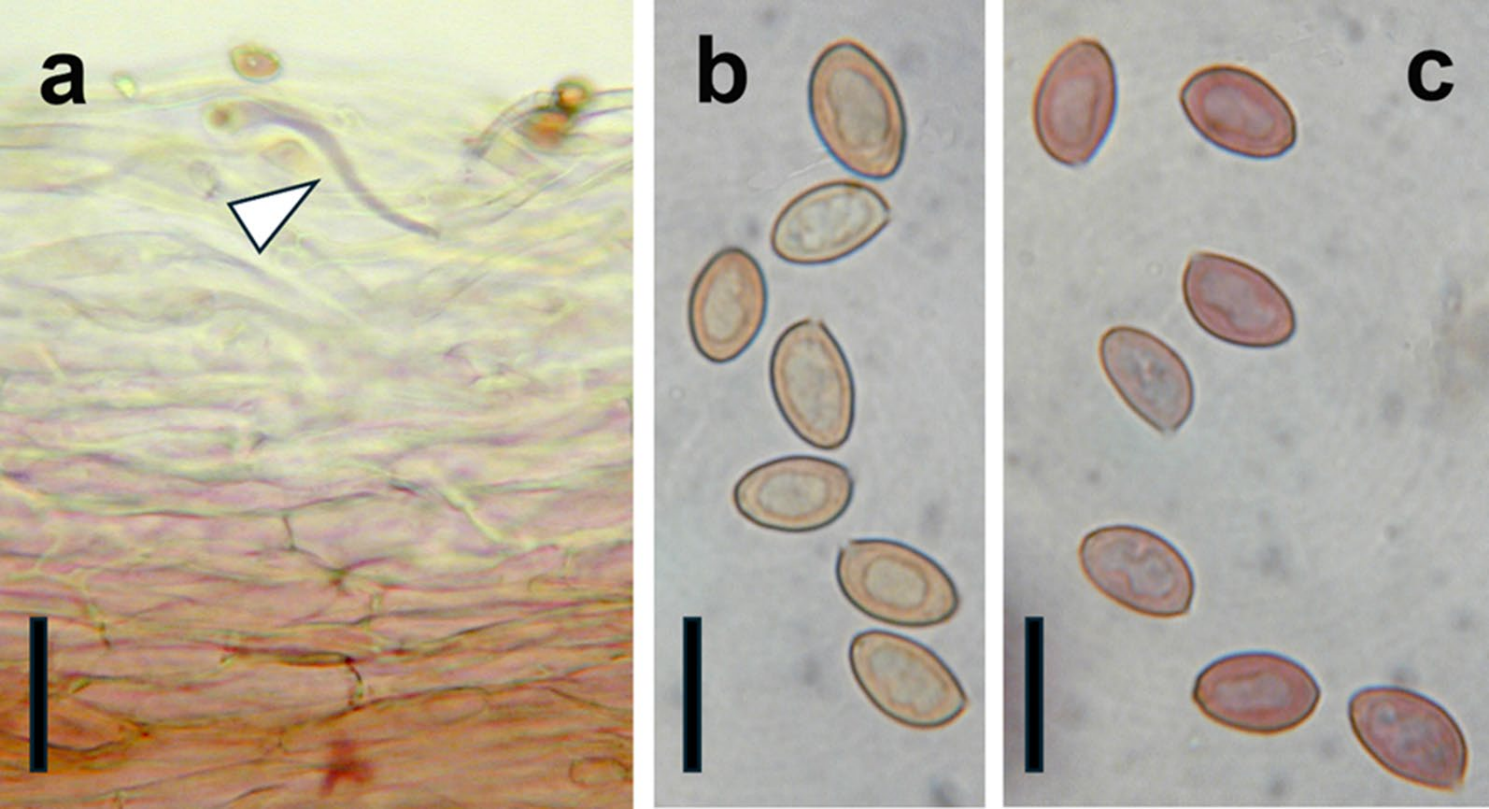
Micrographs of diagnostic features of *Cortinarius mapuveronicae* (CONCF 2241, isotype); **a:** pileipellis in cross section in KOH (5%), some hyphae turned purplish (arrowhead); scale bar = 40 µm; **b:** basidiospores in water, scale bar = 10 µm; **c:** basidiospores turned purplish brown after addition of KOH (5%), scale bar = 10 µm

**Fig. 4:**
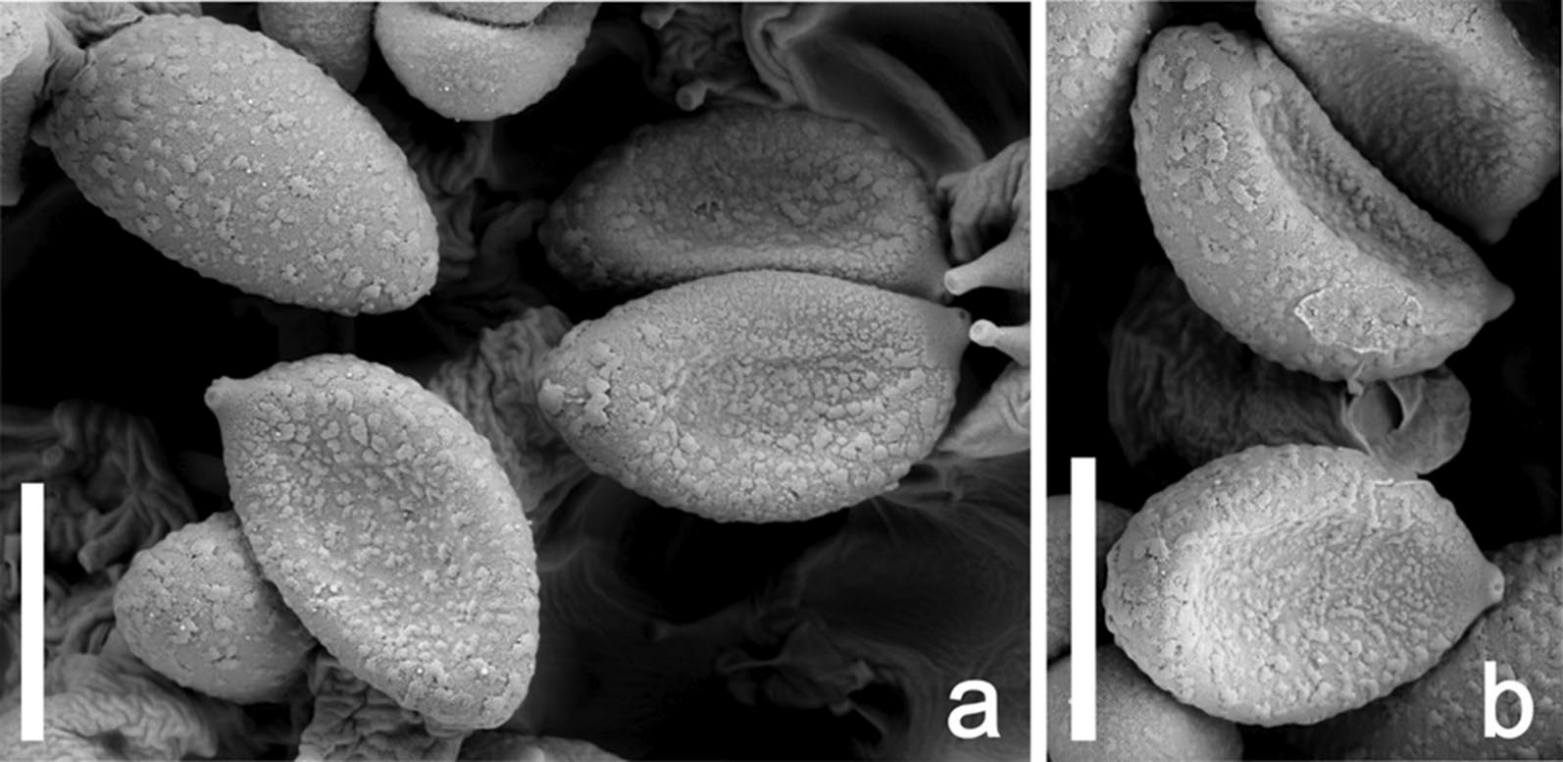
**a**, **b:** SEM pictures of basidiospores of *Cortinarius mapuveronicae* (IBF2023110), holotype; scale bar = 5 µm

**Fig. 5:**
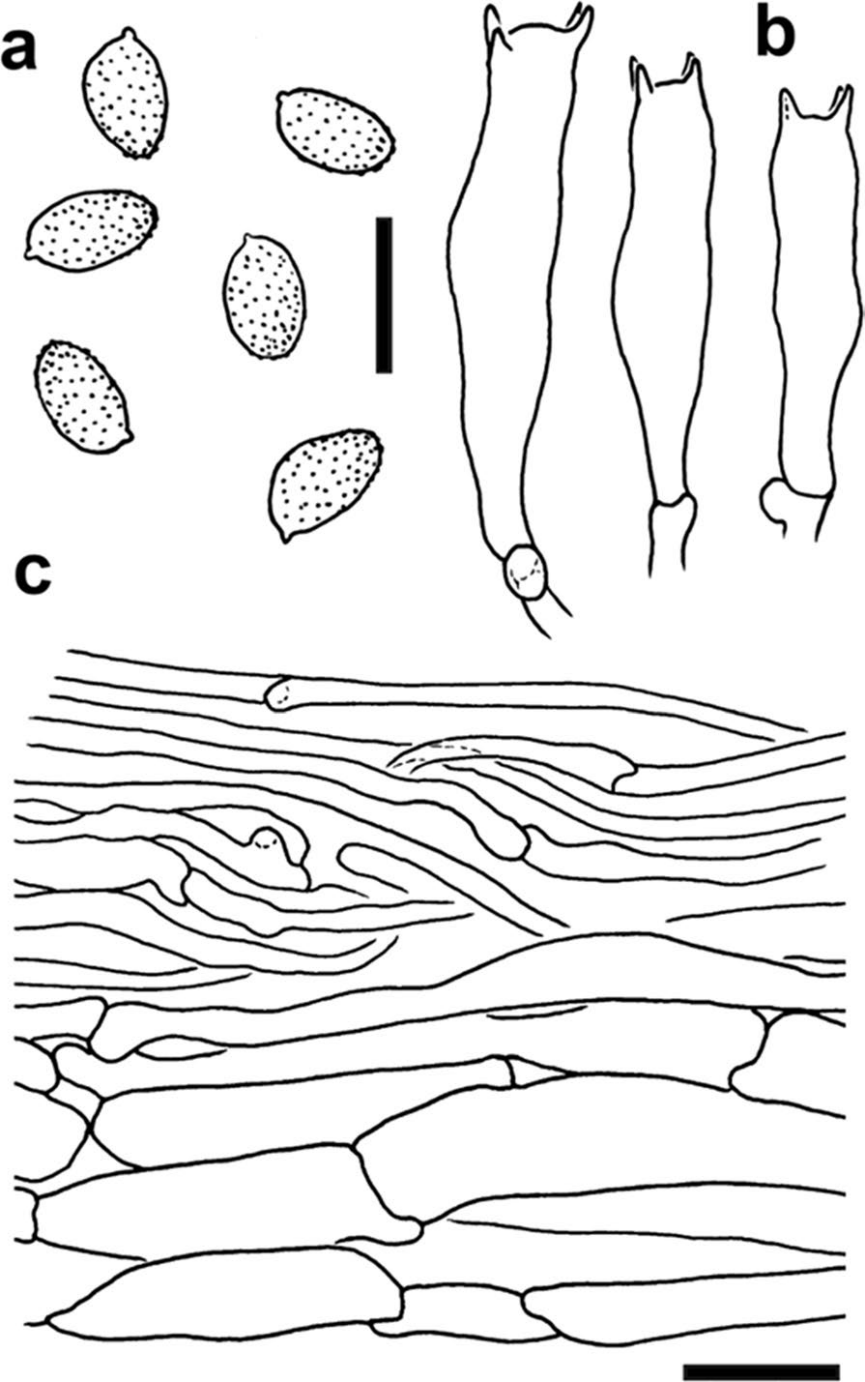
Line drawings of diagnostic features of *Cortinarius mapuveronicae* (CONCF 2241, isotype); **a:** basidiospores; scale bar = 10 µm; **b:** basidia; scale bar = 10 µm; **c:** pileipellis in cross section; scale bar = 20 µm

***Cortinarius mapuveronicae*** N. Arnold, Palfner, Peintner, Huymann, Siewert sp. nov. (Figs. 2, 3, 4, 5)

**Etymology:** In Mapudungun, the traditional native language in southern Chile and Argentina, “mapu” means “land”, circumscribing the native territory, and is a geographical reference to the region where the species was first encountered; the word element “veronicae” was chosen because of the phenotypic similarity to the Australian species *Cortinarius veronicae*.

**Diagnosis:** *C. mapuveronicae* differs from phylogenetically related Australian species by the habitat under *Nothofagus dombeyi* and *Lophozonia obliqua* in Andean-Patagonian forests. The striking orange-red fruitbodies are characteristic for *C.* *mapuveronicae*. *C.* *teresae* and *C.* *rubrobasalis* are other red colored Cortinarii, with red tinge and positive alkaline reaction from this habitat and area, however, the basidiospores of *C.* *mapuveronicae* have a unique, distinctive purplish reaction when observed in the microscope with KOH (3%), which is lacking in *C.* *rubrobasalis* and *C.* *teresae*. Moreover, *C. mapuveronicae* can be distinguished from *C.* *rubrobasalis* by the pale pink or peach-colored to orange lamellae, and by a red to purplish red alkaline reaction, while *C. rubrobasalis* has yellow–brown lamellae when young, and a dark brown to blackish alkaline reaction. *C.* *teresae* has more purplish-red basidiomata when compared to *C.* *mapuveronicae*.

**Type:** Chile, Aysen Region, Chaíten, close to the Carretera Austral (Ruta 7), S 43° 20′ 55.932’’, W 72° 23′ 59.927’’, 635 m asl, mixed forest dominated by *Nothofagus dombeyi*, growing on soil and litter; 09.04.2023; leg.: L. Huymann, U. Peintner, B. Siewert, det.: N. Arnold, G. Palfner L. Huymann, U. Peintner, Holotype IBF20230110, Isotype CONCF 2241; GenBank: PQ859656 (ITS), PX230468 (LSU), PX240688 (*rpb1*).

**Macroscopic description:** *Pileus*: (2.7) 4.3 ± 1.0 (6.5) cm wide; hemispherical to broadly convex, young convex to bluntly conical, margin sometimes enrolled; not hygrophanous, dry and fresh with a greasy shine and texture, innately fibrous, slimy when wet; disc of pileus dark red or bright dark red-brown (S15/S17), sometimes paler red-brown (S13/T13), towards margin bright cherry red (R15/R17); older basidiomata with lighter red margin (P19/P20), rim orange light red (N20/M20) to pale pink or salmon, slightly translucently striate, especially at the margin. *Lamellae*: straight, closely spaced, margin sometimes saw-toothed and wavy (possibly due to drying), young pink to light red (N19/N20) orange (2.5YR 6/8) and even brighter, pink-salmon (N15/N17/N20), old lively orange-brown, edge same color or slightly lighter, slowly turning purple when bruised. *Stipe*: (4.7) 8.1 ± 2.0 (10.8) cm x (1.0) 1.2 ± 0.2 (1.6) cm, cylindrical, slightly club-shaped or spindle-shaped, apex pale beige-pink (K29), silky, lower portion slightly pink-brown (P37), lower two thirds covered with girdles of dark/bright fox-orange-red (R17/P19/P39) velum fibers¸ turning slightly grayish brown–red (P49/N49) upon touch, especially at the base; base lemon yellow with attached yellow mycelium. *Context*: in the cap uniformly pale red-brown (S13/S15), ochre (yellow–red) (N57), stipe soon hollow, light yellow-orange, inside the cavity pink-red and fibrous (N20), also often already decayed in small specimens, slightly pink at the margin (M27); base fiery orange-red (P19), very young olive-green (P65), pale towards the base, slightly yellowing. *Velum*: dark red or fox-red to slightly paler (P19). *Cortina*: pale pinkish white. *Color changes*: flesh in the stipe slowly turning yellow. *Taste*: not specific. *Macrochemical reactions*: with KOH 30% red to purplish magenta-red, on lamellae red–purple, pileus context brown, pileipellis red–purple dark violet. *UV-fluorescence:* negative.

**Microscopic description:** *Basidiospores* (Figs. 3b, 3c, 4, 5a) (8.1) 9.8 ± 0.6 (11.0) × (4.4) 5.8 ± 0.3 (6.6), Q = 1.7 ± 0.1 (n = 171, holotype), (7.9) 9.4 ± 0.6 (11.1) × (4.4) 5.8 ± 0.3 (6.9), Q = 1.6 ± 0.1 (n = 482, all collections), ellipsoid to subamygdaloid, yellowish brown in water, verruculose, almost smooth with light microscopy, more coarsely verrucose towards the apex. *Basidia* (Figs. 3b, 5) clavate, tetrasporic, 28.6–33.0–35.8 × 6.4–7.5–8.6 µm (n = 20). Cheilocystidia or pleurocystidia not observed. *Pileipellis* (Figs. 3a, 5c) consisting of cylindrical, occasionally ramified, hyphae with clamp connections, 3–8 µm in diameter, up to 22 µm in deeper layers. *Chemical reactions:* most basidiospores turning purplish brown in KOH (5%) within about one minute, some hyphae of the pileipellis also turning purplish in KOH.

**Ecology and distribution:** Supposedly ectomycorrhizal, associated with *Nothofagus dombeyi* and *Lophozonia obliqua*. Growing in small groups, terrestrial. So far only recorded in Chile.

**Additional specimens examined:** Chile, Cañete, coord. 37°48′31.3"S, 73°09′38.2"W, leg. N. Arnold, 18.05.2022 (IPB-CHL 43/22); coord. 37°41′23.208''S, 73°21′35.85''W, leg. U. Peintner, L. Huymann, N. Arnold, 18.05.2022 (IBF20220073); coord. 37°49′03.9"S, 73°05′21.5"W, leg. N. Arnold, 28.05.2022 (IPB-CHL 92/22); coord. 37°31′21.7''S, 72°23′58.16''W, leg. J. Farias, 28.05.2022 (CONCF 2069); Caramavida, Arauco Province, coord. 37°41′42.4"S, 73°07′52.7"W, leg. N. Arnold, 08.05.2022 (IPB-CHL 32/22); Curanilahue, Trongol Alto, coord. 37°41′23.208''S, 73°21′35.85''W, leg. U. Peintner, L. Huymann, N. Arnold, 17.05.2022 (IBF20220064); coord. 37°41′23.208''S, 73°21′35.85''W, leg. U. Peintner, L. Huymann, N. Arnold, 21.05.2022 (IBF20220104); coord. 37°33′19.1"S, 73°11′00.1"W, leg. N. Arnold, 21.05.2022 (IPB-CHL 51/22); coord. 37°31′21.72''S, 72°23′58.16''W, leg. J. Farias, 18.06.2022 (CONCF 2088); coord. 37°31′21.7''S, 72°23′58.16''W, leg. J. Farias, 26.06.2022 (CONCF 2088B); La Union, Ranco Province, coord. 40°13′32.1"S, 73°21′36.9"W, leg. N. Arnold, 03.05.2018 (IPB-CHL 9/18); coord. 40°13′32.5"S, 73°21′35.7"W, leg. N. Arnold, 30.04.2022 (IPB-CHL 11/22); coord. 40°13′33.2"S, 73°21′35.4"W, leg. N. Arnold, 13.05.2023 (IPB-CHL 19/23); Llancacura, coord. 40°13′32.916''S, 73°21′35.63''W, leg. U. Peintner, L. Huymann, N. Arnold, 14.05.2022 (IBF20220019); coord. 40°13′32.916''S, 73°21′35.63''W, leg. U. Peintner, L. Huymann, N. Arnold, 14.05.2022 (IBF20220020); Los Álamos, Caramahuida, coord. 37°31′21.7''S, 72°23′58.16''W, leg. N. Arnold, 21.05.2017 (CONCF 1787); Nahuelbuta, Arauco Province, coord. 37°49′38.1"S, 73°00′36.6"W, leg. N. Arnold, 01.05.2018 (IPB-CHL 46/18); coord. 37°49′39.1"S, 73°00′35.6"W, leg. N. Arnold, 26.05.2018 (IPB-CHL 61/18); Chaíten, coord. 43°20′55.932''S, 73°10′18.73''W, leg. L. Huymann, B. Siewert, U. Peintner, 09.04.2023 (IBF20230090); coord. 43°20′48.408''S, 72°23′59.92''W, leg. L. Huymann, B. Siewert, U. Peintner, 09.04.2023 (IBF20230104); coord. 43°20′55.932''S, 73°10′18.73''W, leg. L. Huymann, B. Siewert, U. Peintner, 09.04.2023 (IBF20230110 holotype, CONCF 2241 isotype); coord. 43°20′48.408''S, 72°23′59.92''W, leg. L. Huymann, B. Siewert, U. Peintner, 09.04.2023 (IBF20230111); coord. 43°20′48.408''S, 72°23′59.92''W, leg. L. Huymann, B. Siewert, U. Peintner, 09.04.2023 (IBF20230112);

**Notes:** The species falls in a clade with *C. rubrobasalis* (BPP 1, BS 77), another species from South-America with quite similar red basidiomata. However, the sequences originating from the holotypes of *C. rubrobasalis* (IBF19630173) and *C. mapuveronicae* (IBF20230110) do have 16 differences in the ITS sequences and only 97.22% common identity.

The color change of the basidiospores to purplish brown after addition of KOH is a distinctive feature of *C.* *mapuveronicae* which has rarely been reported from other Cortinarii, such as *C.* *anthracinus* (Fr.) E. Berger [31]. The morphologically similar species *C.* *rubrobasalis* and *C. teresae* do not share this attribute.

*C. rubrobasalis* has a dark brown KOH reaction on the pileus surface, the flesh of the base is immediately turning black [1], whereas *C. mapuveronicae* has a purplish magenta red reaction. *C.* *teresae* has more purplish-red basidiomata when compared to *C.* *mapuveronicae*.

*C. mapuveronicae* has some resemblances to *C. veronicae* K. Soop from New Zealand. This species has more orange-red lamellae, and an alkaline reaction, which is olive-grey on the surface and less purplish magenta red [3, 30], furthermore the basidiospores are more broadly elliptic (5.7–7.7 × 5–5.7 µm), and the habitat on different continents (South America compared to New Zealand / Australia) is clearly distinctive.

### Isolation and structural elucidation of compounds 1–10

Repeated column chromatography of the crude extract resulting from combined collections of *C. mapuveronicae* (Supplementary Information: Additional file 1, Tab. S3) on Sephadex LH 20 and Sephadex LH 60, Polyamide SC 6-Ac, silica gel 60, RP18 modified silica gel, and SPE on dimethyl-modified silica gel yielded anthraquinoid compounds **1**–**10**.

Compound (**1**) was isolated as red amorphous solid. Its high-resolution negative-ion ESIMS spectrum exhibited a quasi-molecular ion peak at *m/z* 343.0462 ([M − H]^−^ (calculated for C_17_H_11_O_8_^−^, 343.0532), consistent with the formula C_17_H_12_O_8_ and corresponding to 12 degrees of unsaturation. (Supplementary Information: Additional file 1, Fig. S2).

**Fig. 6:**
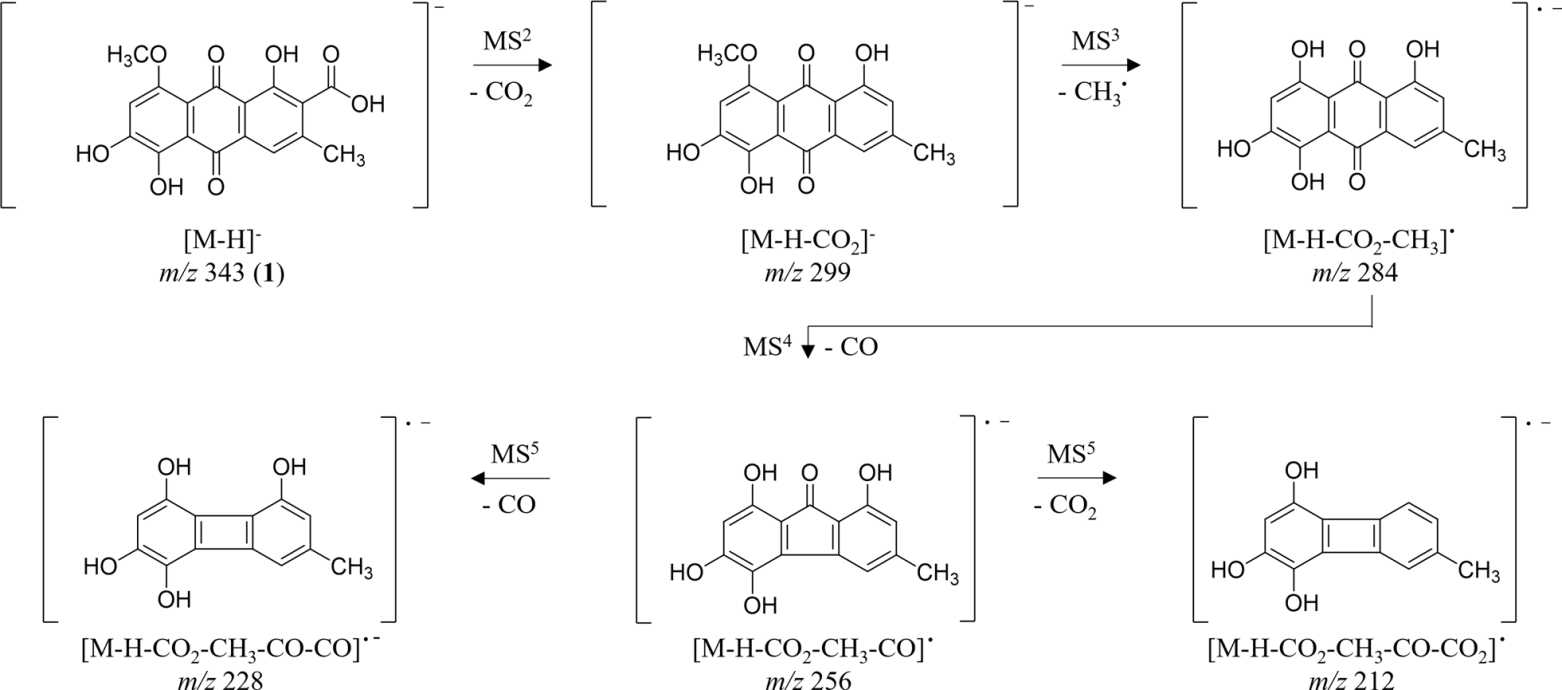
Key ions in the negative HRESIMS^n^ spectra of clavorubin-8-*O*-methylether (**1**)

HRESIMS^n^ analysis of **1** in negative mode displayed characteristic ions at *m/z* 299 [M − H − CO_2_]^−^and *m/z* 284 [M − H − CO_2 _− CH_3_]^●−^ correlating to losses of the carboxyl and a radical methyl (Fig. 6). Further fragmentation is characterized by losses of CO and CO_2_ (*m/z* 256 [M − H − CO_2 _− CH_3 _− CO]^●−^, *m/z* 240 [M − H − CO_2_− CH _3 _− CO_2_]^●−^, *m/z* 228 [M − H − CO_2 _− CH_3 _− CO − CO]^●−^, and *m/z* 212 [M − H − CO_2 _− CH_3 _− CO − CO_2_]), similar to emodin (**3**) as reported in literature [32].

**Fig. 7:**
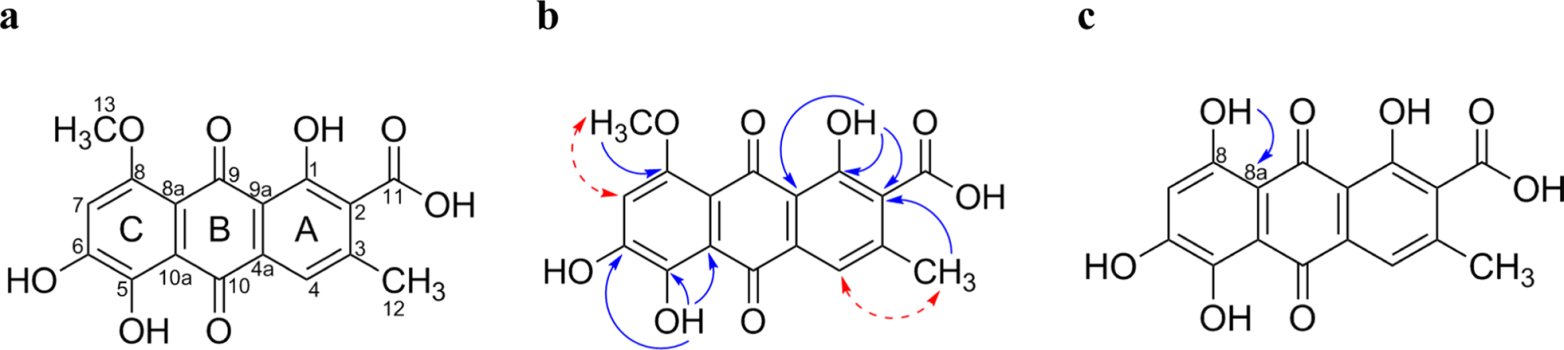
**a:** Structure of clavorubin-8-*O*-methylether (**1**); **b:** key HMBC (plain arrow) and NOE (dashed arrow) correlations of clavorubin-8-*O*-methylether (**1**); **c:** key HMBC (arrow) of clavorubin (**11**)

The structure of **1** was further determined on the basis of detailed NMR analysis (Table 1; Figs. 7 a – b; Supplementary Information: Additional file 1, Figs. S3–S7). The ^1^H NMR spectrum of **1** show resonances from two phenolic hydroxy groups (*δ*_H_ 14.03, s, OH; 13.48, s, OH), two isolated signals from two aromatic methine groups (*δ*_H_ 7.57, s, H-4; *δ*_H_ 6.94, s, H-7), and resonances of a methyl group attached to a carbon (*δ*_H_ 2.42, s, H-12, *δ*_C_ 20.0, C-12) and a methyl group connected to oxygen (*δ*_H_ 3.91, s, H-13; *δ*_C_ 56.7, C-13). The ^13^C NMR data complements these assignments and in particular confirms the presence of two quinonoid carbonyls (*δ*_C_ 189.4, C-9; 186.0, C-10) and one carboxyl (*δ*_C_ 167.4, C-11) in the molecule. The placement of the substituents in the rings of the anthraquinone scaffold could be deduced from ^1^H-^13^C HMBC and NOE experiments. The ^1^H-^13^C HMBC spectrum shows in accordance with NOE correlations cross peaks of H-4 with C-3, OH-1 with C-1, C-9a, and C-2. Further ^1^H-^13^C HMBC cross peaks of H-4 to C-2, C-11, C-12, and H-12 to C-2, C-3, and C-4 confirmed the substitution pattern of ring A. Ring B is characterized by two carbonyl moieties at C-9 and C-10, and four quaternary carbons C-4a, C-8a, C-9a, and C-10a. NOE correlation of H-7 to H-13 and ^1^H-^13^C HMBC cross peaks of the hydroxyl group OH-5 with C-5, C-6 and C-10a finalized the substitution pattern in ring C. In addition, the position of the methoxy group at C-8 was supported by a demethylation reaction of **1** to clavorubin (**11**), which shows ^1^H-^13^C HMBC cross peaks of the resulting hydroxy OH-8 to C-8a (Fig. 6c; Table 1; Supplementary Information: Additional file 1, Fig. S49). The OH-6 resonances in **1** could not be observed in the ^1^H NMR spectrum but is determined through the chemical shift of C-6 in ^13^C NMR spectrum as well as by HMBC cross peaks of OH-5 to C-6. The obtained spectral data of the semisynthetic compound **11** were in agreement with those reported by Gill et al. [33]. Therefore, the structure of **1** was recognized as a new natural acidic anthraquinone methylether of clavorubin (**11**) and consequently named clavorubin-8-*O*-methylether (**1**).

**Table 1:** NMR spectroscopic data (500/125 MHz, THF-*d*_8_ and DMSO-*d*_6_, *δ* in ppm) of **1** and** 11**

**Pos**	**1**			**11**			
	*δ*_C_, type^a^	*δ*_H_ (*J* in Hz)^a^	HMBC^a^	*δ*_C_, type^a^	*δ*_H_ (*J* in Hz)^b^	*δ*_H_ (*J* in Hz)^a^	HMBC^a^
1	160.6, C–OH	14.03, s, 1H	1, 2, 3, 9a	160.1, C–OH	12.65, s, 1H	12.63, s, 1H	1, 2, 9a
2	131.9, C			132.0, C			
3	143.4, C			144.9, C			
4	119.5, CH	7.57, s, 1H	2, 3, 9, 9a, 10, 11	121.0, CH	7.73, s, 1H	7.71, s, 1H	1, 2, 3, 9a, 10, 11, 12
4a	132.9, qC			133.8, qC			
5	156.2, C–OH	13.47, s, 1H	5, 6, 10a	158.5, C–OH	13.34, s, 1H	12.49, s, 1H	7, 8, 8a
6	148.3, C–OH		^c^	150.5, C–OH	^c^	^c^	
7	108.7, CH	6.94, s, 1H	5, 6, 7, 8, 8a, 10	111.2, CH	6.76, s, 1H	6.66, s, 1H	5, 6, 8, 8a, 9
8	158.9, C			162.2, C–OH	12.49, s, 1H	13.44, s, 1H	5, 6, 10a
8a	110.4, qC			106.2, qC			
9	189.4, C			189.0, C			
9a	116.4, qC			115.6, qC			
10	186.0, C			187.6, C			
10a	116.1, qC			113.5, qC			
11	167.4, C		^c^	167.1, C	^c^	^c^	
12	20.0, C	2.42, s, 3H	1, 2, 3, 4, 11	20.2, C	2.51, s, 3H	2.46, s, 3H	1^d^, 2, 3, 4, 9a^d^, 11^d^
13	56.7, C	3.91, s, 3H	8	–	–	–	–

^a^Measured in THF-*d*_8_

^b^Measured in DMSO-*d*_6_

^c^Not observed

^d^Weak ^1^H-^13^C-HMBC cross peaks

The UV spectrum of clavorubin-8-*O*-methylether (**1**) exhibits four maxima [UV (MeOH) λ_max_ = 205 nm, 237 nm, 262 nm, 490 nm (Supplementary Information: Additional file 1, Fig. S8)]. Spotted on TLC, clavorubin-8-*O*-methylether (**1**) appears as an orange-red spot showing no fluorescence under UV-light (λ = 366 nm). By treating the spot with ammonia vapor a bathochromic shift to violet can be observed (Bornträger reaction; Supplementary Information: Additional file 1, Figs. S1 a–d). Due to the high occurrence of **1** (18.3 mg isolated from 212 g d.w. fruiting bodies), clavorubin-8-*O*-methylether (**1**) is the most prominent pigment in *C. mapuveronicae*.

**Fig. 8:**
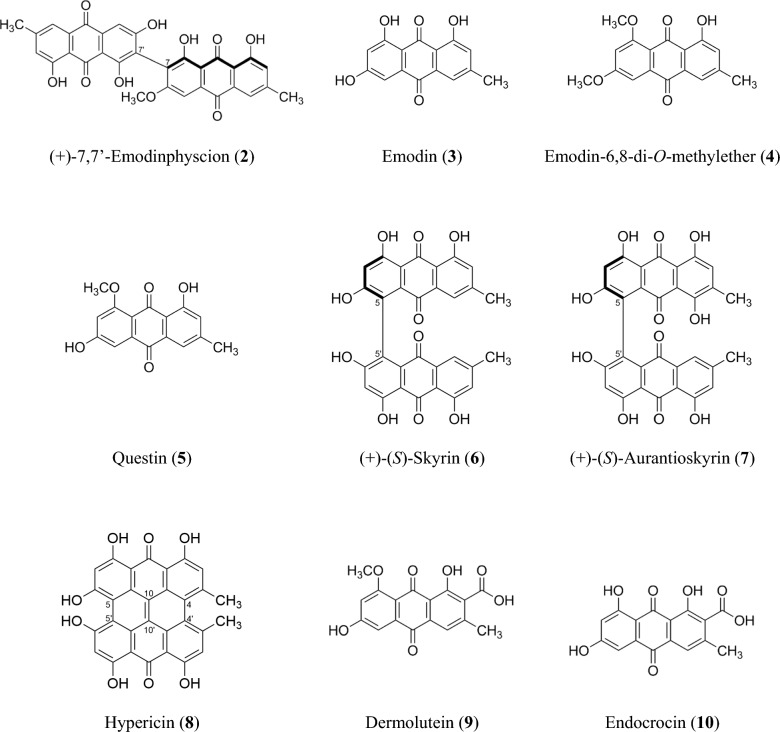
Structures of isolated compounds **2**–**10**

**Fig. 9:**
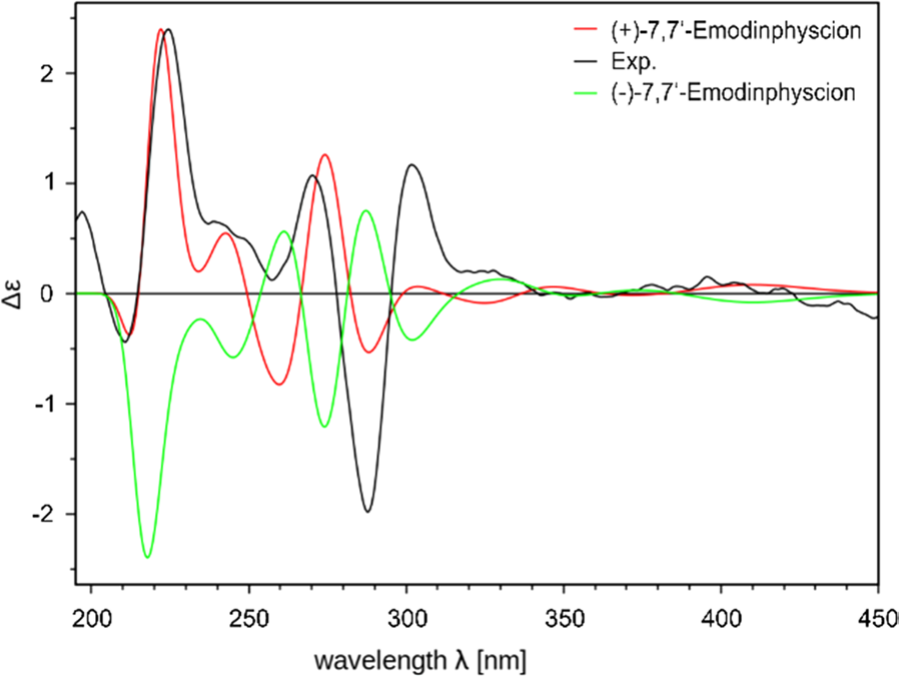
Calculated ECD spectra of the (+)-7,7’-emodinphyscion (**2**, red) and (−)-7,7’-emodinphyscion (green) isomers in comparison with the experimental spectra (black)

**Fig. 10:**
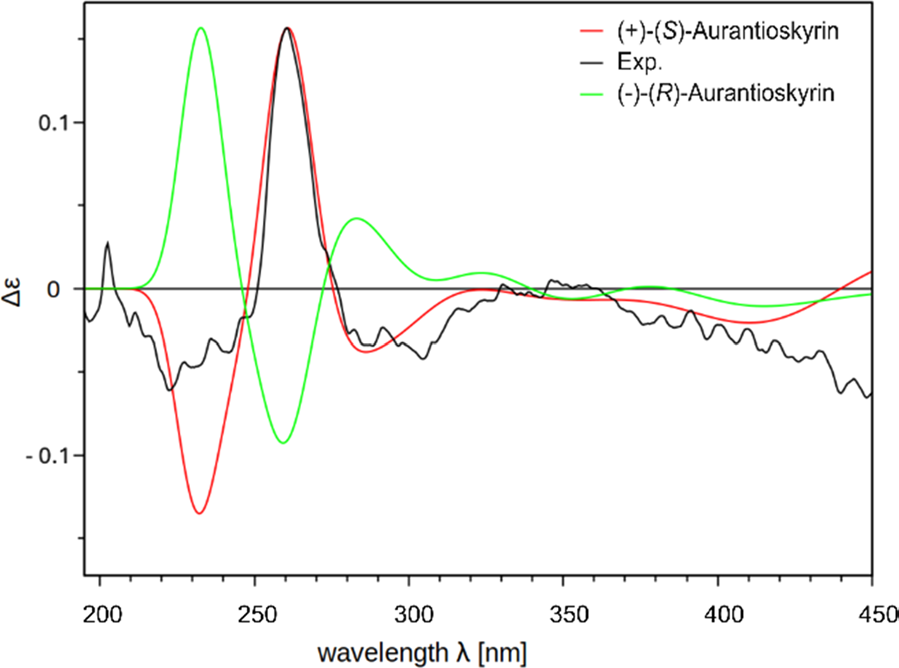
Calculated CD spectra of (+)-(*S*) aurantioskyrin (**7**, red) and (–)-(*R*)*-*aurantioskyrin (green) isomers in comparison with the experimental spectra (black)

Isolated compounds **2**–**10** (Fig. 7) were identified based on their spectroscopic data (^1^H NMR, ^13^C NMR, HRESIMS^n^, CD; Supplementary Information: Additional file 1, Figs. S9-S49, Tab. S2) and comparison by TLC with authentic reference compounds as ( +)-7,7-emodinphyscion (**2)** [29], emodin (**3**) [34], emodin-6,8-di-*O*-methylether **(4)** [35], questin (**5**) [36], ( +)-(*S*)-skyrin (**6**) [37], ( +)-(*S*)-aurantioskyrin (**7**) [38], hypericin (**8**) [39], dermolutein (**9**) [40], and endocrocin (**10**) [40]. The obtained spectral data of the isolated compounds **2**–**10** were in agreement with those reported in the relevant references. The comparative analysis of the calculated and experimental electronic circular dichroism (ECD) spectra of compounds **2** and **7** showed a strong agreement between the experimental ECD spectrum and the calculated spectrum of ( +)-7,7’-emodinphyscion (Fig. 8, 9), with a similarity factor of S = 0.72 (σ = 0.21 eV at 18 nm shift) and ( +)-(*S*)-aurantioskyrin (Fig. 9, 10), with a similarity factor of S = 0.8372 (σ = 0.3 eV at 31 nm shift), respectively.

### Photoantimicrobial activity of isolated compounds 1–10

The isolated compounds were photobiologically evaluated employing a recently established protocol [41]. All experiments were performed as biological triplicates of technical triplicates. As depicted in Table 2, next to the known photoantimicrobial active compound from dermocyboid Cortinarii emodin (**3**) [42], (+)-(*S*)-aurantioskyrin (**7**), and hypericin (**8**) were found. Furthermore, (+)-(*S*)-skyrin (**6**), showed significant light-enhanced activity, although a PhotoMIC (photoinduced minimal inhibitory concentration) could not be determined in the tested concentration range c_max_ = 5 mg/L.

**Table 2:** PhotoMIC [mg/L] of compounds **1**–**10** against the human pathogens *S. aureus*, and *C. albicans* observed under dark and irradiated (λ = 478 nm; H = 30 J/cm^2^) conditions in broth micro-dilution assay. (see Supplementary Information: Additional file 1, Fig. S53)

Compound	*S. aureus*		*C. albicans*	
	Dark (MIC)	Irradiated (PhotoMIC)	Dark (MIC)	Irradiated (PhotoMIC)
Clavorubin-8-*O*-methylether (**1**)	–	–	–	–
(+)-7,7´-Emodinphyscion (**2**)	–	–	–	–
Emodin (**3**)	–	0.5	–	2.5
Emodin-6,8-di-*O*-methylether (**4**)	–	–	–	–
Questin (**5**)	–	–	–	–
(+)-(*S*)-Skyrin (**6**)	–	–	–	–
(+)-(*S*)-Aurantioskyrin (**7**)	–	5	–	–
Hypericin (**8**)	–	2.5	–	–
Dermolutein (**9**)	–	–	–	–
Endocrocin (**10**)	–s	–	–	–
Fluconazole	n.d	n.d	0.5	n.d
Chloramphenicol	15	n.d	n.d	n.d

“n.d.” = not determined; “- “ not active under test conditions (c_max_ = 5 µg/mL)

In the next step, the photocytotoxicity was examined, employing cell cultures of two different human cancer cell lines. The test was based on a modified sulforhodamine B (SRB) assay [43], as published elsewhere [44]. In short, the cells were treated with different test concentrations 24 h after seeding and incubated for 24 h. After the incubation time, fresh media were supplied and one of two identically prepared plates was irradiated (λ = 478 nm, H = 30 J/cm^2^, t_PI_ = 30 min). Cell fixation followed by SRB staining was performed after an additional 48 h. As depicted in Table 3, photocytotoxic activity was observed for emodin (**3**), (+)-(*S*)-aurantioskyrin (**7**), and hypericin (**8**).

**Table 3:**
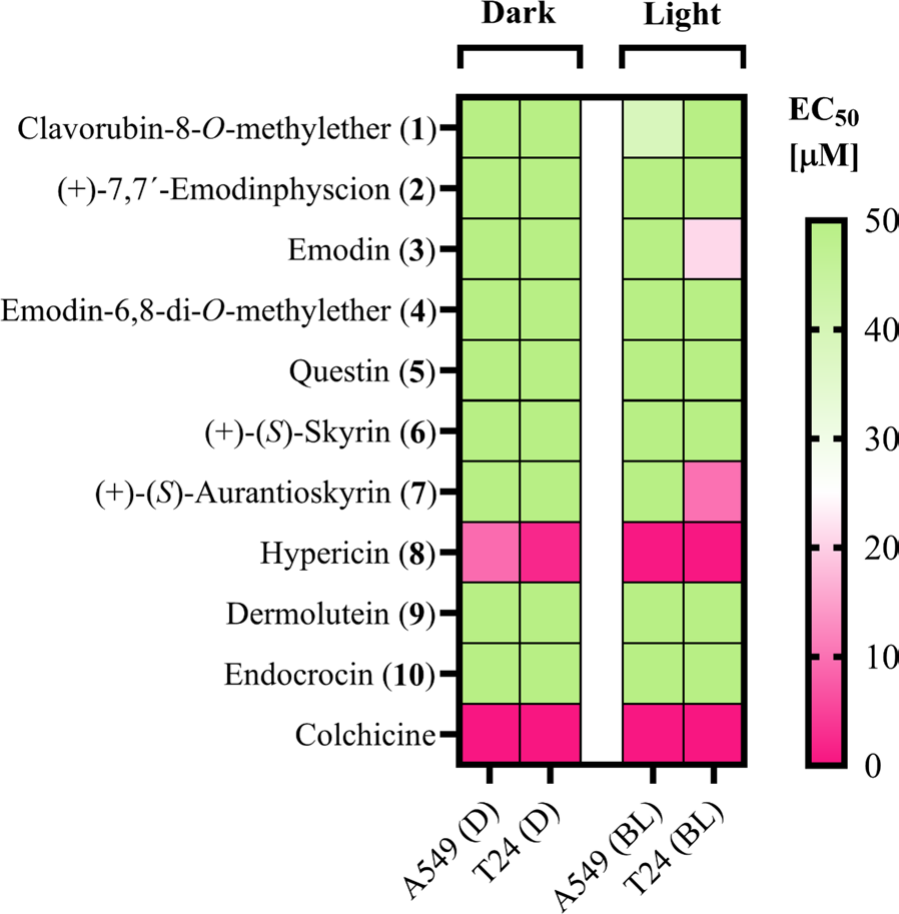
Cell viability of T24 and A549 carcinoma cells under blue light irradiation (BL; λ = 478 nm; H = 5.2 J/cm^2^) and dark condition (D) given as EC_50_ (Supplementary Information: Additional file 1, Fig. S52, Tab. S5)

## Discussion

Our concatenated phylogenetic analysis (see Supplementary Information: Additional file 1, Fig. S50) places the lineage of the two South American sister taxa *C. mapuveronicae* and *C. rubrobasalis* in a large, weakly supported dermocyboid group (BPP 0.69). This group was already detected in an earlier study [3]. Our results also confirm earlier hypotheses of a possible common origin of dermocyboid species, as this group contains both, Southern hemisphere lineages (sections Orixanthi, Icterinula, Walkeri, Chrysmata, Cruentoides, Pauperae, Rubrobasales, Ignelli) and Northern hemisphere lineages (sections Dermocybe, Olivaceopicti). Some Southern hemisphere species without clear affiliation to a defined section, like *C. promethenus*, *C. cramesinus*, *C. elaiops*, *C.* *aurantioferreus*, *C. mycenarum*, and *C. cardinalis* also belong to this large group which is characterized by small dermocyboid basidiomata and positive alkaline reactions, although anthraquinonoid pigments were reported only for few of them [1, 27, 33, 38, 40].

*Cortinarius mapuveronicae* has a sister group relationship to the holotype of *C. rubrobasalis*. The sequencing of the type of *C. rubrobasalis* allowed to correct a taxonomical error in the database: The sequence deposited as *C. rubrobasalis* TUB11487 has 58 base differences in the ITS region when compared to the sequence from the type of *C. rubrobasalis* IBF1963173 but is identical to *C. teresae* IBF20230155, which forms a lineage with *C. carneolus* IPB-CH 41/17, TUB011471. Unfortunately, this lineage was wrongly named sect. Rubrobasales based on the misidentification and needs to be renamed in future studies.

As already proposed by Soop and colleagues, Northern Hemisphere dermocyboid Cortinarii fall into Sect. *Dermocybe*, which has a distinct evolutionary history clearly differing from all Southern Hemisphere lineages [23]. Moreover, our analysis also indicates a separate evolutionary history of South-American dermocyboid Cortinarii: they are closely related, but do not form mixed lineages with Southern hemisphere species from Australia or New Zealand. However, species sampling is still comparatively poor, when compared to dermocyboid taxa from the Northern Hemisphere.

The orange-red major anthraquinoid pigment **1** is most likely responsible for the red–orange color of the basidiomata of *C. mapuveronicae* and could be identified as the new naturally occurring clavorubin-8-*O-*methylether (**1**) based on the intensive mass spectrometric and NMR spectroscopic investigations. Clavorubin-8-*O*-methylether (**1**) represents the pigment chemical link of *C.* *mapuveronicae* to the Australian *Dermocybe* spp. via the core structure of clavorubin (**11**) [33]. Clavorubin (**11**) was first isolated from *Claviceps purpurea* by Frank and co-workers [45, 46] and later recognized in *Cortinariu*s sp. WAT 24273 and *Cortinarius* sp. WAT 26645 [33]. We were allowed to sequence material of *Cortinarius* sp. WAT 26645 and could identify it as *Cortinarius veronicae* (see Supplementary Information: Additional file 1, Tab. S1).

Besides clavorubin-8-*O-*methylether (**1**)*,* a series of known monomeric and dimeric anthraquinones could be isolated from *C. mapuveronicae* in variable amounts. The yellow neutral anthraquinones emodin (**3**), emodin-6,8-di-*O*-methylether (**4**), and questin (**5**) were present in *C. mapuveronicae* in small quantities. Whereby the precursor of the neutral anthraquinone series emodin (**3**) occurs in species from all four continental areas [25–29, 38, 39, 47–49], emodin-6,8-di-*O*-methylether (**4**) and questin (**5**) could be only detected in species from Europe so far. [38]

The neutral dimeric anthraquinones (+)-7,7'-emodinphyscion (**2**), (+)-(*S*)-skyrin (**6**), and (+)-(*S*)-aurantioskyrin (**7**) could be isolated as minor pigments from *C. mapuveronicae*. (+)-7,7'-emodinphyscion (**2**) [28] seems to be restricted to dermocyboid Cortinarii originating from Patagonia, whereas (+)-(*S)*-aurantioskyrin (**7**) could be isolated so far only from the European phlegmacioid *Cortinarius atrovirens* [38]. (+)-(*S*)-Skyrin (**6**) is recognized in dermocyboid Cortinarii from Patagonian South America [28, 29] and Australasia [27, 29, 38, 50], and some European phlegmacioid *Cortinarius* species [38].

As representatives of the monomeric acidic anthraquinones, dermolutein (**9**) and endocrocin (**10**) are present in *C. mapuveronicae*. The pigments dermolutein (**9**) and endocrocin (**10**) occur in several European species [38] and in the Australian *C. cardinalis* [40]. It can be suggested, that the enzymatic *O*-methylation in position 8 of the anthraquinone core seems to be more likely in the dermocyboid *C. mapuveronicae* through the occurrence of dermolutein (**9**), clavorubin-8-*O*-methylether (**1**), and questin (**5**). The naphtodianthrone hypericin (**8**) was obtained in small quantities from *C. mapuveronicae*. The occurrence of hypericin (**8**) seems to be restricted to species from Patagonian South America and Australasia and could so far not be detected in European and North American dermocyboid Cortinarii [51–54].

As a remarkable finding, anthraquinones connected to saccharides, mainly in the form of 1-β-D-glucopyranoside, known from European and North American species [38, 42, 48, 49, 54], could not be detected in the Patagonian *C. mapuveronicae.* Furthermore, anthraquinone disaccharides are also absent in *C. mapuveronicae* and have only been found in Australian dermocyboid Cortinarii [55]. The phylogenetic similarity of *C.* *mapuveronicae* and *C.* *rubrobasalis* has not been verified by pigment-chemical investigations of the latter.

To explore the functional implications of these pigments, we investigated whether the photoantimicrobial activity observed in Northern Hemisphere and Australian specimens arises exclusively from the known fungal photosensitizer emodin (**3**) and the hypericin (**8**), or if other pigment classes contribute synergistically to this trait.

In detail, a (photo)antimicrobial and (photo)cytotoxic assay were performed on all isolated compounds (Table 3) The (photo)antimicrobial assay is based on the CLSI and EUCAST microdilution protocol and thus the MIC obtained can be well correlated with the values published by EUCAST. As reference antibiotic chloramphenicol was chosen, which has a MIC of 15 mg/L (c = 46.6 µM) against the strain and is therefore still susceptible, and as antifungal agent fluconazole, which has a MIC of 0.5 mg/L (c = 1.6 µM). Highly potent candidates were identified with a photo-activated minimum inhibitory concentration (PhotoMIC) of 0.5 (1.9 µM), 5 (c = 9.0 µM) and 2.5 (5.0 µM) mg/L for **3**, **7** and **8**, respectively. A cut-off test concentration of c = 5 mg/L was chosen to allow identification of only truly active compounds within the range of potent antimicrobials.

A similar trend was observed in the (photo) cytotoxicity screen, confirming the photo-enhanced effect of these fungal anthraquinones. The modest photo-active effect of **3** can be attributed to the chosen light source; the peak wavelength of the irradiation setup used was λ = 478 nm (FWHM = 27 nm). The absorption maximum of **3** in a polar solvent (methanol) was characterized as λ_max_ (ε) = 436 (10,724). Consequently, the probability of absorption is lower under the chosen setup and explains the observed photobiological effect.

The uncovering of the newly identified fungal photosensitizer ( +)-(*S)*-aurantioskyrin (**7**) proves that the photoantimicrobial property is not only due to known light-activated pigments, but that distinctly different photosensitizers have evolved in the South American dermocyboid Cortinarii. Phylogenetic patterns reveal distinct lineages, and closely related species do have similar pigment patterns [27]. However, pigment chemistry as detected in dermocyboid Cortinarii suggests more diverse pathways of biosynthesis of anthraquinonoid secondary metabolites which seem to be correlated with geographical regions and specific lineages on both hemispheres.

## Experimental

### General experimental procedures

Column chromatography was performed on Sephadex LH 20 (Fluka, Germany), Sephadex LH 60 (Fluka, Germany), silica gel (0.040–0.063 mm, Merck, Germany), C18 modified silica gel (LiChroPrep RP18, 40–63 μm, Merck, Germany), and Polyamide SC 6-Ac (Macherey & Nagel, Germany). For solid phase extraction Chromabond C2 cartridges (Macherey & Nagel, Germany) were used. Analytical TLC was performed on pre-coated silica gel F_254_ aluminum sheets (Merck, Germany) using toluene/ethyl formate/formic acid, 10:5:3 (v/v) as solvent system. Compound spots were analyzed by their absorbance at long-wavelength UV light (*λ* = 366 nm) and short-wavelength UV light (*λ* = 254 nm), as well as their color reaction on TLC by treating with ammonia vapor.

Analytical and semi-preparative RP-HPLC were performed on a Shimadzu prominence system consisting of an SPD-M20A diode array detector, a FRC-10A fraction collector, a CBM-20A communications bus module, a DGU-20A5R degassing unit, an LC-20AT liquid chromatograph, and an SIL-20A HT auto sampler. Chromatographic separation was performed using a semi-preparative Agilent Zorbax Eclipse XDB C18 column (ID 10.0 mm, length 250 mm, particle size 5 µm) using ultrapure water (TKA ultrapure water system) and acetonitrile as eluents both acidified with 0.1% formic acid.

UV spectra were recorded on a Jasco V-560 UV/Vis spectrophotometer and CD spectra were obtained from a Jasco J-815 CD spectrophotometer. The specific rotation was measured with a Jasco P-2000 polarimeter.

^1^H, ^13^C and 2D NMR spectra were recorded on a Bruker Avance Neo 500 NMR spectrometer at 500.234 and 125.797 MHz, respectively, using a 5 mm prodigy probe with TopSpin 4.0.7 and TopSpin 4.3.0 spectrometer software. For 2D NMR standard pulse sequences (hsqcedetgpsisp2.3, hmbcgplpndqf, roesyphpp.2 and noesygpphpp) were used.

^1^H chemical shifts are referenced to internal TMS (^1^H *δ* = 0.0 ppm), ^13^C chemical shifts to THF-*d*_8_ (^13^C *δ* = 67.4 ppm), pyridine-*d*_5_ (^13^C *δ* = 123.5 ppm) and DMSO-*d*_6_ (^13^C *δ* = 39.7 ppm).

The negative ion high-resolution ESI mass spectra were obtained from an Orbitrap Elite mass spectrometer (Thermo Fisher Scientific, Bremen, Germany) equipped with an HESI electrospray ion source (spray voltage 3.0 and 4.0 kV, capillary temperature 275 °C, source heater temperature 40 °C; FTMS resolution 60.000). Nitrogen was used as sheath gas. The sample solutions were introduced continuously via a 500 μL Hamilton syringe pump with a flow rate of 5 μL min–1. The instrument was externally calibrated by the Pierce® LTQ Velos ESI positive ion calibration solution (product number 88323) and Pierce® ESI negative ion calibration solution (product number 88324) from Thermofisher Scientific, Rockford, IL, 61,105 USA). The data were evaluated by the Xcalibur software 2.7 SP1 and 2.2 SP1.

The incubation step of the DNA extractions was done with an Eppendorf Thermomixer comfort (Eppendorf AG, Germany), and the centrifugation with an Eppendorf centrifuge 5415 R (Eppendorf AG, Germany). For the PCRs a Thermocycler Peqlab Primus 96 advanced (Peqlab Biotechnologie GmbH, Erlangen, Germany) was used. The results of the PCR were checked with an electrophorese chamber RunOne Casting System (Embi Tec, San Diego, California, USA) and the Bio Rad Gel Doc EZ Imager 1,708,270 together with the Bio Rad Blue sample tray (Bio-Rad Laboratories, Hercules, California, USA).

Samples of emodin (**3**), emodin-6,8-di-*O*-methylether (**4**), questin (**5**), (+)-(*S*)-skyrin (**6**), (+)-(*S*)-aurantioskyrin (**7**), hypericin (**8**), dermolutein (**9**), and endocrocin (**10**) originating from Steglich’s group (Ludwig-Maximilians-University Munich, Germany), were available from the *in-house* compound library of the Department of Bioorganic Chemistry, Leibniz Institute of Plant Biochemistry, Halle (Saale), Germany.

### Fungal material

For a list of all specimens, and their voucher, GenBank and collection dates, examined in this study see Supplementary Information: Additional file 1, Tab. S1. Basidiomata of *C.* *mapuveronicae* were collected in Chile, Biobío Region, Arauco Province, Cordillera de Nahuelbuta, and Los Rios Region, Ranco Province, ascent to Reserva Costera Valdiviana, growing on soil and leaf litter beneath *Nothofagus dombeyi* (Mirb.) Oerst and *Lophozonia obliqua* (Mirb.) Heenan & Smissen. Voucher specimens are deposited at the Fungarium of Concepción University (CONCF) in Concepcion, Chile, in the Fungarium of the Tiroler Landesmuseen (IBF), Hall, Austria, and at the Leibniz Institute of Plant Biochemistry (IPB), Halle, Germany.

### Morphological analysis

All features were noted from the fresh basidiomata and the colors were described after the “Code des Couleurs” [56] and the *“*Munsell Soil Color Chart” [57]. For macro-chemical reactions KOH 30% was used. Possible fluorescence under UV was observed at wavelengths 250 nm and 350 nm with dry material. Light microscopy was carried out with a Nikon Eclipse 600 with fresh material or dried material soaked in water or KOH 3% before visualization. Measurements were carried out using the imaging software Nis-Elements D (Nikon Europe B.V.). Basidiospores were measured in KOH 3% with a 100 × oil immersions objective. At least 30 spores were measured per collection, and 170 of the holotype, in order to allow for statistical evaluation of spore size. The length/width ratio Q was calculated. Measurements are presented in the following: (min) MV ± *sd* (max) x (min) MV ± *sd* (max) Q value ± *sd* (n = x). For basidiospore sizes of all measured collections see Supplementary Information: Additional file 1, Tab. S4. All other microscopic attributes were analyzed from isotype material (CONCF 2241/ IBF20230110) with a Leitz Dialux compound microscope. For the SEM pictures of the spores a piece of lamellae was dried with a BAL-TEC CPD 030 Critical point dryer. After mounting, they were gold sputtered using a BAL-TEC MED 020 Coating system and he images were taken using a Zeiss DSM 982 Gemini Scanning Electron Microscope.

### Molecular analysis

DNA extraction was done with the CTAB method using dried material [58, 59]. A Polymerase chain reaction (PCR) was done with different primers for the rDNA ITS1–5.8S–ITS2 sequences the primers ITS1 and ITS4 [60] was used. The rDNA LSU region was amplified with the primer combination LR0R and LR7 [60]. The amplifications of RPB1 domain were made with the Cortinarius specific primer combination RPB1-cort119bF and RPB1-cort92b6R [61]. Sequences were assembled and edited with Geneious [62]. As a first step, a Blast search was conducted in UNITE (https://unite.ut.ee/) and GenBank (http://ncbi.nlm.nih.gov/) and sequences of closely related Cortinarius species or with similar morphology were then downloaded. A total of 19 ITS sequences were 5 LSU and 3 rpb1 sequences generated in this study. The newly generated sequences were submitted to GenBank (Supplementary Information: Additional file 1, Tab. S1).

### Phylogenetic analysis

The different loci (ITS; LSU; rpb1) were aligned with the online version of MAFFT 7 [63] for ITS and rpb1 loci with the E-INS-i algorithm and for the LSU locus with G-INS-i algorithm, all under default settings. Then they were checked and adjusted in Geneious Prime 2025.0.3.

Two final datasets were created: The first multigene alignment was made to show the deeper relationships to other *Cortinarius* species. Therefore, all three single loci alignments (ITS, LSU, rpb1) were concatenated. The second one, with only selected species and two loci (ITS, LSU) served for a better delimitation of species. As outgroup for the first dataset (ITS, LSU, *rpb1*), sect. Austroduracinus with *C.* *austroduracinus* and *C. viscincisus* was chosen [3]. *Cortinarius veronicae* was chosen as outgroup for the second dataset (ITS, LSU analysis).

For each of the datasets, a ML tree was calculated using RAxML [64]. To further evaluate branch robustness a Bayesian interference tree was calculated using MrBayes [65]. ML analysis was carried out based on the best model (first dataset = GTR + G, second dataset = GTR + G + I), rapid Bootstrap analyses were conducted with 1000 replications. For each dataset, two separate MrBayes runs were run under the general time-reversible model with gamma-distributed rate variation. Runs included four incrementally heated chains, they were run for 5 million generations each, sampling every 100 generations and with the first 1.25 million generations discarded as burn-in.

### Pigment extraction and isolation

Air-dried fruiting bodies of *C. mapuveronicae* (coll. IPB-CHL 9/18, 46/18, 61/18, 11/22, 32/22, 43/22, 51/22, 92/22, 19/23 (Supplementary Information: Additional file 1, Tab. S3), combined 212 g, were crushed using a blender and exhaustively extracted with acidified acetone (4 × 1 L) in an ultrasound bath for 1 h at room temperature. The combined orange-brown solution was evaporated *in vacuo* to dryness. The resulting crude extract (11.37 g) was dissolved in water and partitioned with ethyl acetate (5 × 300 mL). The resulting ethyl acetate fractions were combined and evaporated to dryness. Separation of the ethyl acetate extract (5.54 g) by gel-permeation column chromatography on Sephadex LH 20 (eluent acetone/methanol 4:1 (v/v) afforded six fractions (F1–F6). Only fractions showing a positive Bornträger reaction on TLC were further processed (Supplementary Information: Additional file 1, Fig. S51).

Fraction F2 was separated by adsorption column chromatography on Polyamide SC 6-Ac using a series of solvents with increasing polarity (*n*-hexane, ethyl acetate, acetone, methanol) as eluents, yielding four fractions (F2a–F2d). Fraction F2b was subjected to silica gel column chromatography using toluene/ethyl acetate/chloroform (2:1:2 (v/v)) as elution system and afforded fraction F2b1 containing **2** (6.9 mg), and fractions F2b2–F2b3. Fraction F2b2 was further separated by gel-permeation column chromatography on Sephadex LH 20 (eluent methanol) and yielded compounds **4** in a pure form (2.1 mg), and **3** and** 5** impure. Therefore, semi-preparative HPLC (0 → 20 min, 5 → 100% B for **3** and **5** individually) was carried out to afford **3** (*t*_R_ = 16.2–16.5 min, 4.4 mg) and **5** (*t*_R_ = 14.7–15.1 min, 1.4 mg). Likewise, fraction F2b3 was separated accordingly on Sephadex LH 20 (eluent methanol) and afforded compounds **6** (6.0 mg) and **7** (0.8 mg).

Fraction F4 was separated by gel-permeation column chromatography on Sephadex LH 60 (eluent methanol) resulting in four fractions (F4a–F4d). Fraction F4c was subjected to solid phase extraction on a C2-cartridge using mixtures of ethyl acetate and methanol (increasing polarity) as eluents to afford **8** (5.4 mg).

Fraction F5 was separated by gel-permeation column chromatography on Sephadex LH 20 (eluent methanol/dichloromethane 1:1 (v/v)) and yielded compounds **9** (3.6 mg) and **10** (3.2 mg).

Fraction F6 was separated by adsorption column chromatography on Polyamide SC 6 Ac, eluting with solvents with increasing polarity (*n*-hexane, ethyl acetate, acetone, methanol) resulting in four fractions (F6a–F6d). Fraction F6d was subjected to adsorption chromatography on C18 modified silica gel using water/methanol (isocratic, 7:3 (v/v)) as eluent to obtain **1** (18.3 mg).

### Spectroscopic data of isolates

*Clavorubin-8-O-methylether* (**1**): yellow-orange amorphous solid; TLC *R*_f_ 0.41 (toluene/ethyl formate/formic acid, 10:5:3 (v/v)); UV (MeOH) λ_max_ (log *ε*) 205 (3.85), 237 (3.91), 262 (3.85), 313 (3.54), 490 (3.45) nm; HRESIMS *m/z* 343.0462 ([M − H]^−^, calcd for C_17_H_11_O_8_^−^, 343.0532; Scheme 1). ^1^H NMR, ^13^C NMR, HSQC and HMBC data see Table 1 and Supplementary Information: Additional file 1, Figs. S2-S8. Purity:^1^H NMR based: 87.7%.

(+)-*7,7’-Emodinphyscion* (**2**): dark yellow amorphous solid; TLC *R*_f_ 0.64 (toluene/ethyl formate/formic acid, 10:5:3 (v/v)); $[\alpha]_{D}^{24}$ + 2.53 (*c* 0.08, MeOH); UV (MeOH) λ_max_ (log ε) 219 (3.86), 283 (3.52), 443 (3.21) nm; CD (c 0.04 mM, MeOH) λ_max_ (Δε) 210 (−0.43), 224 (+ 2.39), 257 (+ 0.12), 270 (+ 1.07), 288 (− 1.98), 301 (+ 1.16) M^−1^ × cm^−1^; HRESIMS *m/z* 551.0995 ([M − H]^−^, calcd for C_31_H_19_O_10_^−^, 551.1056). ^1^H NMR and ^13^C NMR data see Supplementary Information: Additional file 1, Figs. S9-S12. Purity: ^1^H NMR based: 86.4%.

*Emodin* (**3**): orange amorphous solid; TLC *R*_f_ 0.74 (toluene/ethyl formate/formic acid, 10:5:3 (v/v)); UV (MeOH) *λ*_max_ (log *ε*) 203 (4.01), 221 (4.05), 254 (3.82), 289 (3.77), 437 (3.52) nm; HRESIMS *m/z* 269.0458 ([M − H]^−^, calcd for C_15_H_9_O_5_^−^, 269.0528). ^1^H NMR and ^13^C NMR data see Supplementary Information: Additional file 1, Figs. S13-S16. Purity: ^1^H NMR based: 88.7%.

*Emodin-6,8-di-O-methylether* (**4**): yellow amorphous solid; TLC *R*_f_ 0.74 (toluene/ethyl formate/formic acid, 10:5:3 (v/v)); UV (MeOH) *λ*_max_ (log *ε*) 224 (4.10), 279 (3,82), 422 (3.44) nm; HRESIMS *m/z* 297.1535 ([M − H]^−^, calcd for C_17_H_13_O_5_^−^, 297.0841). ^1^H NMR and ^13^C NMR (HSQC/HMBC) data see Supplementary Information: Additional file 1, Figs. S17-S20. Purity: ^1^H NMR based: 91.2%.

*Questin* (**5**): yellow amorphous solid; TLC *R*_f_ 0.60 (toluene/ethyl formate/formic acid, 10:5:3 (v/v)); UV (MeOH) *λ*_max_ (log *ε*) 217 (3.18), 285 (2.83), 438 (2.35) nm; HRESIMS *m/z* 283.0613 ([M − H]^−^, calcd for C_16_H_11_O_5_^−^, 283.0685). ^1^H NMR and ^13^C NMR (HSQC/HMBC) data see Supplementary Information: Additional file 1, Figs. S21-S24. Purity: ^1^H NMR based: 87.9%.

(+)*-*(*S*)-*Skyrin* (**6**): orange amorphous solid; TLC *R*_f_ 0.54 (toluene/ethyl formate/formic acid, 10:5:3 (v/v)); $[\alpha]_{D}^{24}$ + 52.5 (*c* 0.022 mg/mL, MeOH); UV (MeOH) *λ*_max_ (log *ε*) 219 (3.30), 257 (3.11), 297 (2.92), 450 (2.74) nm; CD (c 0.04 mM, MeOH) λ_max_ (Δε) 215 (-0,33), 246 (-0,20), 261 (+ 0,91), 308 (-0,21) M^−1^ × cm^−1^; HRESIMS *m/z* 537.0826 ([M − H]^−^, calcd for C_30_H_17_O_10_^−^, 537.0900). ^1^H NMR and ^13^C NMR data see Supplementary Information: Additional file 1, Figs. S25-S28. Purity: ^1^H NMR based: 92.6%.

( +)*-*(*S*)-*Aurantioskyrin* (**7**): orange amorphous solid; TLC *R*_f_ 0.54 (toluene/ethyl formate/formic acid, 10:5:3 (v/v)); $[\alpha]_{D}^{24}$ + 3.5 (*c* 0.08 mg/mL, MeOH); UV (MeOH) *λ*_max_ (log *ε*) 255 (2.84), 301 (2.61), 468 (2.35) nm; CD (c 1.4 mM, MeOH) λ_max_ (Δε) 222 (-0,06), 260 (+ 0,16), 304 (-0.04), 346 (+ 0.01) M^−1^ × cm^−1^; HRESIMS *m/z* 553.0774 ([M-H]^−^, calcd for C_30_H_17_O_10_^−^, 554.0849). ^1^H NMR and ^13^C NMR (HSQC/HMBC) data see Supplementary Information: Additional file 1, Figs. S29-S33. Purity: ^1^H NMR based: 66.3%.

*Hypericin* (**8**): black amorphous solid; TLC *R*_f_ 0.52 (toluene/ethyl formate/formic acid, 10:5:3 (v/v)); UV (MeOH) *λ*_max_ (log *ε*) 216 (4.39), 285 (4.15), 327 (4.02), 383 (3.62), 450 (3.67), 471 (3.71), 509 (3.52), 546 (3.90), 588 (4.20) nm; HRESIMS *m/z* 503.0768 ([M − H]^−^, calcd for C_30_H_15_O_8_^−^, 503.0845). ^1^H NMR and ^13^C NMR (HSQC/HMBC) data see Supplementary Information: Additional file 1, Figs. S34-S37. Purity: ^1^H NMR based: 94.6%.

*Dermolutein* (**9**): yellow-orange amorphous solid; TLC *R*_f_ 0.41 (toluene/ethyl formate/formic acid, 10:5:3 (v/v)); UV (MeOH) *λ*_max_ (log *ε*) 227 (4.03), 275 (3.85), 426 (3.42) nm; HRESIMS *m/z* 327.0509 ([M − H]^−^, calcd for C_17_H_11_O_7_^−^, 327.0583). ^1^H NMR and ^13^C NMR data see Supplementary Information: Additional file 1, Figs. S38-S40. Purity: ^1^H NMR based: 81.9%.

*Endocrocin* (**10**): yellow-orange amorphous solid; TLC *R*_f_ 0.48 (toluene/ethyl formate/formic acid, 10:5:3 (v/v UV (MeOH) *λ*_max_ (log *ε*) 227 (3.81), 257 (3.57), 274 (3.63), 442 (3.27) nm; HRESIMS *m/z* 313.0358 ([M − H]^−^, calcd for C_16_H_9_O_7_^−^, 313.0427). ^1^H NMR and ^13^C NMR (HSQC/HMBC) data see Supplementary Information: Additional file 1, Figs. S41-S44. Purity: ^1^H NMR based: 83.6%.

*Clavorubin* (**11**): The orange-red monomethylether clavorubin-8-*O*-methylether **1 (**9.2 mg, 3.0 μmol) was heated with pyridinium chloride (1.71 g, 14.7 mmol) at 180 °C for 6 h in a 10 mL round-bottom flask. The red mass was cooled and digested with ultrapure water (10 mL). After centrifugation of the reaction mixture, the precipitate was separated and dissolved in 5% aqueous sodium carbonate solution. The resulting dark purple solution was acidified with *conc*. HCl until the reaction solution changes color to orange and extracted with ethyl acetate (3 × 20 mL). The organic layers were combined and separated by gel-permeation column chromatography on Sephadex LH 20 (eluent acetone/methanol 4:1 (v/v)) and yielded clavorubin (**11**) [5.8 mg (63%); *R*_f_ 0.58 (toluene/ethyl formate/formic acid, 10:5:3 (v/v)); HRESIMS *m/z* 329.0291 ([M-H]^−^, calcd for C_16_H_9_O_8_^−^, 329.0376)], ^1^H NMR, ^13^C NMR, HSQC and HMBC data see Supplementary Information: Additional file 1, Figs. S46-S49. Purity: ^1^H NMR based: 91.8%.

### Computational details

The molecular geometries of the compounds (+)-7,7’-emodinphyscion (**2**) and (+)-(*S*)-aurantioskyrin (**7**) were sampled using an in-house script. Starting from a SMILES string, the script generated 50 different structural conformers for both structures. These conformers were minimized with the steepest decent algorithm using the MMFF94 force field and ranked based on their total energies [66]. For **2** the best two force field energy minimum structures were saved as SDF file. These structures were further optimized using density functional theory (DFT) with the CAM-B3LYP [67] functional and the def2-TZVP [68] basis set. For **7** the best force field energy minimum structure was saved as SDF file and the stereoisomeric version of it was manually generated. Solvent effects for methanol were considered using the conductor-like polarizable continuum model (CPCM) [69] as implemented in the ORCA 5.0 [70] program package. Electronic Circular Dichroism (ECD) spectra were computed for each optimized structure using time-dependent DFT (TD-DFT) at the same level of theory (CAM-B3LYP/def2-TZVP) by calculating the first 40 excited states. The calculated ECD spectra were compared with experimental data using the SpecDis software (version 1.71) [71] with a Gaussian distribution function at a half-bandwidth of σ = 0.3 eV, allowing for a spectral shift in the range of + 50 to − 50 nm.

### Photodynamic inhibition (PDI) of microbial growth

All experiments and preparations for PDI were conducted under aseptic conditions within a biosafety level-2 laminar airflow cabinet at room temperature. The study examined the antibacterial and antifungal efficacy of selected compounds against *Candida albicans* (IBF19991201) and *Staphylococcus aureus* (DSM1104), sourced from the Leibniz Institute—DSMZ-German Collection of Microorganisms and Cell Cultures GmbH, Braunschweig, Germany. Strains were stored in darkness at 4 °C until use. Bacteria were cultured on lysogeny broth (LB) agar, while *C. albicans* was maintained on potato dextrose agar (PDA). DMSO, LB-agar from Sigma Aldrich (Merck KGaA, Darmstadt, Germany, Batch #018K0809), and RPMI-1640 medium (Lot: 1003383838) were purchased from Merck (Merck KGaA, Darmstadt, Germany); PDA and Mueller–Hinton broth (MHB) from VWR (MHB Lot: 1934, PDA Lot: 241,310,184, VWR International GmbH, Vienna, Austria). Each PDI experiment involved a freshly prepared overnight culture incubated at 37 °C for 18–23 h in darkness. Each PDI experiment involved a freshly prepared overnight culture incubated at 37 °C for 18–23 h in darkness.

### Photo minimal inhibitory concentration (MIC) assay

Photoantimicrobial experiments followed established protocols [41]. Eleven compounds were tested, with chloramphenicol and fluconazole as positive controls. Identical 96-well plates were prepared for dark and light treatments, containing six dilution levels (0.06–5.00 µM), medium blanks, fraction blanks, and untreated growth controls. Inoculum turbidity was adjusted to a McFarland standard of 0.5 (1.5 × 10⁸ CFU/mL) using spectrophotometry at 600 nm (bacteria) [72] and 530 nm (yeast) [73]. Bacterial cultures were prepared in Müller Hinton Broth (MHB), while yeast was cultivated in double-strength RPMI-1640 media. Within 30 min of inoculum preparation, plates were inoculated (50 μL for bacteria, 100 μL for yeast) to achieve standard CFU concentrations ((2–8) × 10^5^ CFU/mL for bacteria and (0.5–2.5) × 10^5^ CFU/mL for yeast) [73, 74]. After 30 min of incubation in darkness, one plate was exposed to a light dose of 30 J/cm^2^ (λ = 459.6 nm, E = 33.7 mW/cm^2^, t = 14.8 min) using a LabLights Millu 1–96 Microplate Illuminator (prototype of LabLights, Innsbruck, Austria). Both light-treated and control plates were incubated at 37 °C for 24 h in darkness. Following incubation, plates were homogenized using a microplate mixer and absorbance was measured (bacteria: 600 nm, fungi: 530 nm) with a SpectraMax^®^ PLUS 384 spectrophotometer (Molecular Devices LLC., San Jose, CA, USA). The inhibitory effects were determined by comparing treated wells to uninhibited controls, with blank corrections applied. All conditions were tested in technical and biological triplicates, and data were analyzed using GraphPad Prism 8.0.1 (GraphPad Software, Boston, MA, USA).

### (Photo)cytotoxicity evaluation

Cytotoxicity assessments were performed on A549 (non-small cell lung cancer, ATCC, Sigma-Aldrich) and T24 (urinary bladder carcinoma, CLS, Eppelheim) cell lines. Cells were cultured in Dulbecco's Modified Eagle Medium (DMEM, Gibco, 11,520,556) supplemented with 8.93% fetal bovine serum (FBS, Eximus, BS-2020–500), 0.893 mM sodium pyruvate (Pyr, Gibco, 11,360,070), and 89.3 U/mL penicillin–streptomycin (P/S, Biotech, P06-07100), with routine passaging at 80% confluency. The assay followed established protocols, with cells seeded into 96-well plates at 2000 cells/well and incubated at 37 °C with 5% CO₂ for 24 h [44, 75]. Test compounds (11 in total) were dissolved in DMSO (10 mM stock) and diluted to the test concentrations (0.5–50 μM). After a 24 h treatment period, media were aspirated, replaced, and one of two identical plates was irradiated with 5.5 J/cm^2^ (λ = 468 nm, E = 6.2 mW/cm^2^, t = 14.8 min), while the other was kept in darkness. Plates were further incubated for 48 h towards achieving a 96 h total incubation for the whole assay. Cells were fixed with 10% trichloroacetic acid and stained with 0.057% sulforhodamine B (Acid red 52, TCI, 3520-42-1) [43]. After washing and drying, 200 µL of 10 mM tris(hydroxymethyl)aminomethane (TRIS, Carl Roth, 77-86-1) were placed in each well to redissolve the staining agent. After 30 min of incubation and 1 min of double orbital shaking, light absorbance at 510 ± 0.8 nm was read out using a Tecan Spark^®^ 10 M plate-reader (Tecan Trading AG, Männedorf, Switzerland). EC₅₀ values were calculated using GraphPad Prism 8.0.1 (GraphPad Software, Boston, MA, USA) with a 95% confidence interval.

### Statistical analysis and visualization programs

Statistical analyses were performed in R Version 4.3.3 [76]. For comparing of Q-values and spore sizes, a Bonferroni t-test was applied [77]. For the scatterplot of the spore sizes, the package ggplot2 [78] was used. For creating the figures Inkscape 1.3 was used [79].

## Supplementary Information

Supplementary material 1.

## Data Availability

The raw spectroscopic data for compounds **1**–**11** are deposited at RADAR [80]. Nomenclatural and taxonomic data of new taxa are deposited in MycoBank under the number MB858613. Newly generated sequences are deposited at GenBank, under the accession numbers PQ859656 (ITS), PX230468 (LSU), PX240688 (rpb1).
