# Supplementary material for: Cortinarius mapuveronicae from South America, a chemical and morphological link between European and Australian dermocyboid Cortinarii
Source: Nat Prod Bioprospect. 2026 Feb 2;16(1):22. doi: 10.1007/s13659-025-00552-5 (PMC12862044; doi:10.1007/s13659-025-00552-5)
Supplement: Supplementary file 1 — Supplementary material 1. [file 13659_2025_552_MOESM1_ESM.docx]

***Cortinarius mapuveronicae* from South America, a chemical and morphological link between European and Australian dermocyboid Cortinarii**

Josefine Lange^1+^, Lesley Huymann^2+^, Sophie Schwarzkopf ^3^, Dilara Balci^1^, Mehdi D. Davari^1^, Arijana Turanovic^3^, Clemens Gotsis^2^, Götz Palfner^4^, Bianka Siewert^3, 5^, Ursula Peintner^2*^, and Norbert Arnold^1*^

^1^Department of Bioorganic Chemistry, Leibniz Institute of Plant Biochemistry, Weinberg 3, D-06120 Halle (Saale), Germany

^2^Department of Microbiology, University Innsbruck, Technikerstr. 25, 6020 Innsbruck, Austria

^3^Institute of Pharmacy, University of Innsbruck, Innrain 80-82, 6020 Innsbruck, Austria

^4^Departamento de Botanica, Facultad de Ciencias Naturales y Oceanograficas, Universidad de Concepción, Casilla 160-C, Concepción, Chile

^5^current address: Institute of Pharmacy, University of Hamburg, Bundesstr. 45, 20146 Hamburg, Germany

*Corresponding authors Norbert Arnold (Norbert.Arnold@ipb-halle.de); Ursula Peintner (Ursula.Peintner@uibk.ac.at)

^+^first authors (equal contribution)

ORCID

Josefine Lange ORCID 0009-0005-1049-5603

Lesley Huymann ORCID 0000-0002-0483-0031

Dilara Balci ORCID 0009-0008-8159-5443

Sophie Schwarzkopf ORCID 0009-0003-9530-0179

Mehdi D. Davari ORCID 0000-0003-0089-7156

Clemens Gotsis ORCID 0000-0001-6847-7970

Götz Palfner ORCID 0000-0001-6098-728X

Bianka Siewert ORCID 0000-0002-4910-1756

Ursula Peintner ORCID 0000-0001-5388-4266

Norbert Arnold ORCID 0000-0003-2925-2263

**Supplementary Information**

**Table of Contents**

| **Fig. S1** | TLC of ethylacetate extracts and isolated compounds (**1-10**) |
| --- | --- |
| **Fig. S2** | Negative ion HRESIMS^n^ spectra of clavorubin-8-*O*-methylether (**1**) |
| **Fig. S3** | ^1^H NMR spectrum (500 MHz, THF-*d*_8_) of clavorubin-8-*O*-methylether (**1**) |
| **Fig. S4** | ^13^C NMR spectrum (125 MHz, THF-*d*_8_) of clavorubin-8-*O*-methylether (**1**) |
| **Fig. S5** | HSQC spectrum (500/125 MHz, THF-*d*_8_) of clavorubin-8-*O*-methylether (**1**) |
| **Fig. S6** | HMBC spectrum (500/125 MHz, THF-*d*_8_) of clavorubin-8-*O*-methylether (**1**) |
| **Fig. S7** | NOESY spectrum (500 MHz, THF-*d*_8_) of clavorubin-8-*O*-methylether (**1**) |
| **Fig. S8** | UV spectrum of clavorubin-8-*O*-methylether (**1**) |
| **Fig. S9** | Negative ion HRESIMS^n^ spectra of (+)-7,7’-emodinphyscion (**2**) |
| **Fig. S10** | ^1^H NMR spectrum (500 MHz, pyridine-*d*_5_) of (+)-7,7’-emodinphyscion (**2**) |
| **Fig. S11** | ^13^C NMR spectrum (125 MHz, pyridine-*d*_5_) of (+)-7,7’-emodinphyscion (**2**) |
| **Fig. S12** | CD spectrum of (+)-7,7-emodinphyscion (**2**) |
| **Fig. S13** | Negative ion HRESIMS^n^ spectra of emodin (**3**) |
| **Fig. S14** | ^1^H NMR spectrum (500 MHz, CDCl_3_) of emodin (**3**) |
| **Fig. S15** | HSQC spectrum (500/125 MHz, CDCl_3_) of emodin (**3**) |
| **Fig. S16** | HMBC spectrum (500/125 MHz, CDCl_3_) of emodin (**3**) |
| **Fig. S17** | Negative ion HRESIMS^n^ spectra of emodin-6,8-di-*O*-methylether (**4**) |
| **Fig. S18** | ^1^H NMR spectrum (500 MHz, CDCl_3_) of emodin-6,8-di-*O*-methylether (**4**) |
| **Fig. S19** | HSQC spectrum (500/125 MHz, CDCl_3_) of emodin-6,8-di-*O*-methylether (**4**) |
| **Fig. S20** | HMBC spectrum (500/125 MHz, CDCl_3_) of emodin-6,8-di-*O*-methylether (**4**) |
| **Fig. S21** | Negative ion HRESIMS^n^ spectra of questin (**5**) |
| **Fig. S22** | ^1^H NMR spectrum (500 MHz, CDCl_3_) of questin (**5**) |
| **Fig. S23** | HSQC spectrum (500/125 MHz, CDCl_3_) of questin (**5**) |
| **Fig. S24** | HMBC spectrum (500/125 MHz, CDCl_3_) of questin (**5**) |
| **Fig. S25** | Negative ion HRESIMS^n^ spectra of (+)-(*S*)-skyrin (**6**) |
| **Fig. S26** | ^1^H NMR spectrum (500 MHz, DMSO-*d*_6_) of (+)-(*S*)-skyrin (**6**) |
| **Fig. S27** | ^13^C NMR spectrum (125 MHz, DMSO-*d*_6_) of (+)-(*S*)-skyrin (**6**) |
| **Fig. S28** | CD spectrum of (+)-(*S*)-skyrin (**6**) |
| **Fig. S29** | Negative ion HRESIMS^n^ spectrum of (+)-(*S*)-aurantioskyrin (**7**) |
| **Fig. S30** | ^1^H NMR spectrum (500 MHz, CDCl_3_) of (+)-(*S*)-aurantioskyrin (**7**) |
| **Fig. S31** | HSQC spectrum (500/125 MHz, CDCl_3_) of (+)-(*S*)-aurantioskyrin (**7**) |
| **Fig. S32** | HMBC spectrum (500/125 MHz, CDCl_3_) of (+)-(*S*)-aurantioskyrin (**7**) |
| **Fig. S33** | CD spectrum of (+)-aurantioskyrin (**7**) |
| **Fig. S34** | Negative ion HRESIMS^n^ spectra of hypericin (**8**) |
| **Fig. S35** | ^1^H NMR spectrum (500 MHz, DMSO-*d*_6_) of hypericin (**8**) |
| **Fig. S36** | HSQC spectrum (500/125 MHz, DMSO-*d*_6_) of hypericin (**8**) |
| **Fig. S37** | HMBC spectrum (500/125 MHz, DMSO-*d*_6_) of hypericin (**8**) |
| **Fig. S38** | Negative ion HRESIMS^n^ spectra of dermolutein (**9**) |
| **Fig. S39** | ^1^H NMR spectrum (500 MHz, MeOD) of dermolutein (**9**) |
| **Fig. S40** | ^13^C NMR spectrum (125 MHz, MeOD) of dermolutein (**9**) |
| **Fig. S41** | Negative ion HRESIMS^n^ spectra of endocrocin (**10**) |
| **Fig. S42** | ^1^H NMR spectrum (500 MHz, MeOD) of endocrocin (**10**) |
| **Fig. S43** | HSQC spectrum (500/125 MHz, MeOD) of endocrocin (**10**) |
| **Fig. S44** | HMBC spectrum (500/125 MHz, MeOD) of endocrocin (**10**) |
| **Fig. S45** | Negative ion HRESIMS^n^ spectra of clavorubin (**11**) |
| **Fig. S46** | ^1^H spectrum (500 MHz, DMSO-*d*_6_ and THF-*d*_8_) of clavorubin (**11**) |
| **Fig. S47** | ^13^C NMR spectrum (125 MHz, THF-*d*_8_) of clavorubin (**11**) |
| **Fig. S48** | HSQC spectrum (500/125 MHz, THF-*d*_8_) of clavorubin (**11**) |
| **Fig. S49** | HMBC spectrum (500/125 MHz, THF-*d*_8_) of clavorubin (**11**) |
| **Fig. S50** | Maximum likelihood phylogenetic tree of ITS, LSU, and rpb1 data for the placement of the new species. |
| **Fig. S51** | Isolation scheme. |
| **Fig. S52** | Cell growth inhibition curves of the active anthraquinoid compounds **1**, **3**, **7**, and **8** |
| **Fig. S53** | Growth inhibition curves of the active anthraquinoid compounds **3**, **7**, and **8** |
|  |  |
| **Table S1** | Collections examined in this study |
| **Table S2** | Key ions in the negative HRESIMS^2^ (^a^) and HRESIMS^3^ (^b^) spectra of the anthraquinones (**2-11** |
| **Table S3** | Collections of *C. mapuveronicae* used for pigment-chemical studies |
| **Table S4** | Basidiospore sizes of measured *C. mapuveronicae* collections |
| **Table S5** | EC_50_ values of the isolated compounds **1**-**10** tested against A549 and T24 cells under dark (D) and blue light (BL) irradiation conditions. Colchicine was used as positive control |

**Fig. S1** **a** TLC (silica gel 60, toluene / ethyl formate / formic acid (10:5:3, (v/v)) overview of ethylacetate extract (5 mg/mL) of *C. mapuveronicae* and isolated compounds (**1-10**, 0.5 mg/mL), 366 nm wet; **b** 366 nm dry; **c** daylight; **d** Bornträger reaction induced through ammonia vapor.


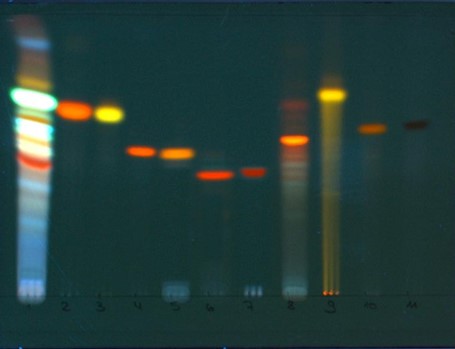

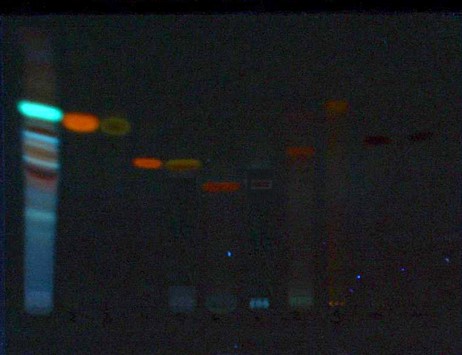


1 2 3 4 5 6 7 8 9 10 11

1 2 3 4 5 6 7 8 9 10 11

**b**

**a**


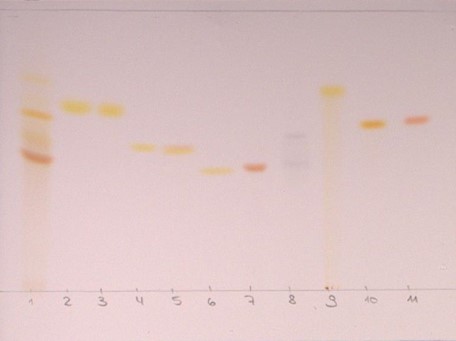

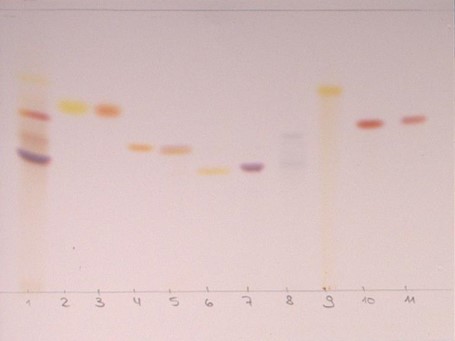


1 2 3 4 5 6 7 8 9 10 11

1 2 3 4 5 6 7 8 9 10 11

**d**

**c**

1 Ethylacetate extract of *C. mapuveronicae*

2 Emodin-6,8-di-*O*-methylether (**4**)

3 Emodin (**3**)

4 Questin (**5**)

5 Endocrocin (**10**)

6 Dermolutein (**9**)

7 Clavorubin-8-*O*-methylether (**1**)

8 Hypericin (**8**)

9 (+)-7,7’-Emodinphyscion (**2**)

10 (+)-(*S*)-Skyrin (**6**)

11 (+)-(*S*)-Aurantioskyrin (**7**)

**
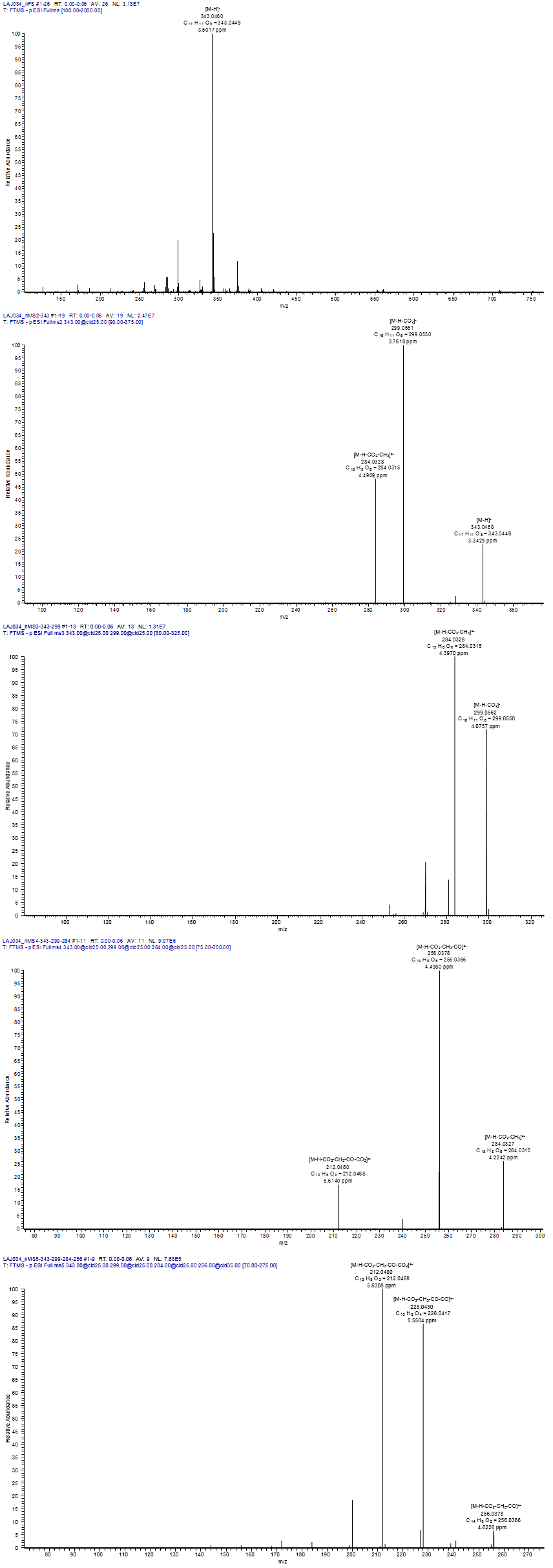
Fig. S2** Negative ion HRESIMS^n^ spectra of clavorubin-8-*O*-methylether (**1**).

**
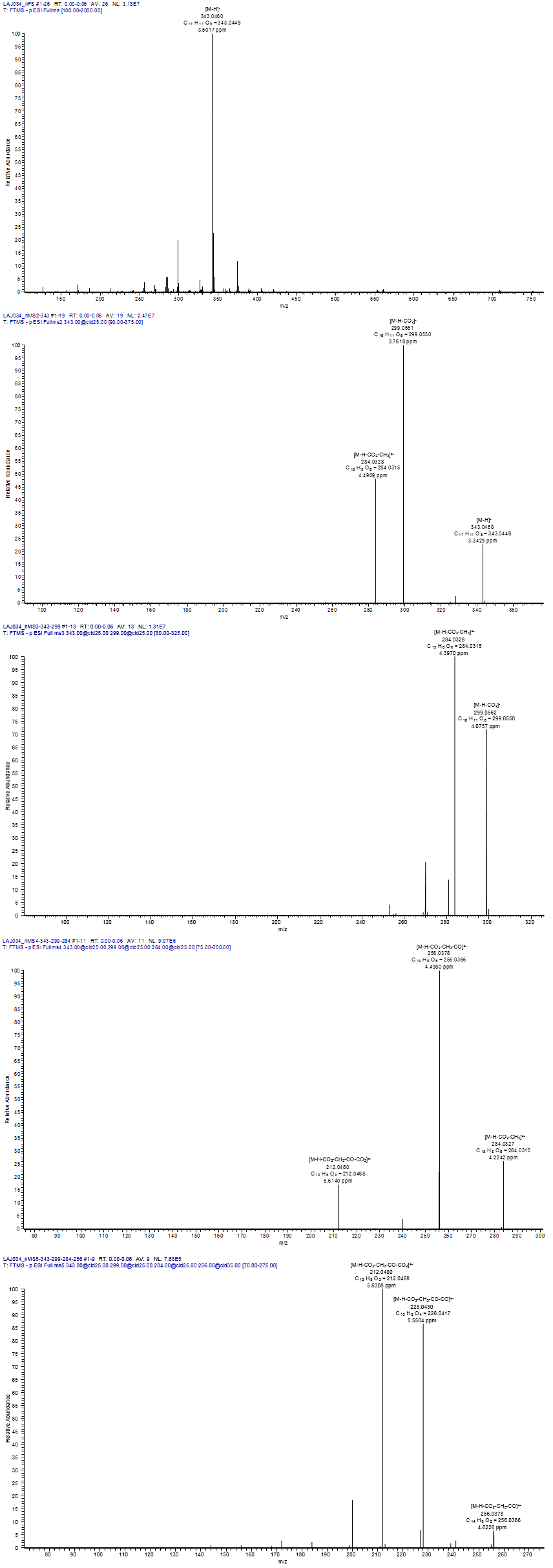
**

**Fig. S3** ^1^H NMR spectrum (500 MHz, THF-*d*_8_) of clavorubin-8-*O*-methylether (**1**).

**
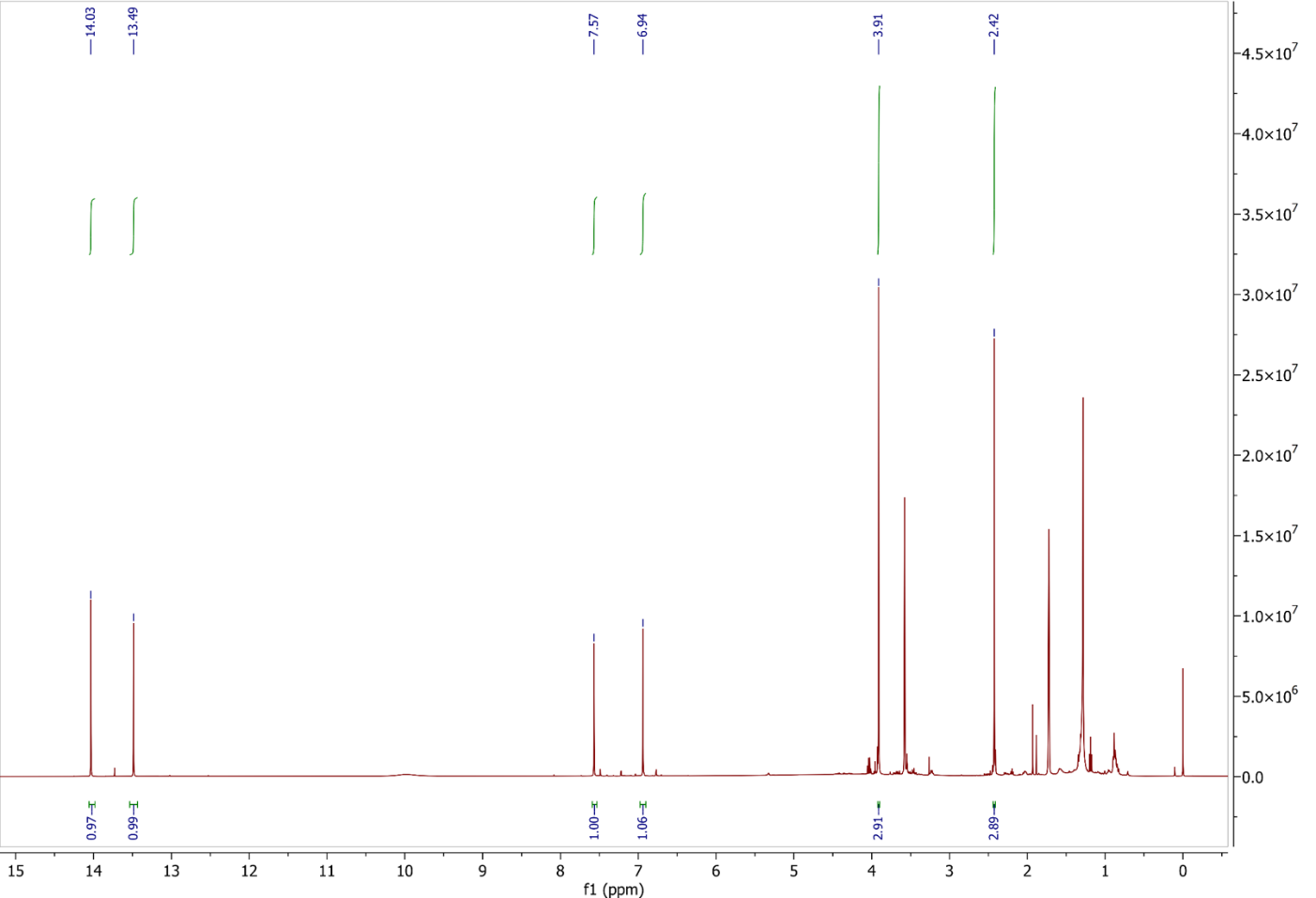
**

**
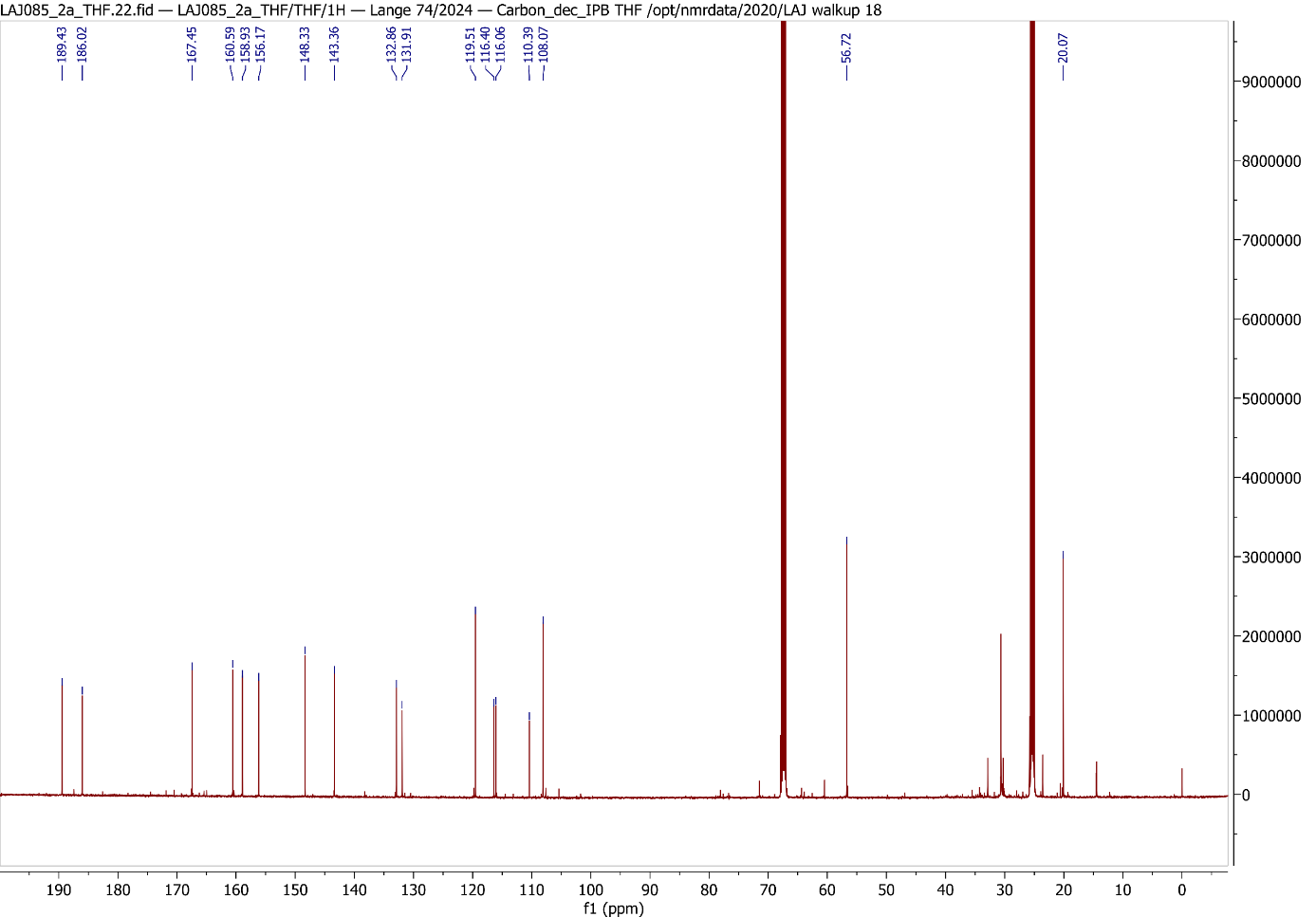
Fig. S4** ^13^C NMR spectrum (125 MHz, THF-*d*_8_) of clavorubin-8-*O*-methylether (**1**).

**Fig. S5** HSQC spectrum (500/125 MHz, THF-*d*_8_) of clavorubin-8-*O*-methylether (**1**).


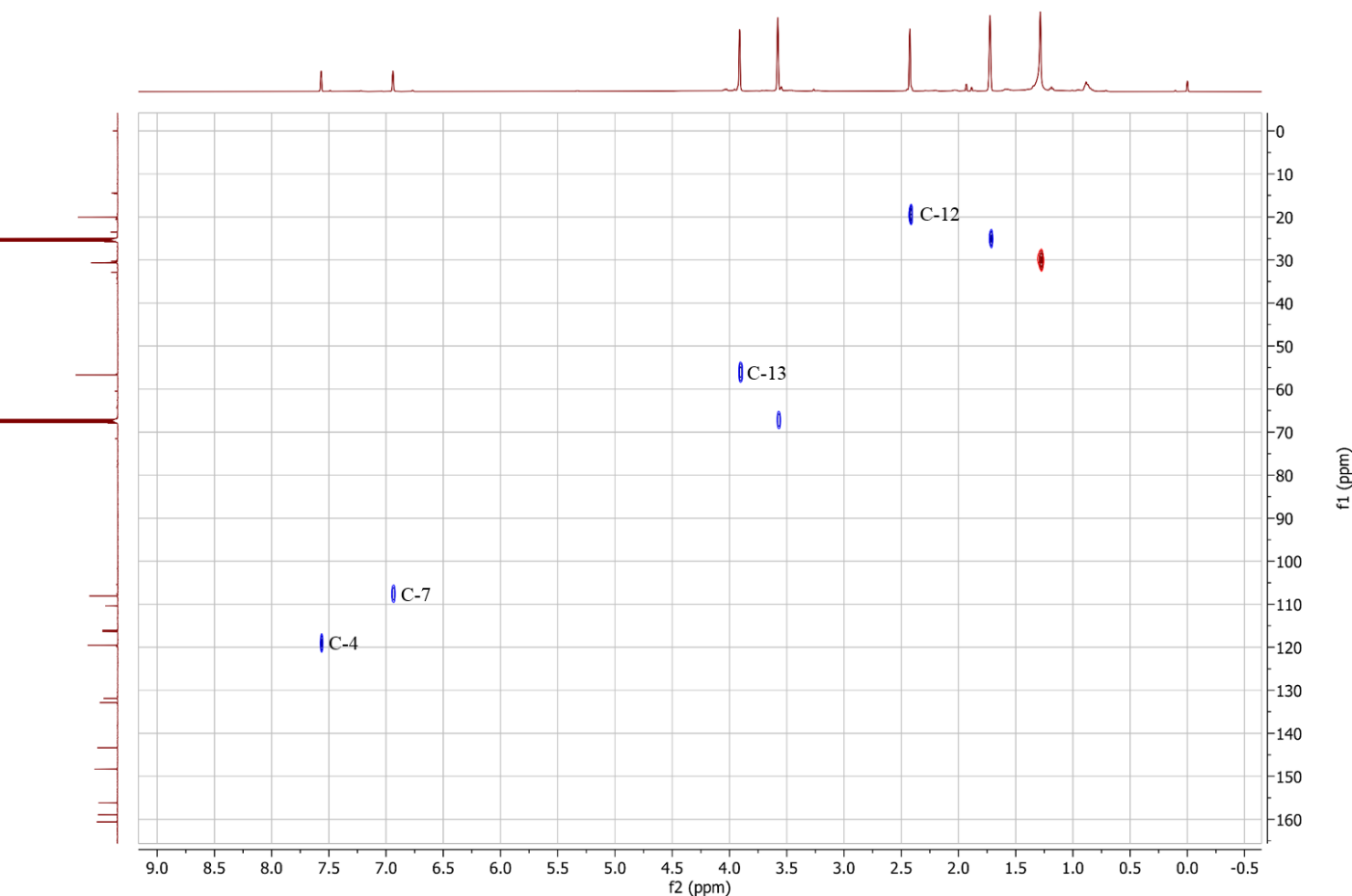


**Fig. S6** HMBC spectrum (500/125 MHz, THF-*d*_8_) of clavorubin-8-*O*-methylether (**1**).

**
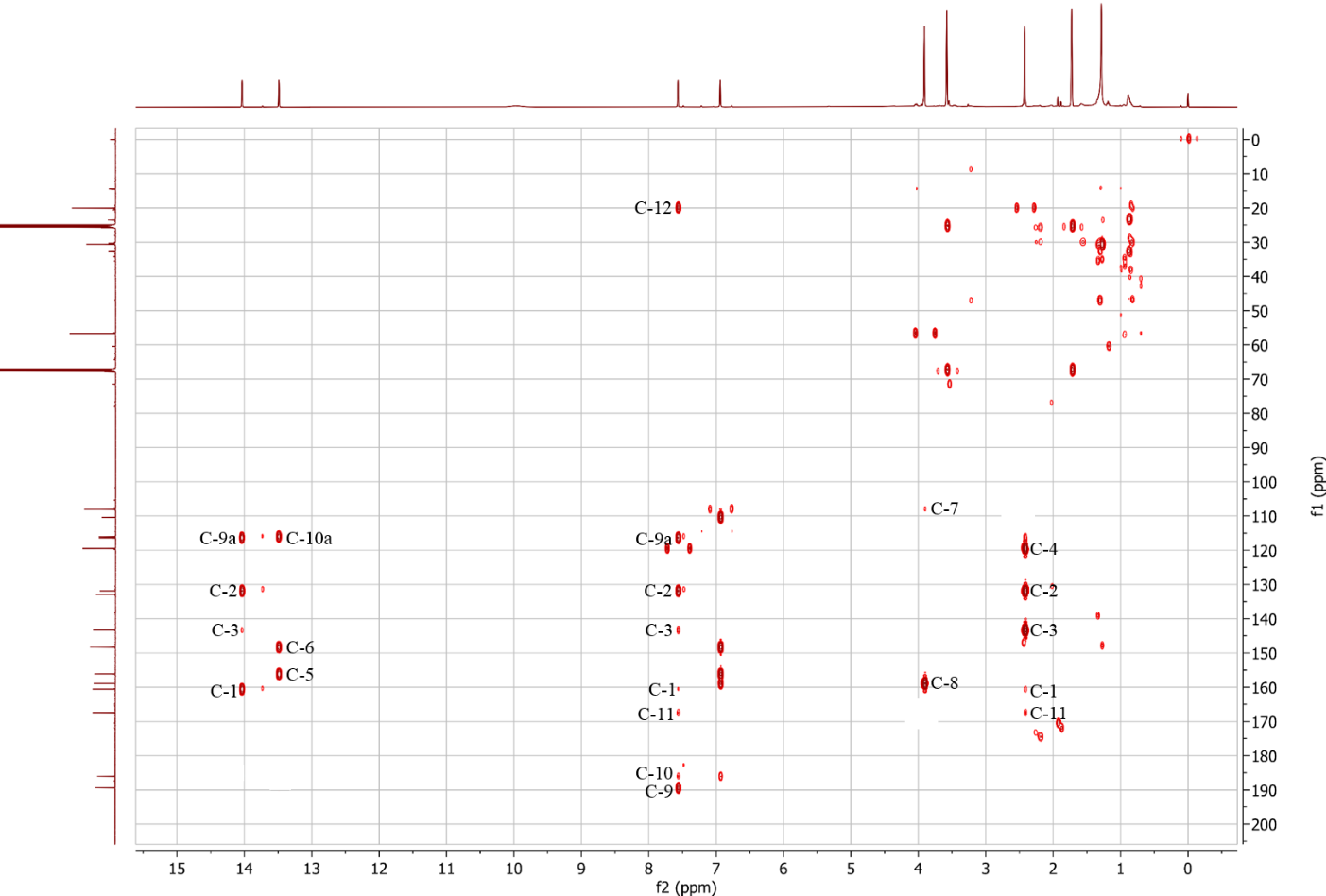
**

**Fig. S7** NOESY spectrum (500/125 MHz, THF-*d*_8_) of clavorubin-8-*O*-methylether (**1**).

**
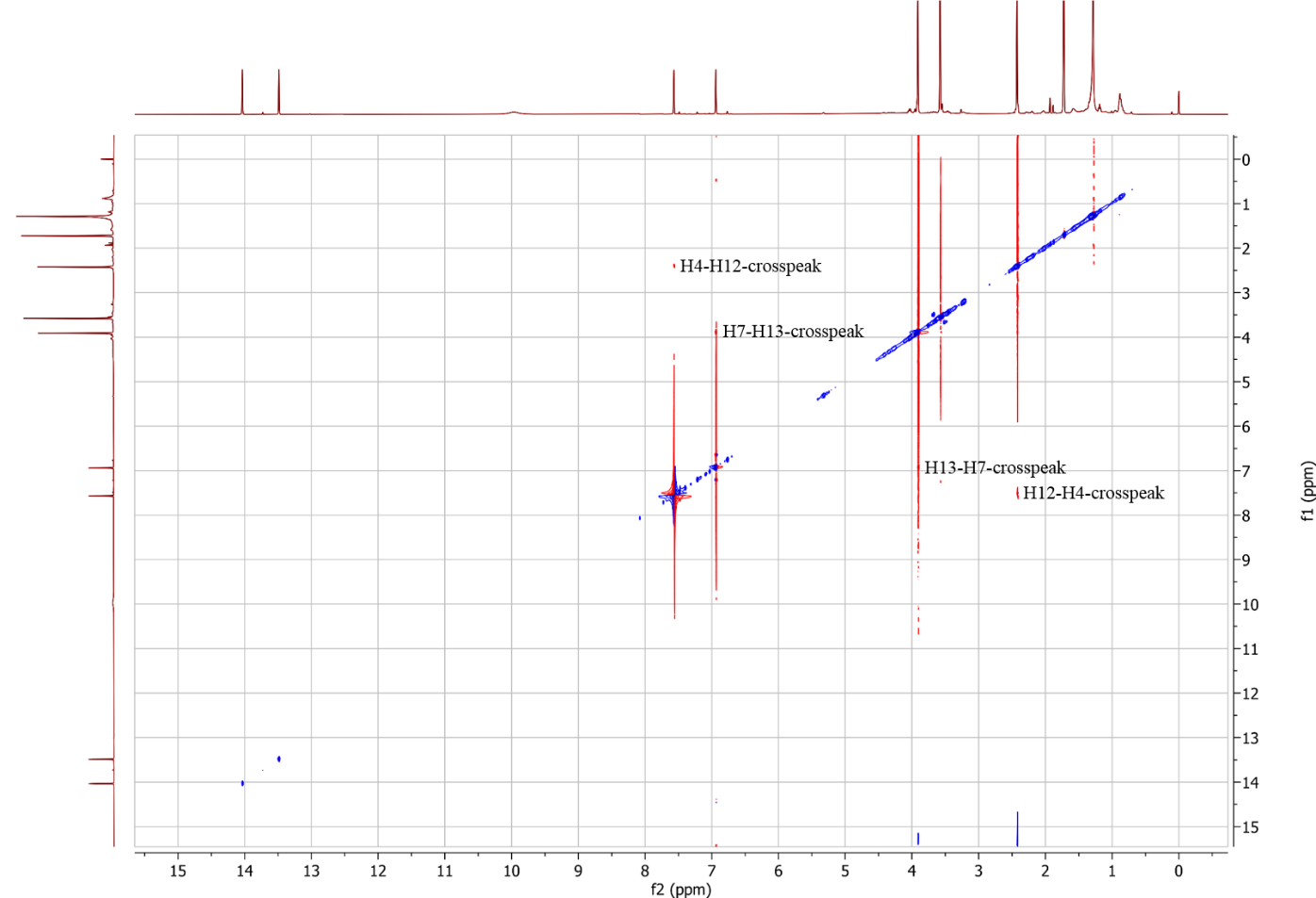
**

**Fig. S8** UV spectrum of clavorubin-8-*O*-methylether (**1**).

**
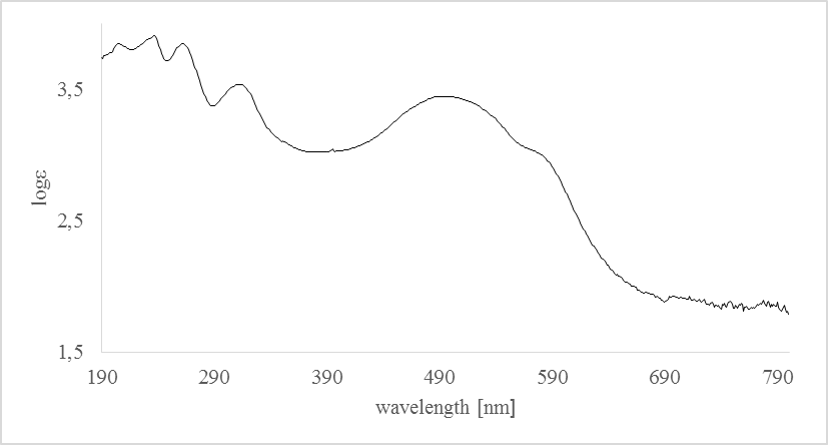
**

**Fig. S9** Negative ion HRESIMS^n^ spectra of (+)-7,7’-emodinphyscion (**2**).
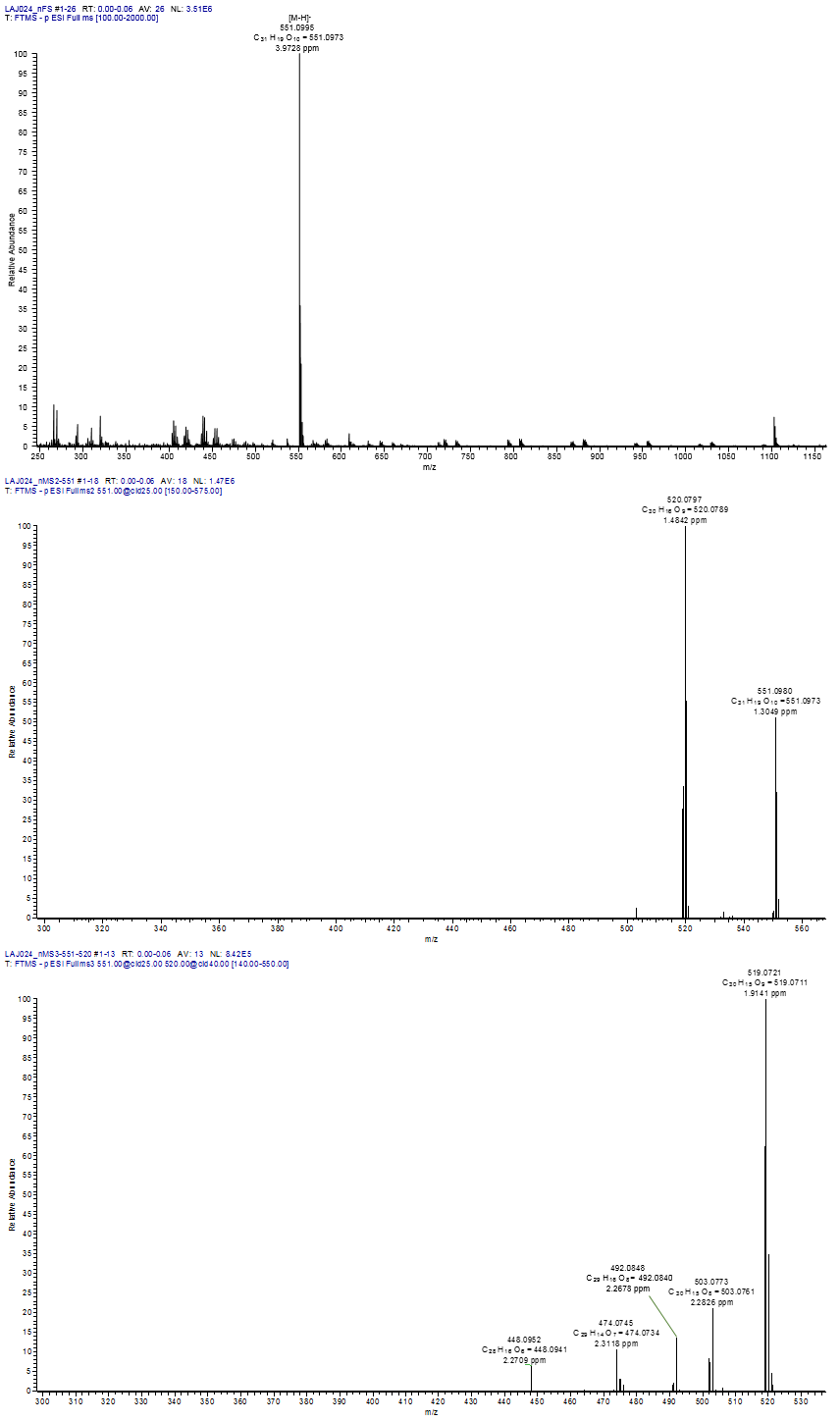


**Fig. S10** ^1^H NMR spectrum (500 MHz, pyridine-*d*_5_) of (+)-7,7’-emodinphyscion (**2**).

**
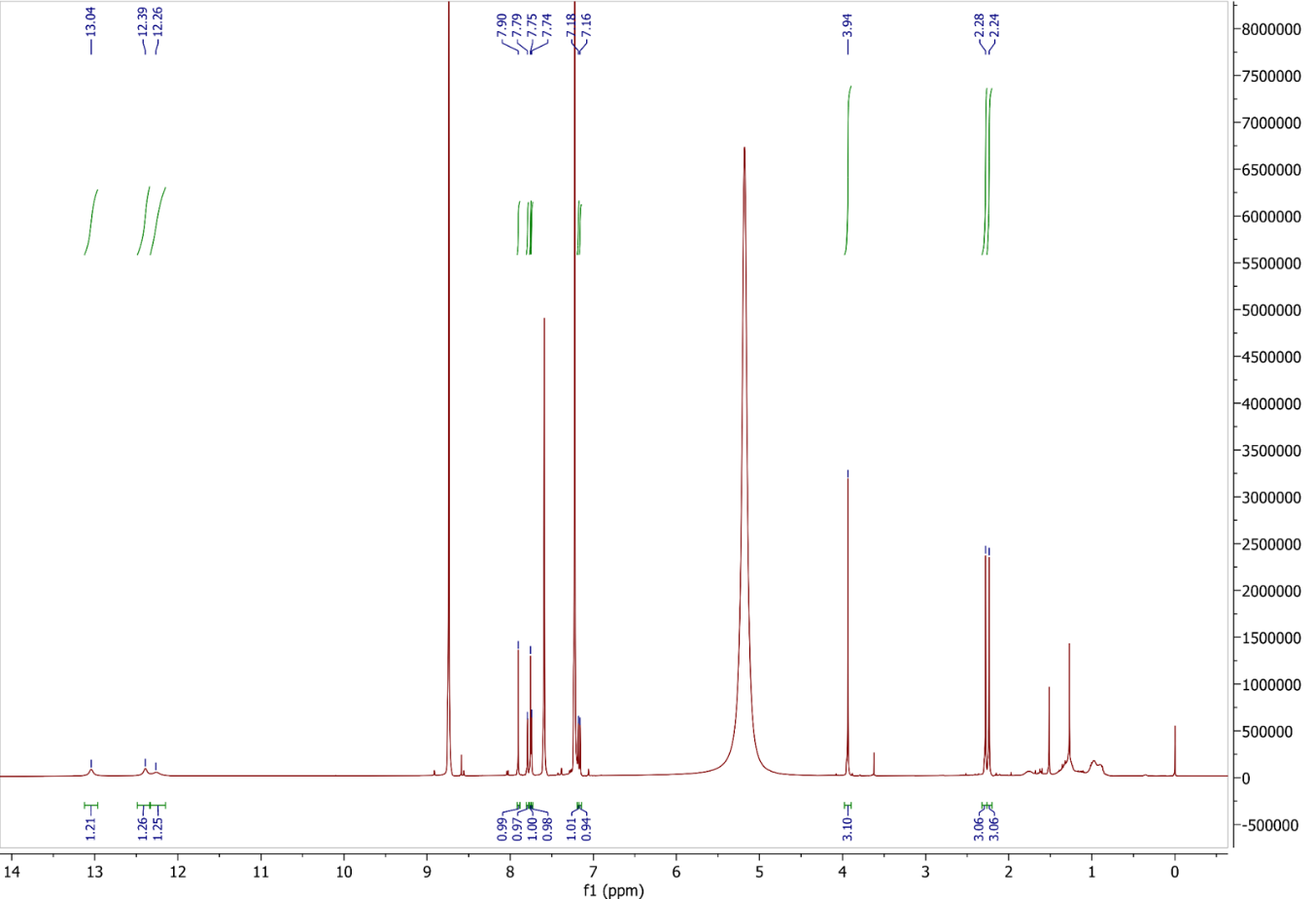
**

**Fig. S11** ^13^C NMR spectrum (125 MHz, pyridine-*d*_5_) of (+)-7,7’-emodinphyscion (**2**).

**
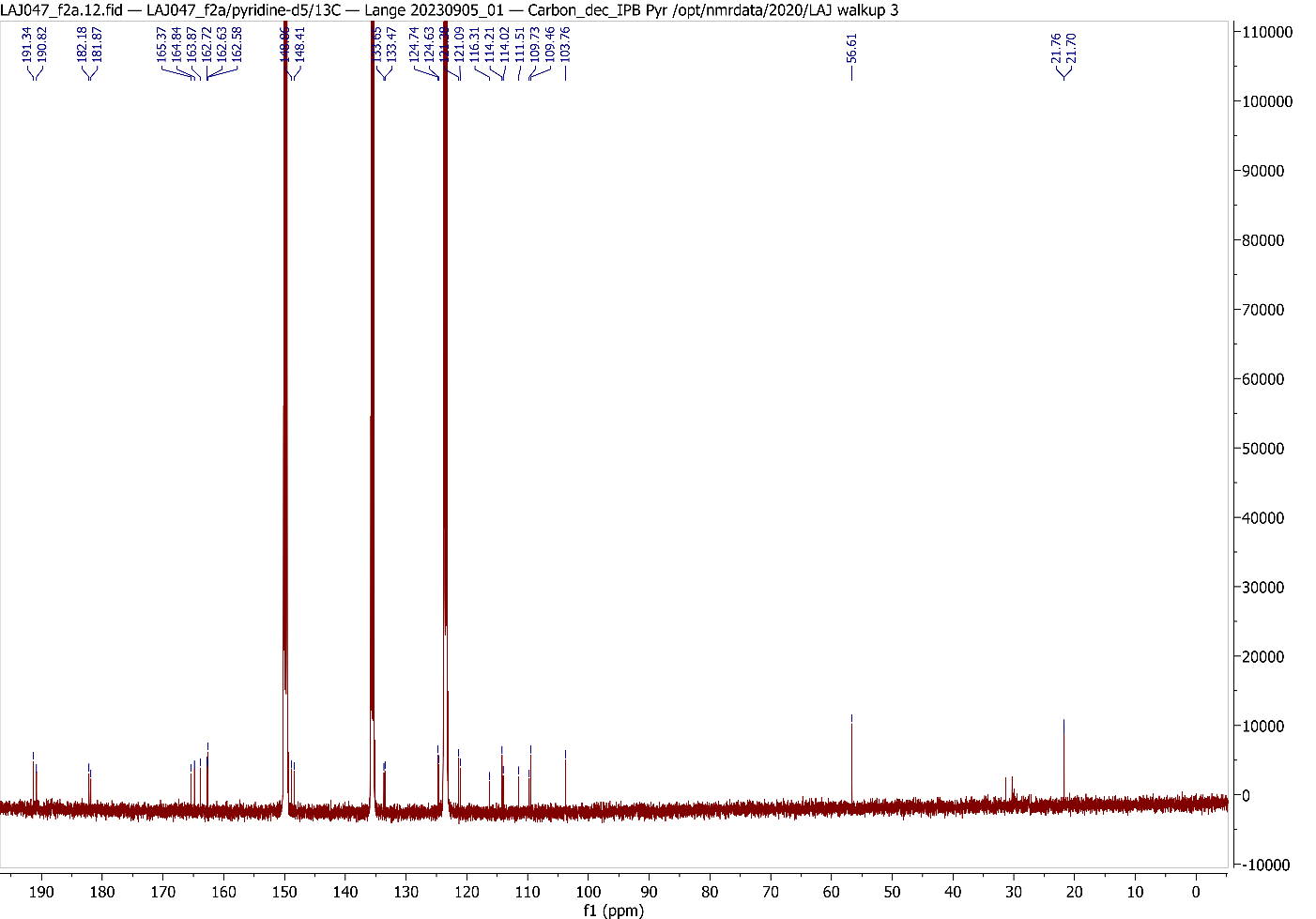
**

**Fig. S12** CD spectrum (MeOH) of (+)-7,7’-emodinphyscion (**2**).


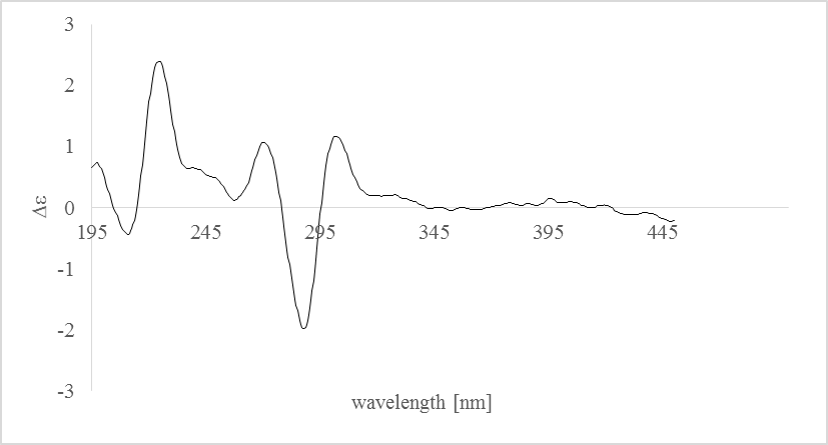


**Fig. S13** Negative ion HRESIMS^n^ spectra of emodin (**3**).

**
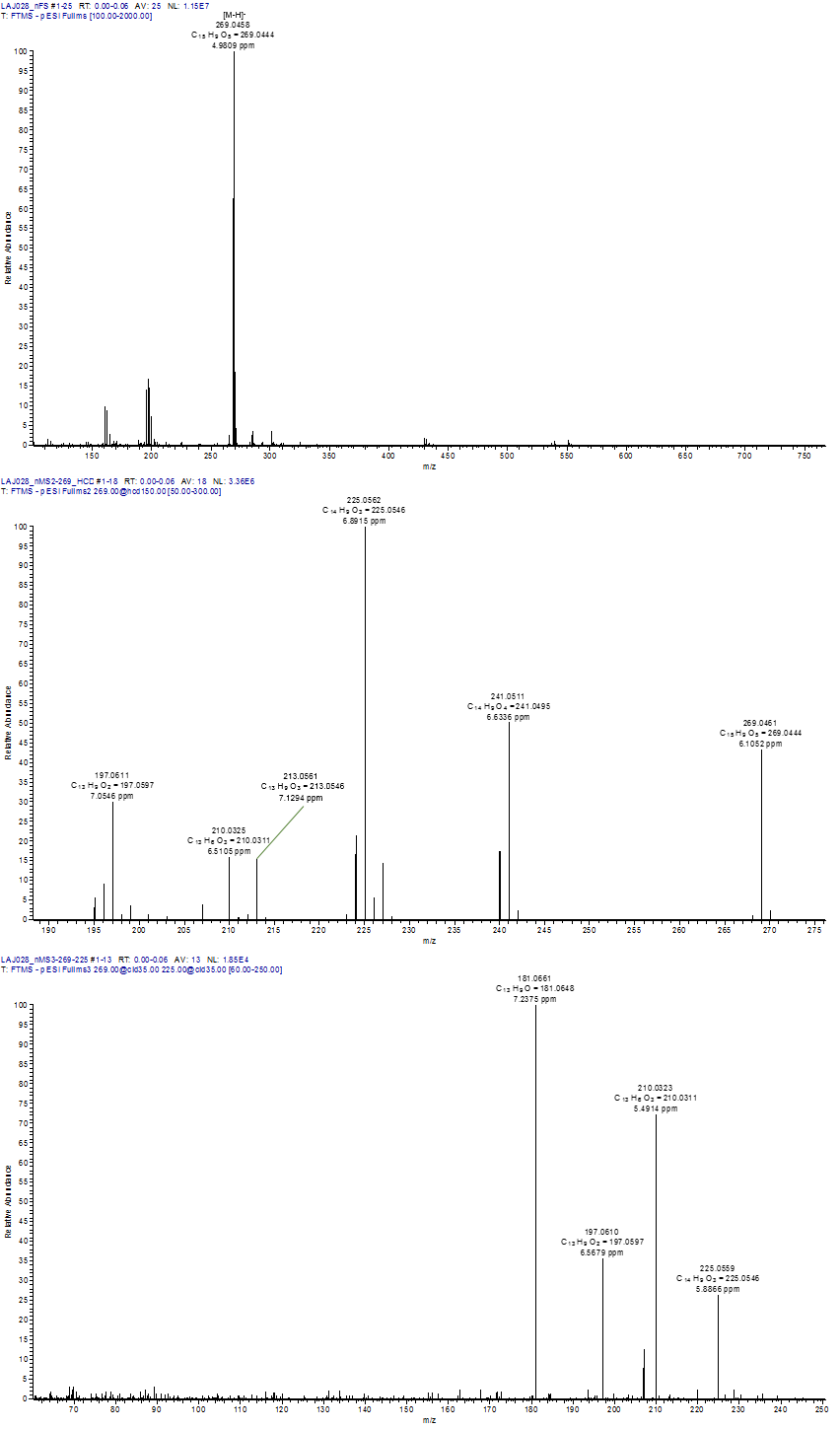
**

**Fig. S14** ^1^H NMR spectrum (500 MHz, CDCl_3_) of emodin (**3**).


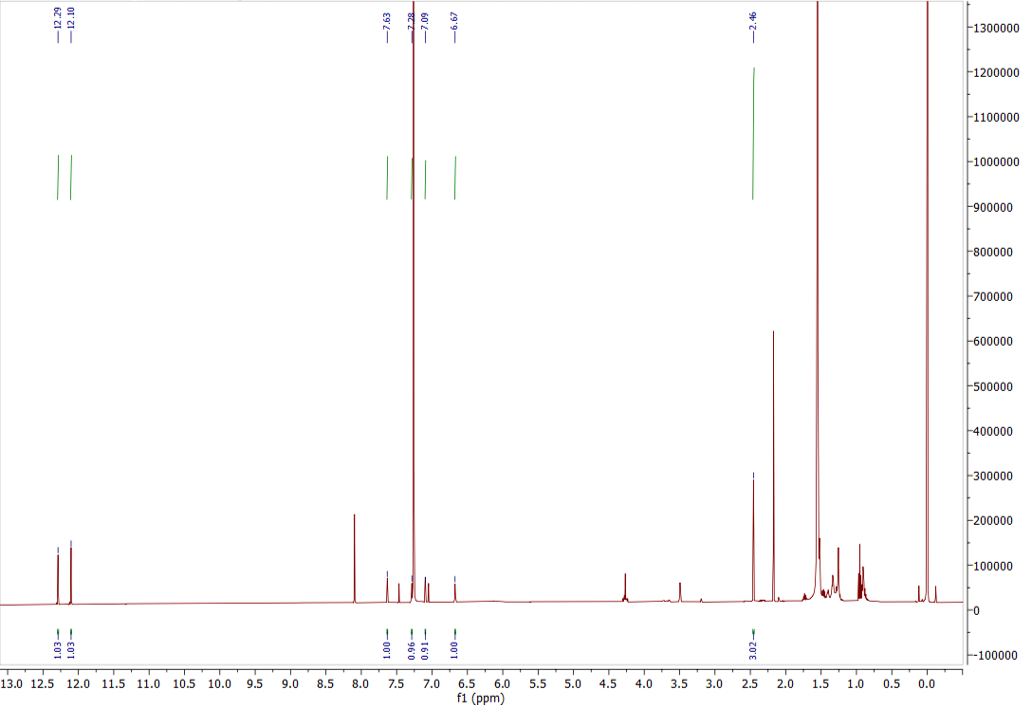


**Fig. S15** HSQC spectrum (500/125 MHz, CDCl_3_) of emodin (**3**).

**
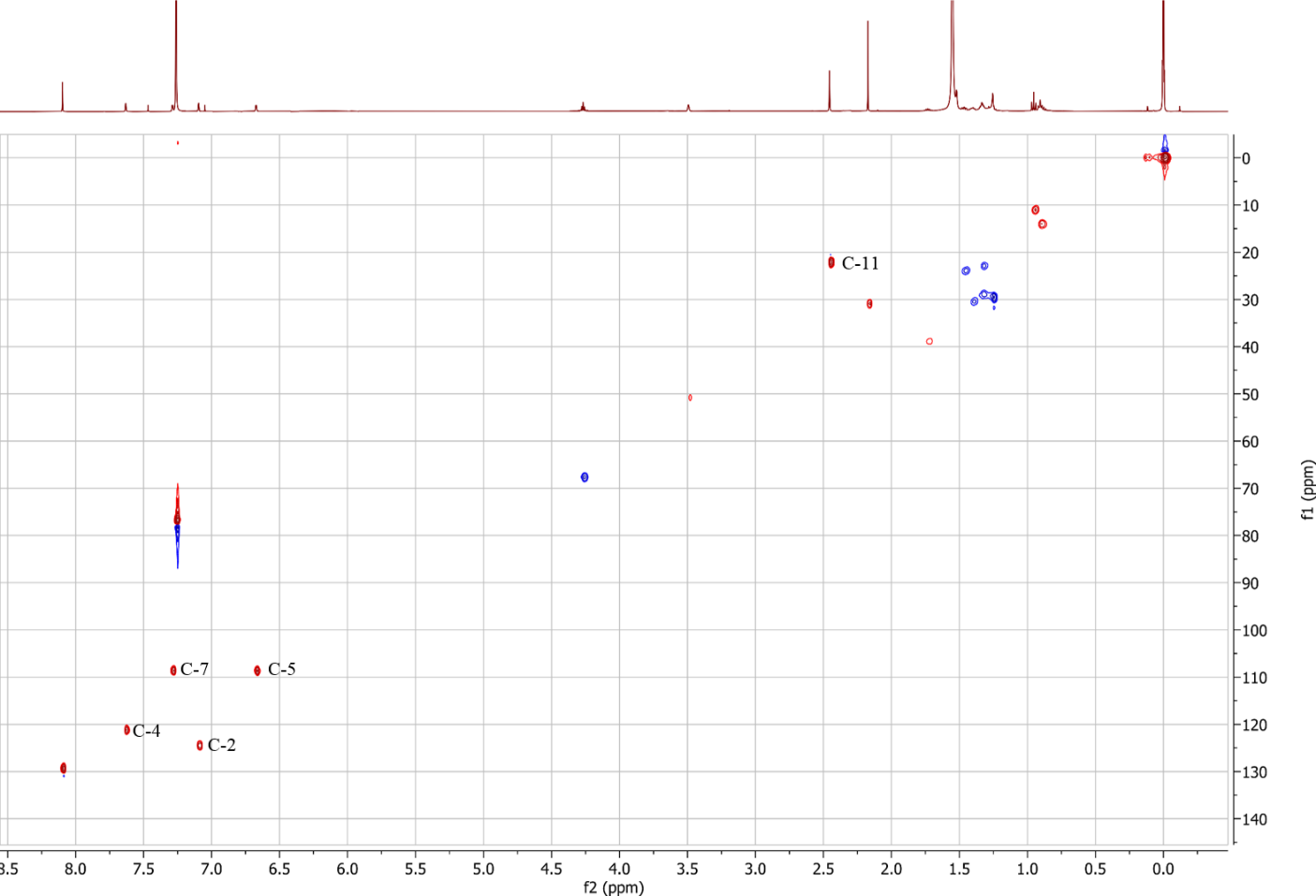
**

**
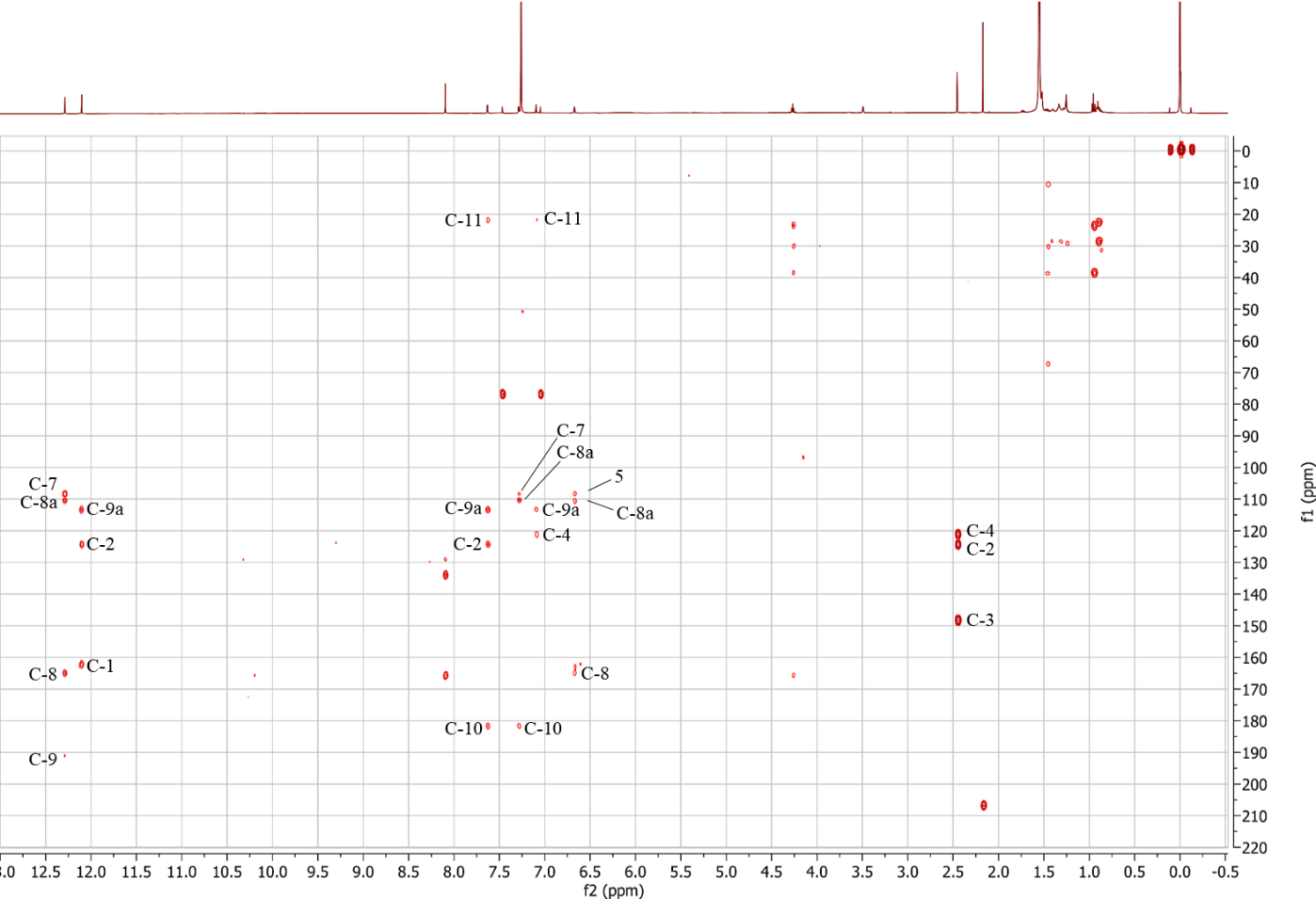
Fig. S16** HMBC spectrum (500/125 MHz, CDCl_3_) of emodin (**3**).

**Fig. S17** Negative ion HRESIMS^n^ spectra of emodin-6,8-di-*O*-methylether (**4**).

**
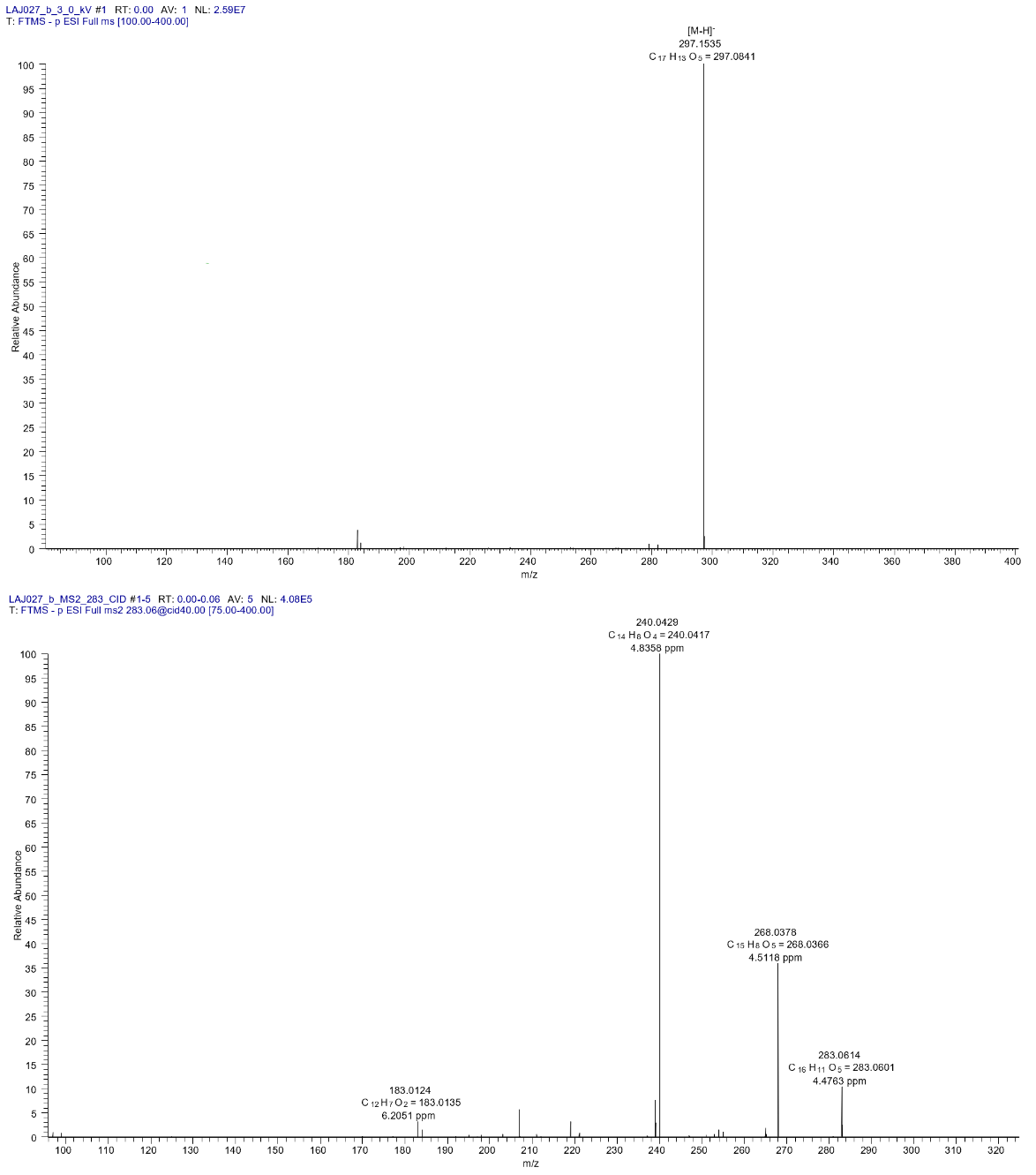
**

**Fig. S18** ^1^H NMR spectrum (500 MHz, CDCl_3_) of emodin-6,8-di-*O*-methylether (**4**).

**
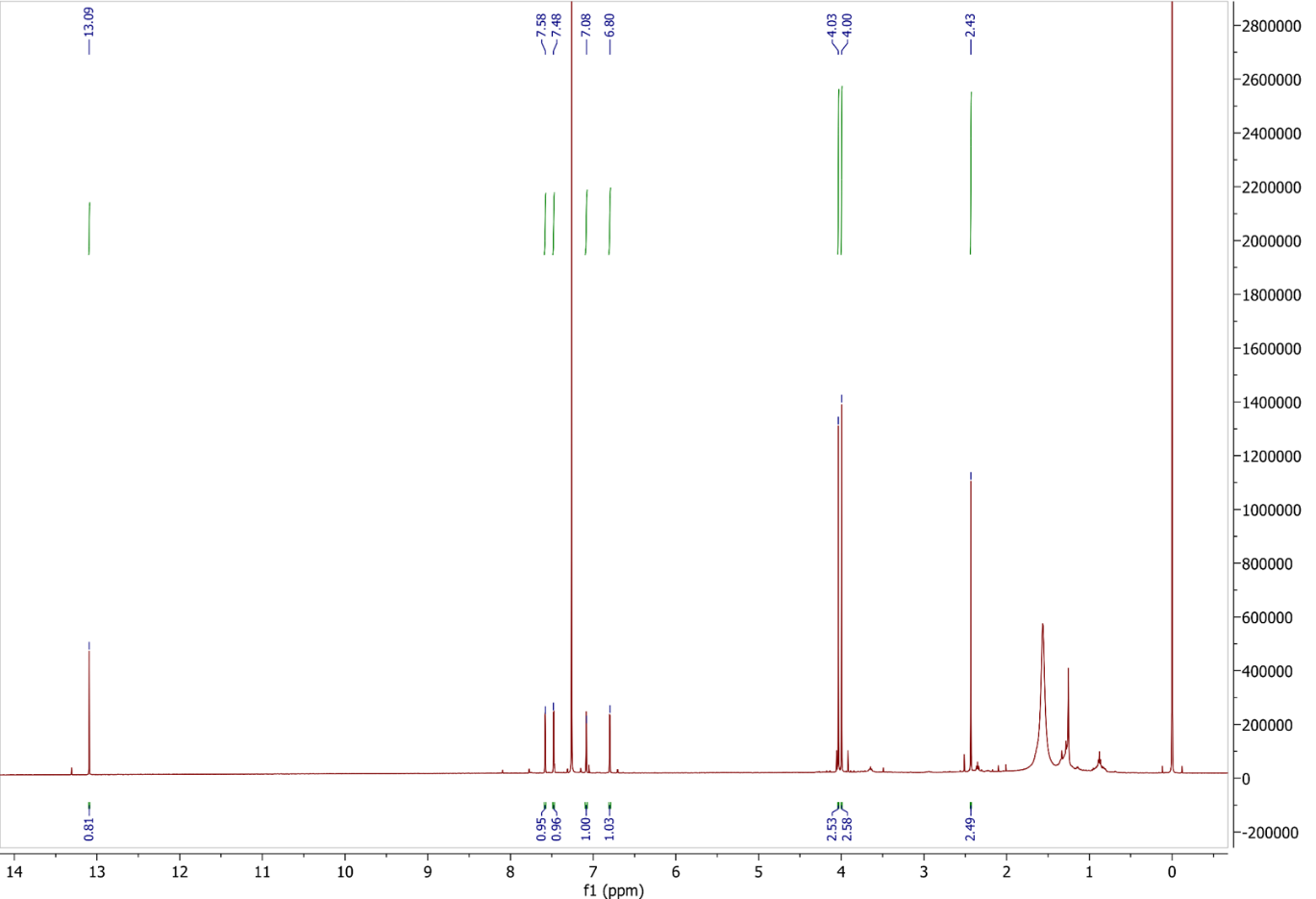
**

**Fig. S19** HSQC spectrum (500/125 MHz, CDCl_3_) of emodin-6,8-di-*O*-methylether (**4**).


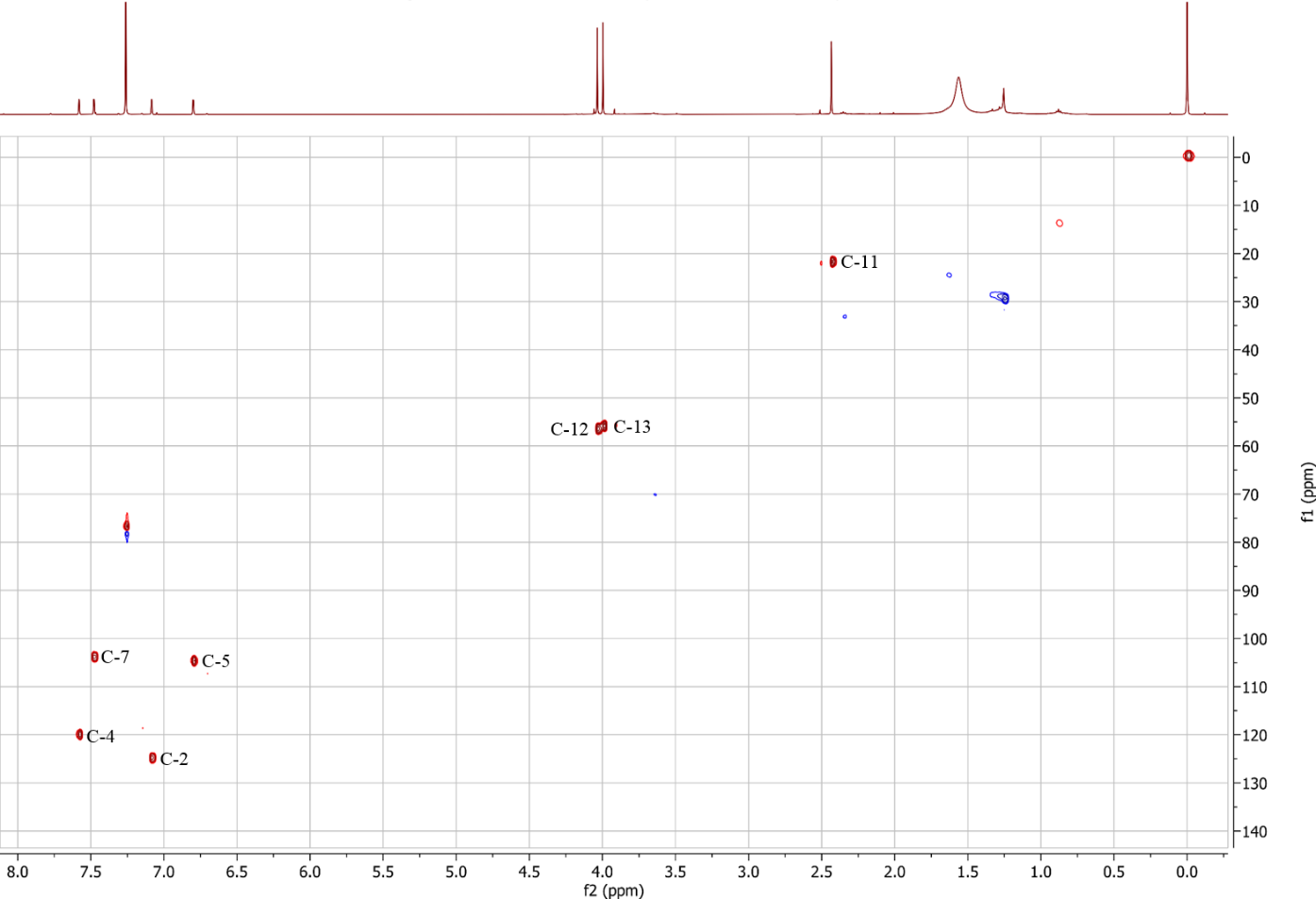


**Fig. S20** HMBC spectrum (500/125 MHz, CDCl_3_) of emodin-6,8-di-*O*-methylether (**4**).

**
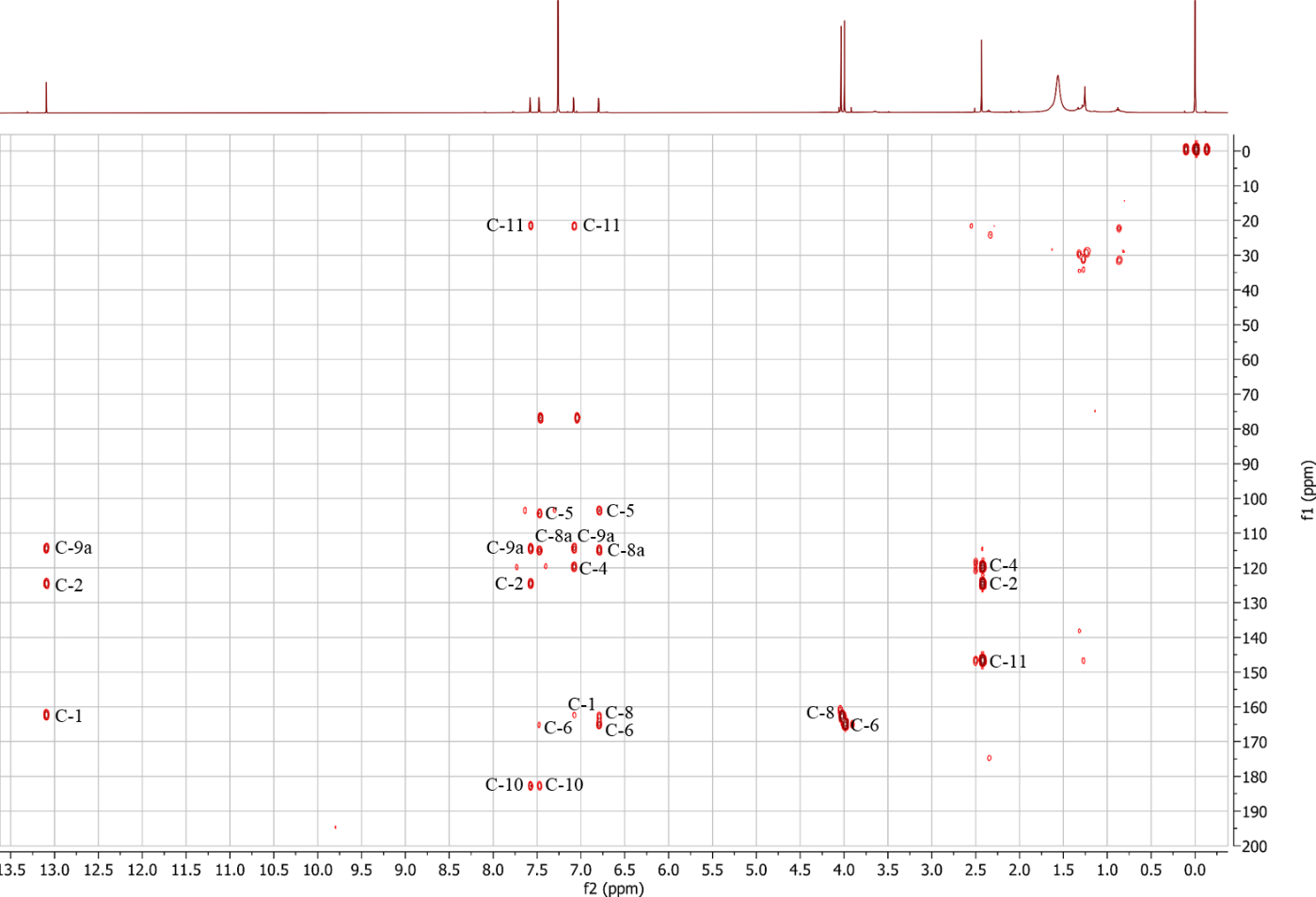
**

**Fig. S21** Negative ion HRESIMS^n^ spectra of questin (**5**).

**
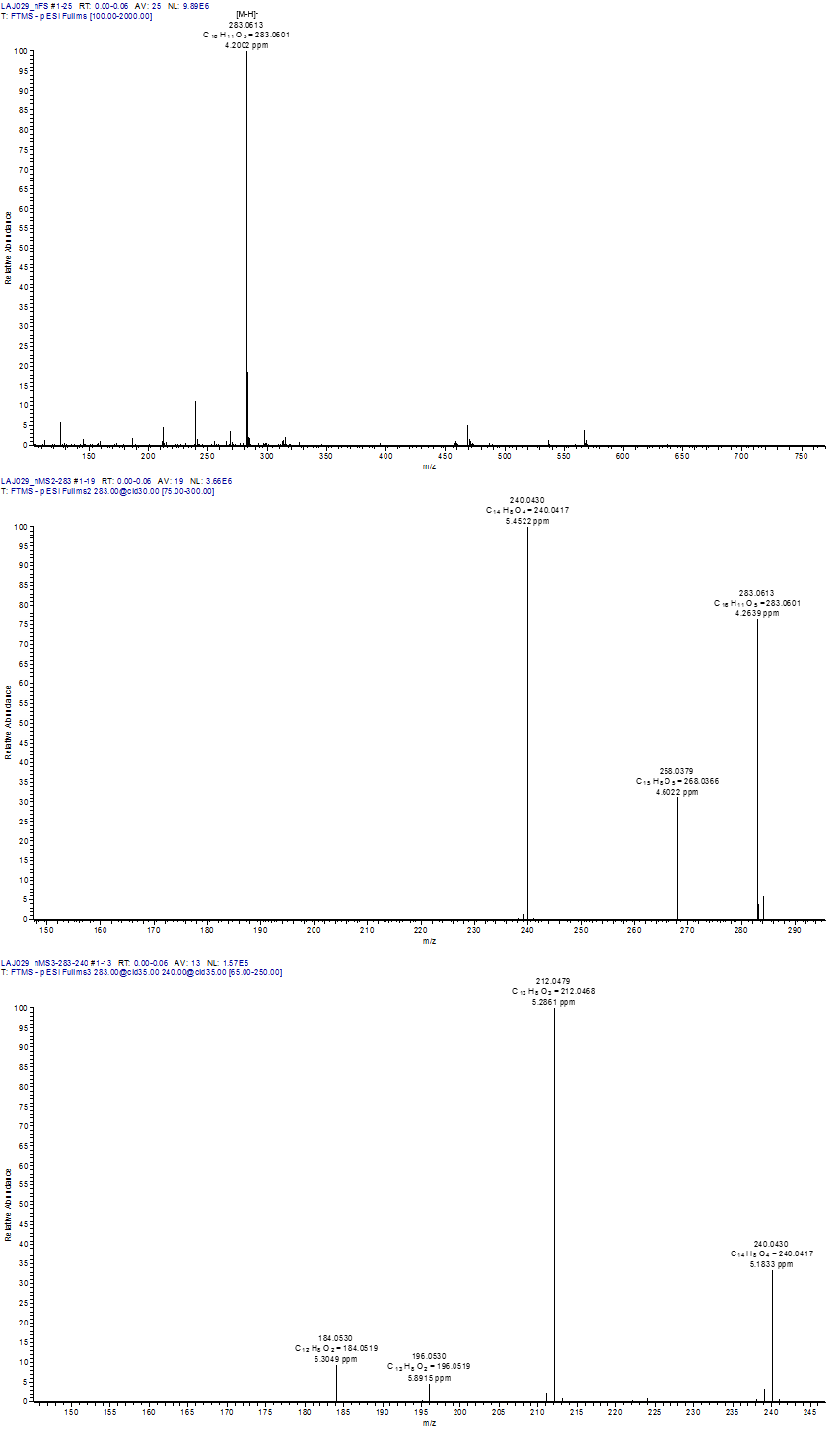
**

**Fig. S22** ^1^H NMR spectrum (500 MHz, CDCl_3_) of questin (**5**).


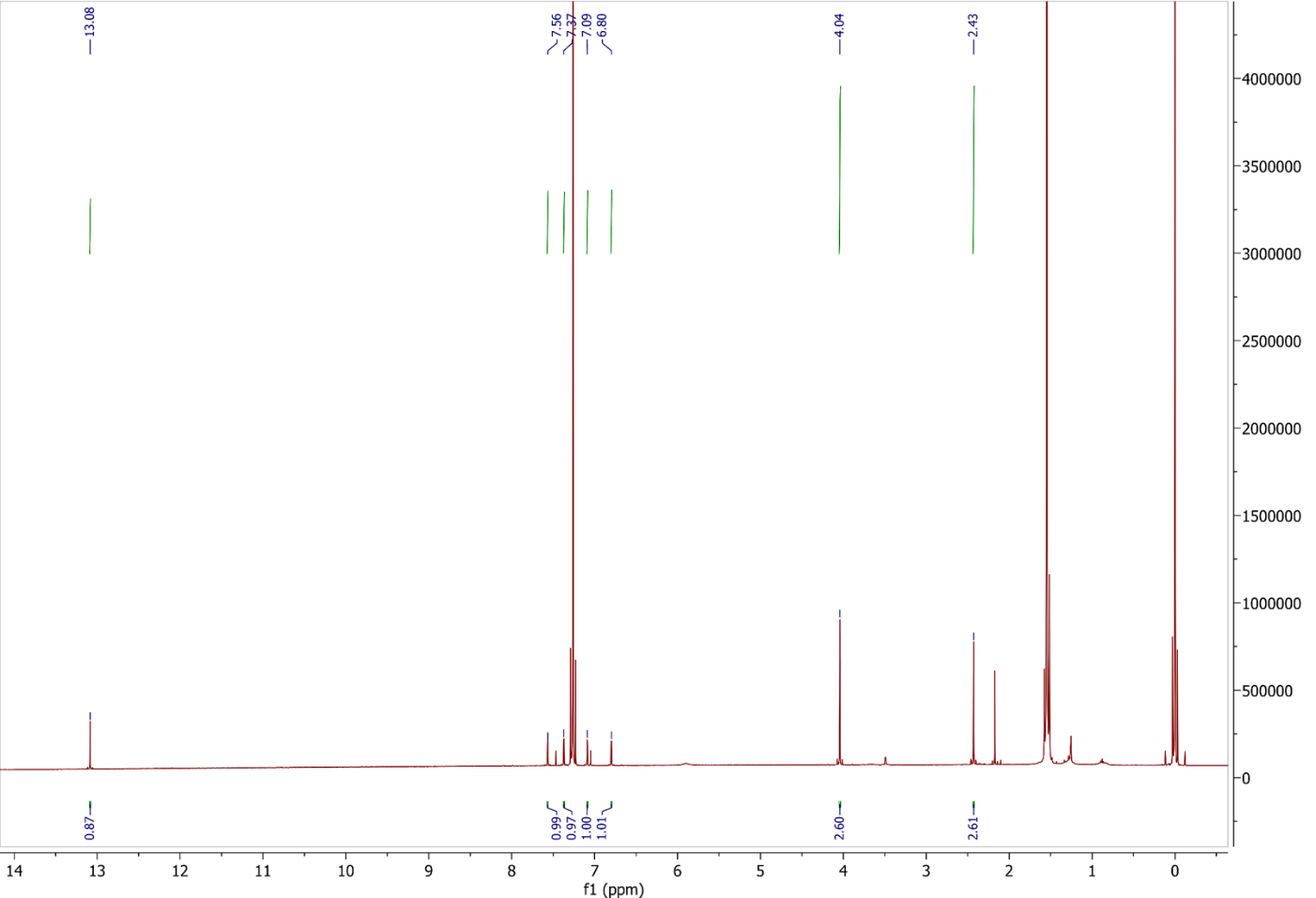


**Fig. S23** HSQC spectrum (500/125 MHz, CDCl_3_) of questin (**5**).


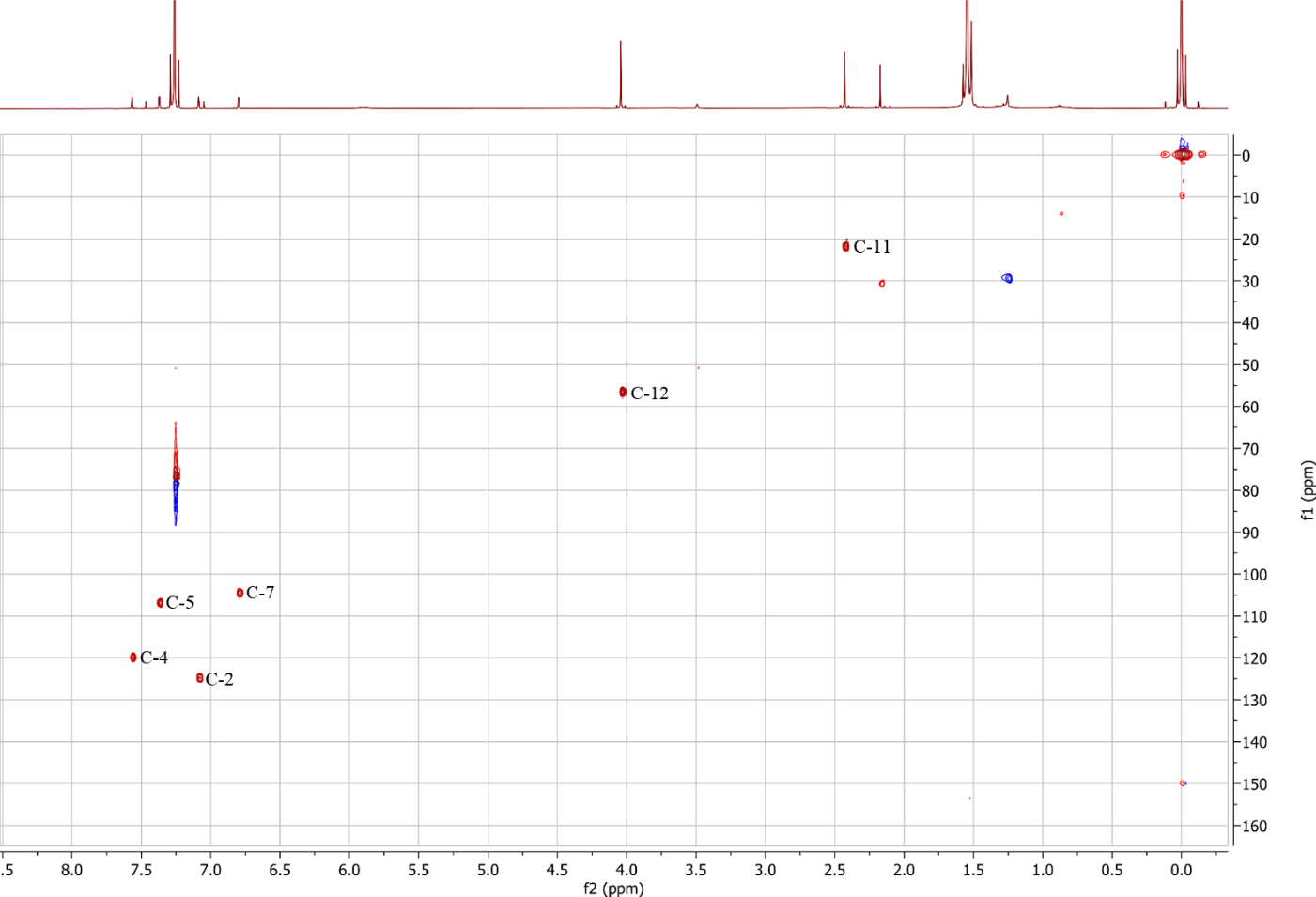


**Fig. S24** HMBC spectrum (500/125 MHz, CDCl_3_) of questin (**5**).

**
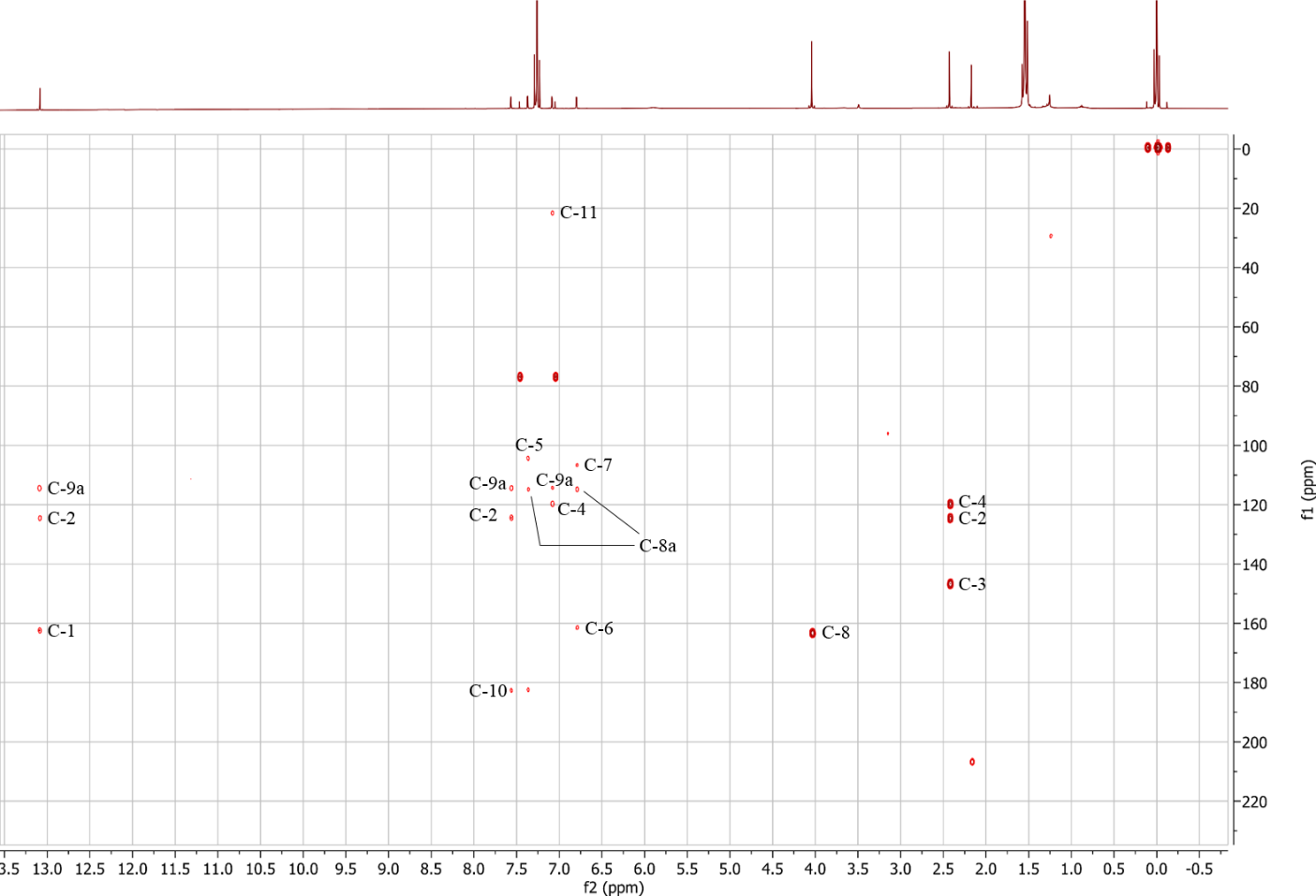
**

**Fig. S25** Negative ion HRESIMS^n^ spectra of (+)-(*S*)-skyrin (**6**).

**
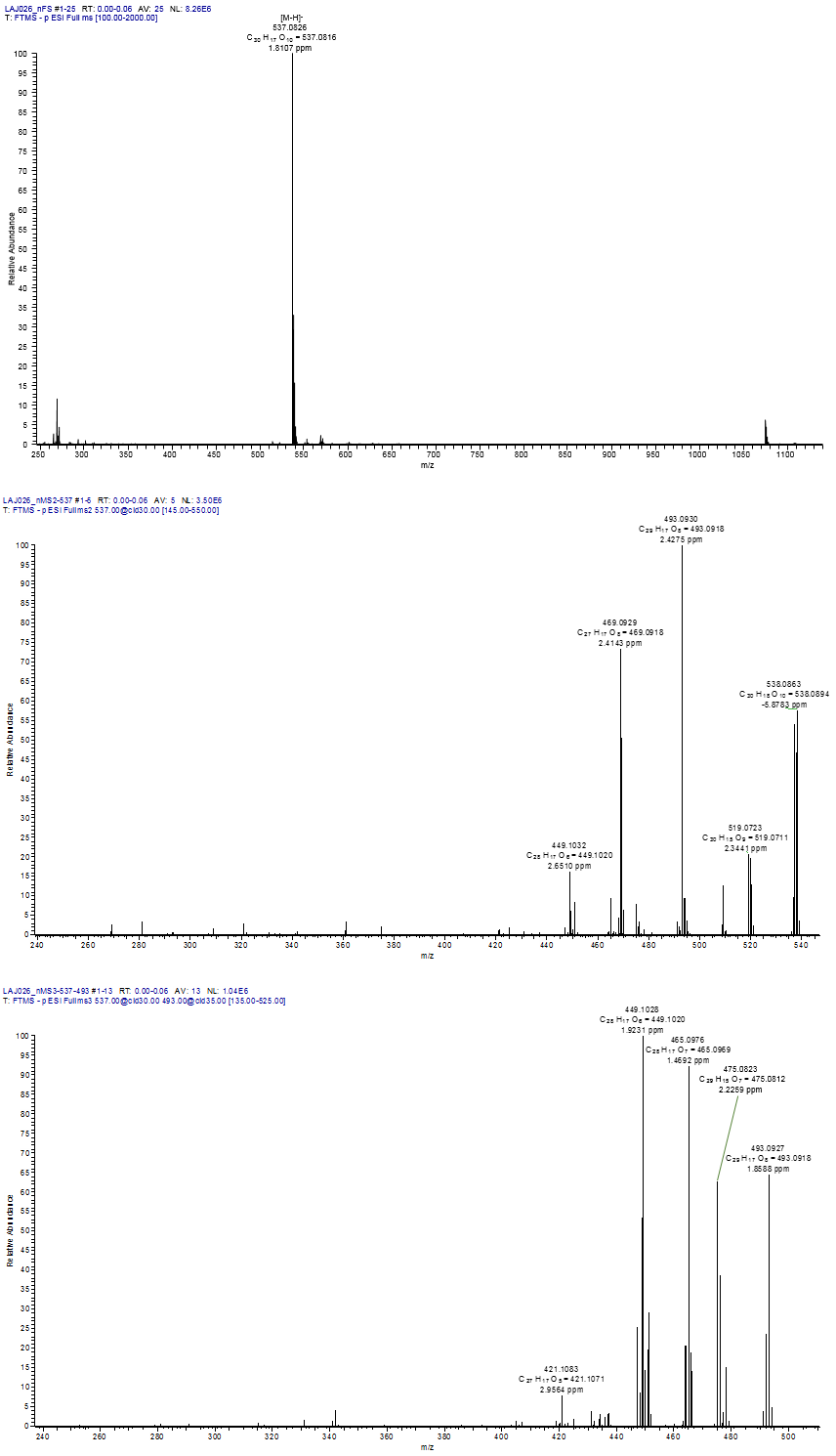
**

**Fig. S26** ^1^H NMR spectrum (500 MHz, DMSO-*d*_6_) of (+)-(*S*)-skyrin (**6**).

**
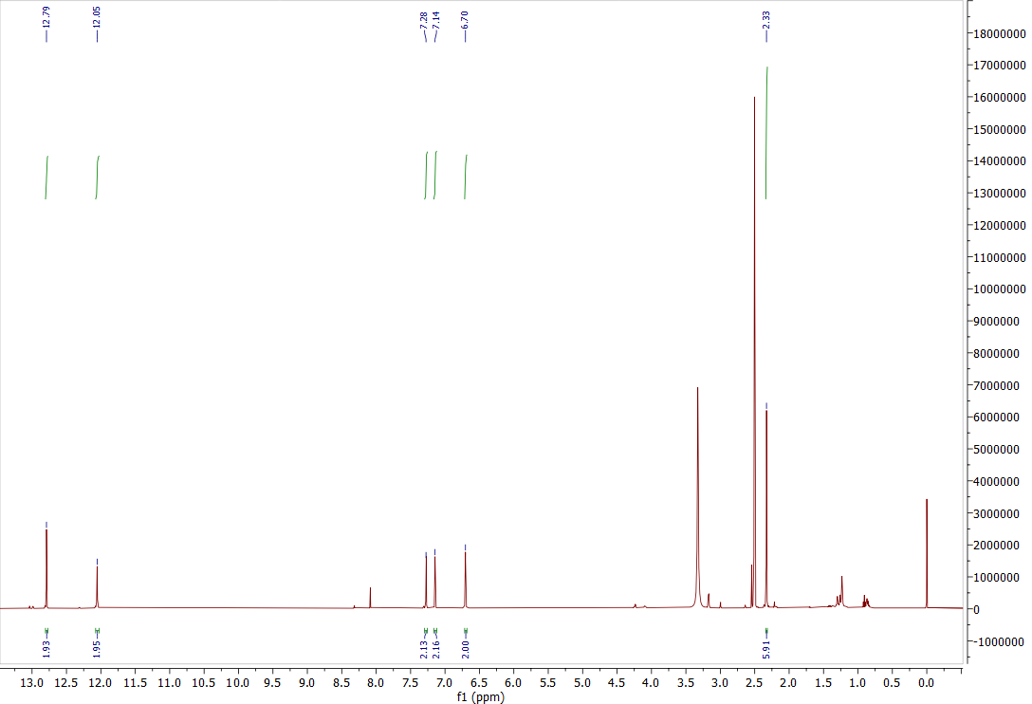
**

**Fig. S27** ^13^C NMR spectrum (500 MHz, DMSO-*d*_6_) of (+)-(*S*)-skyrin (**6**).


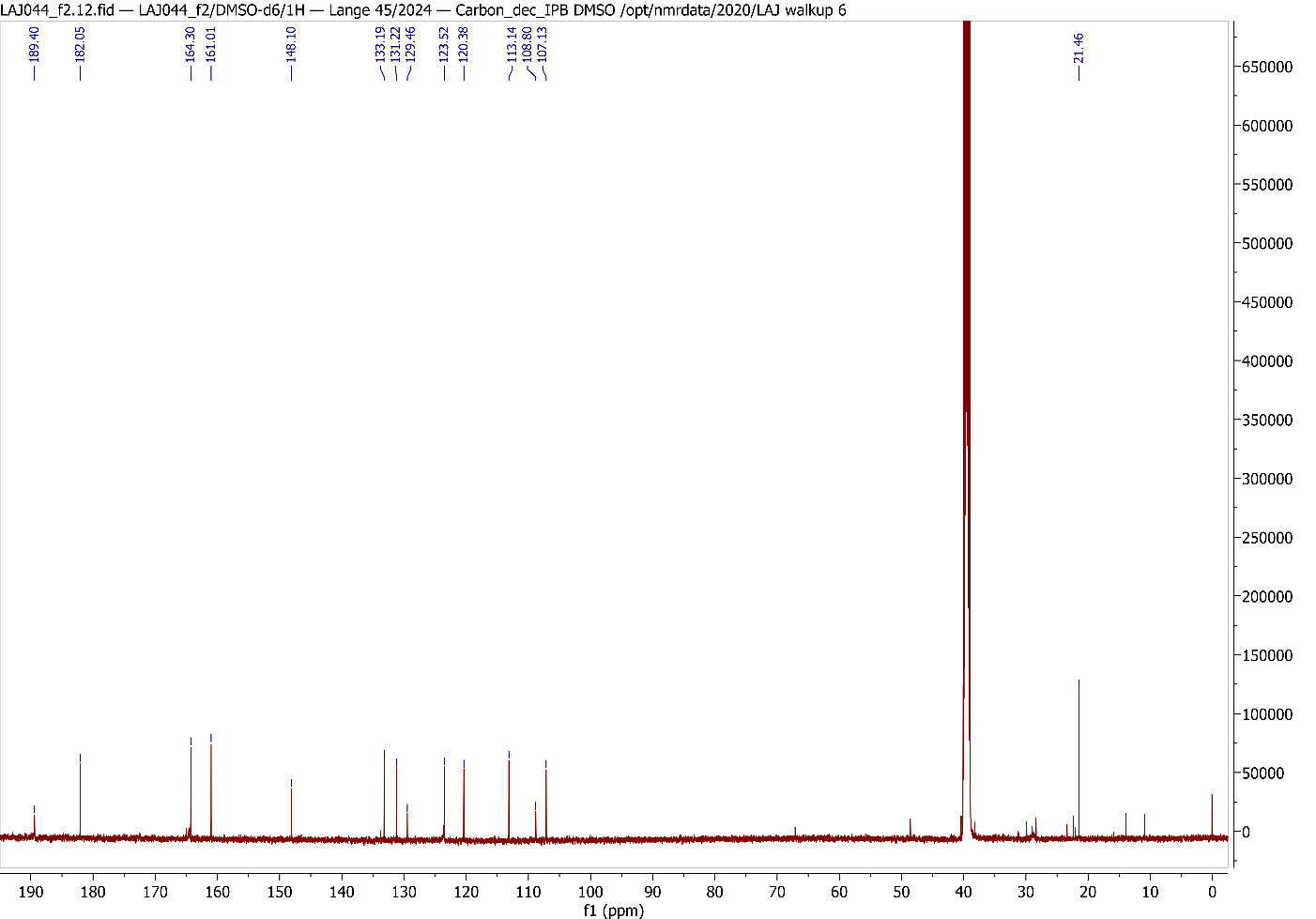


**Fig. S28** CD spectrum (MeOH) of (+)-(*S*)-skyrin (**6**).

**
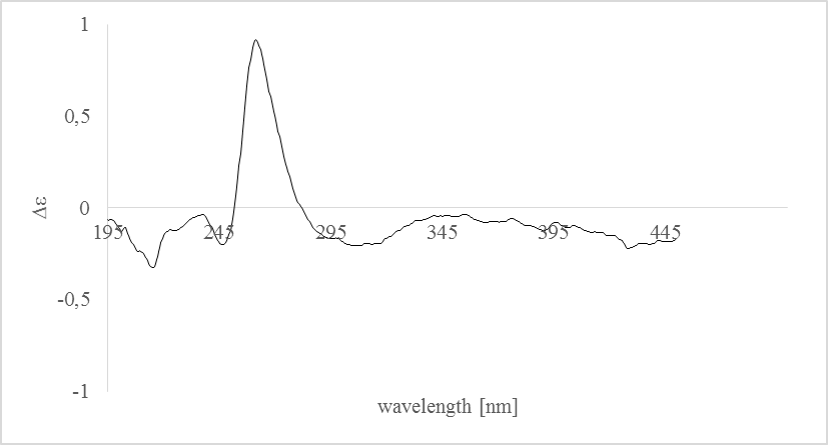
**

**Fig. S29** Negative ion HRESIMS^n^ spectra of (+)-(*S*)-aurantioskyrin (**7**).

**
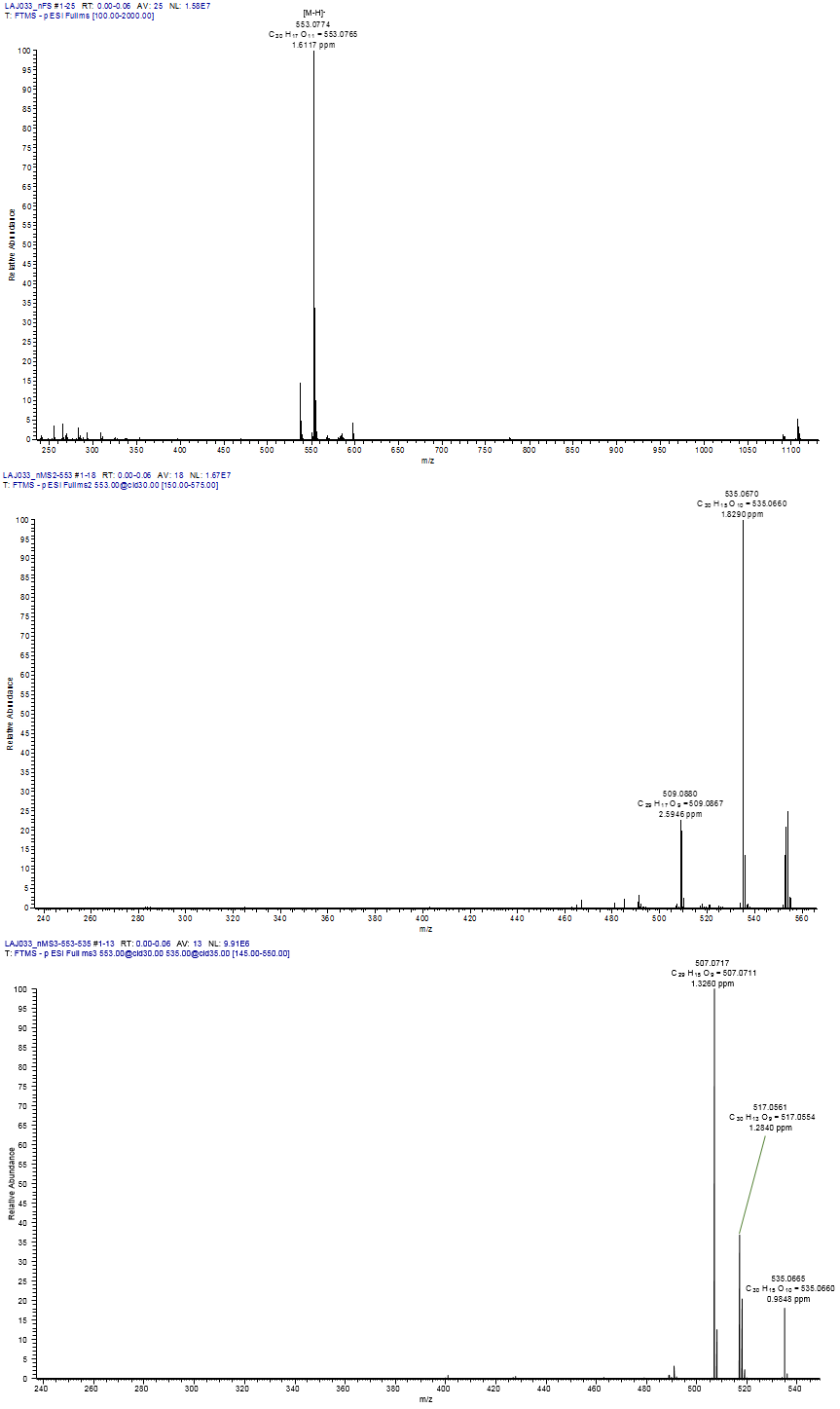
**

**Fig. S30** ^1^H NMR spectrum (500 MHz, CDCl_3_) of (+)-(*S*)-aurantioskyrin (**7**).


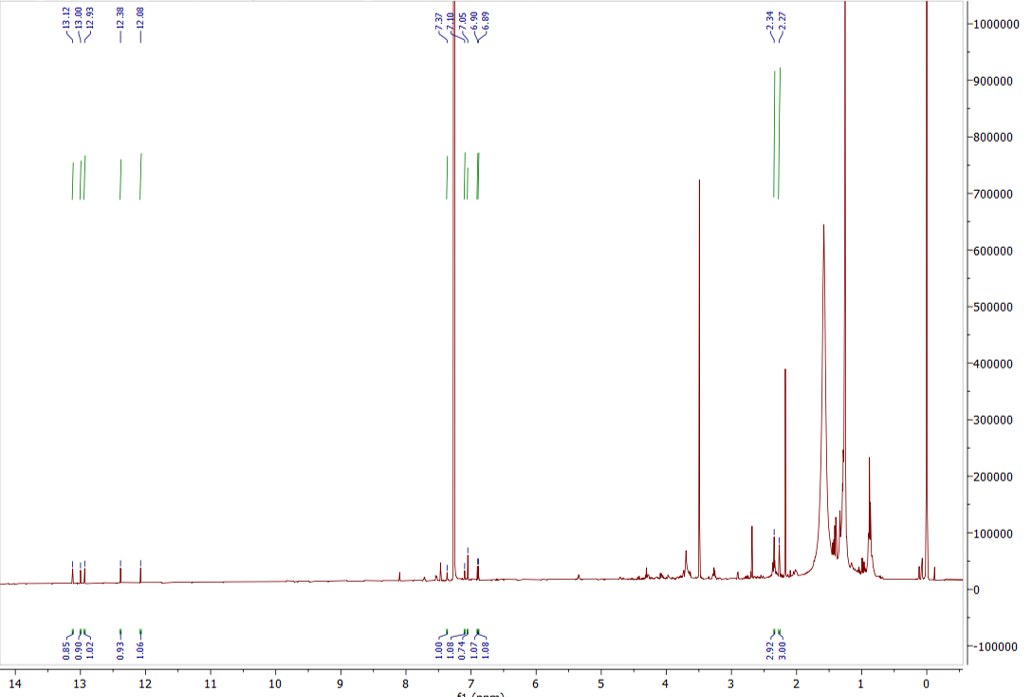


**Fig. S31** HSQC spectrum (500/125 MHz, CDCl_3_) of (+)-(*S*)-aurantioskyrin (**7**).
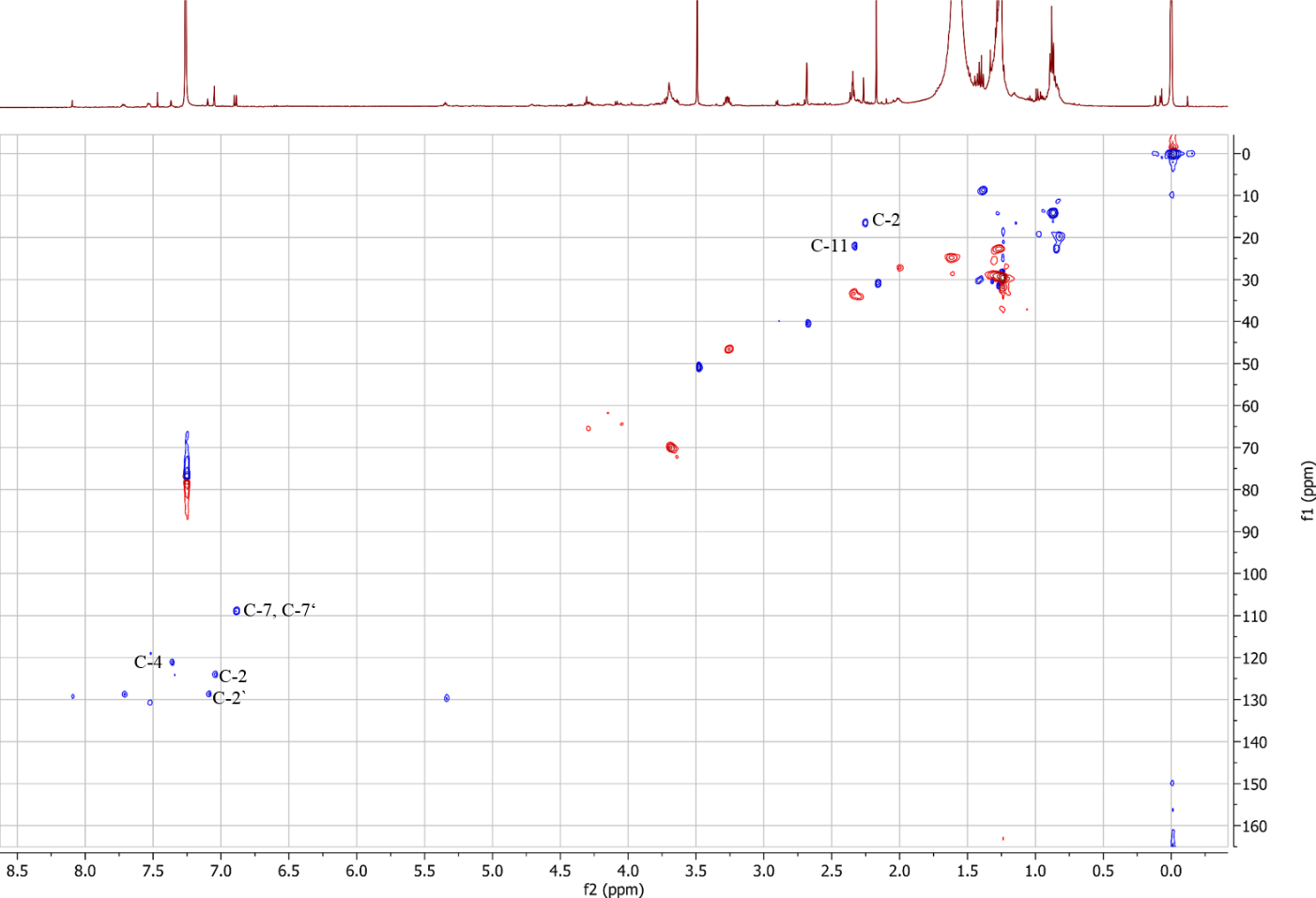


**Fig. S32** HMBC spectrum (500/125 MHz, CDCl_3_) of (+)-(*S*)-aurantioskyrin (**7**).

**
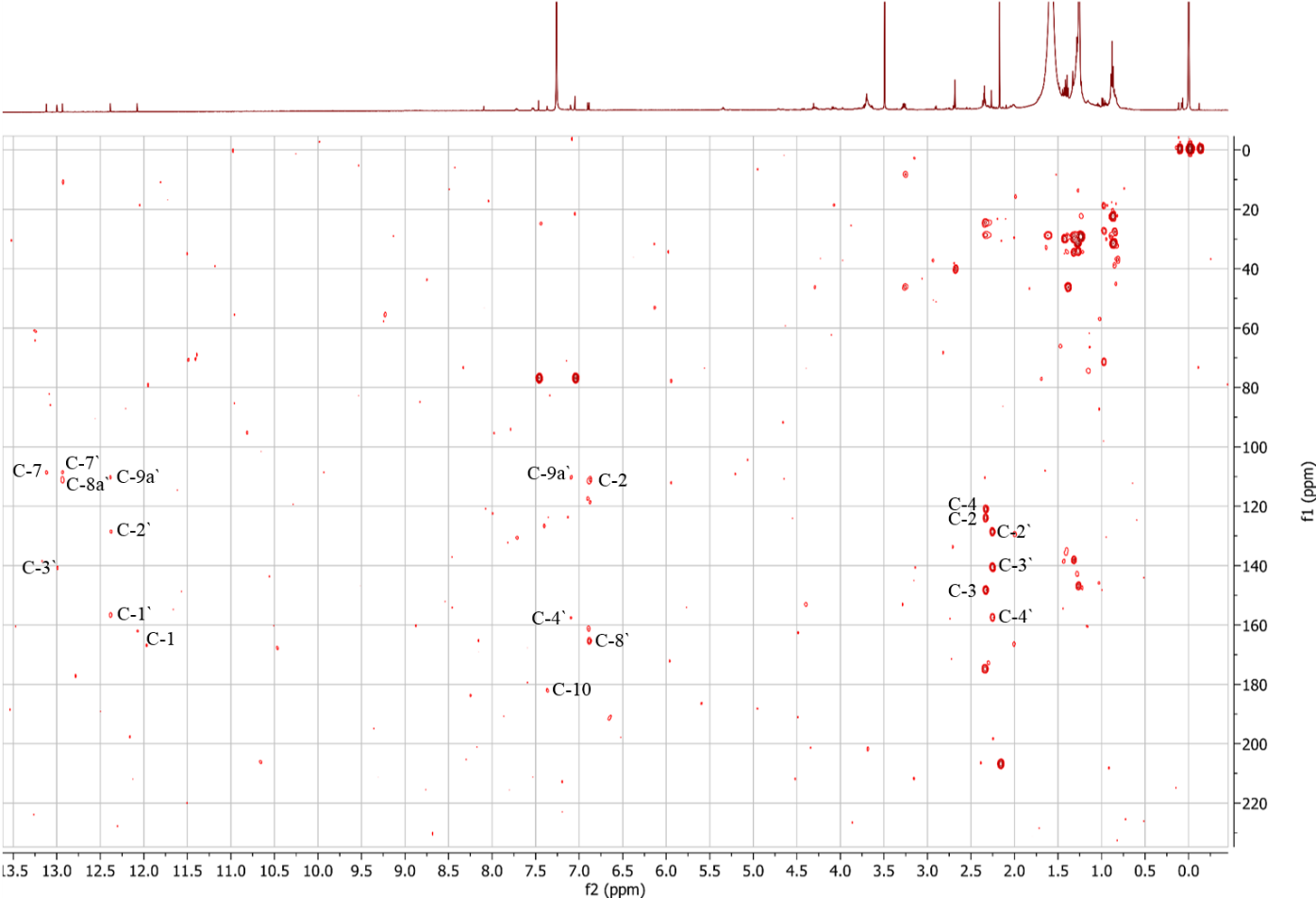
**

**Fig. S33** CD spectrum (MeOH) of (+)-(*S*)-aurantioskyrin (**7**).


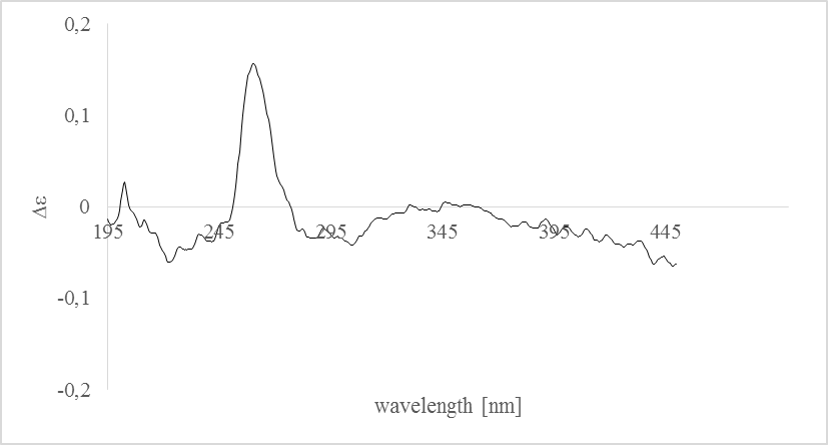


**Fig. S34** Negative ion HRESIMS^n^ spectra of hypericin (**8**).


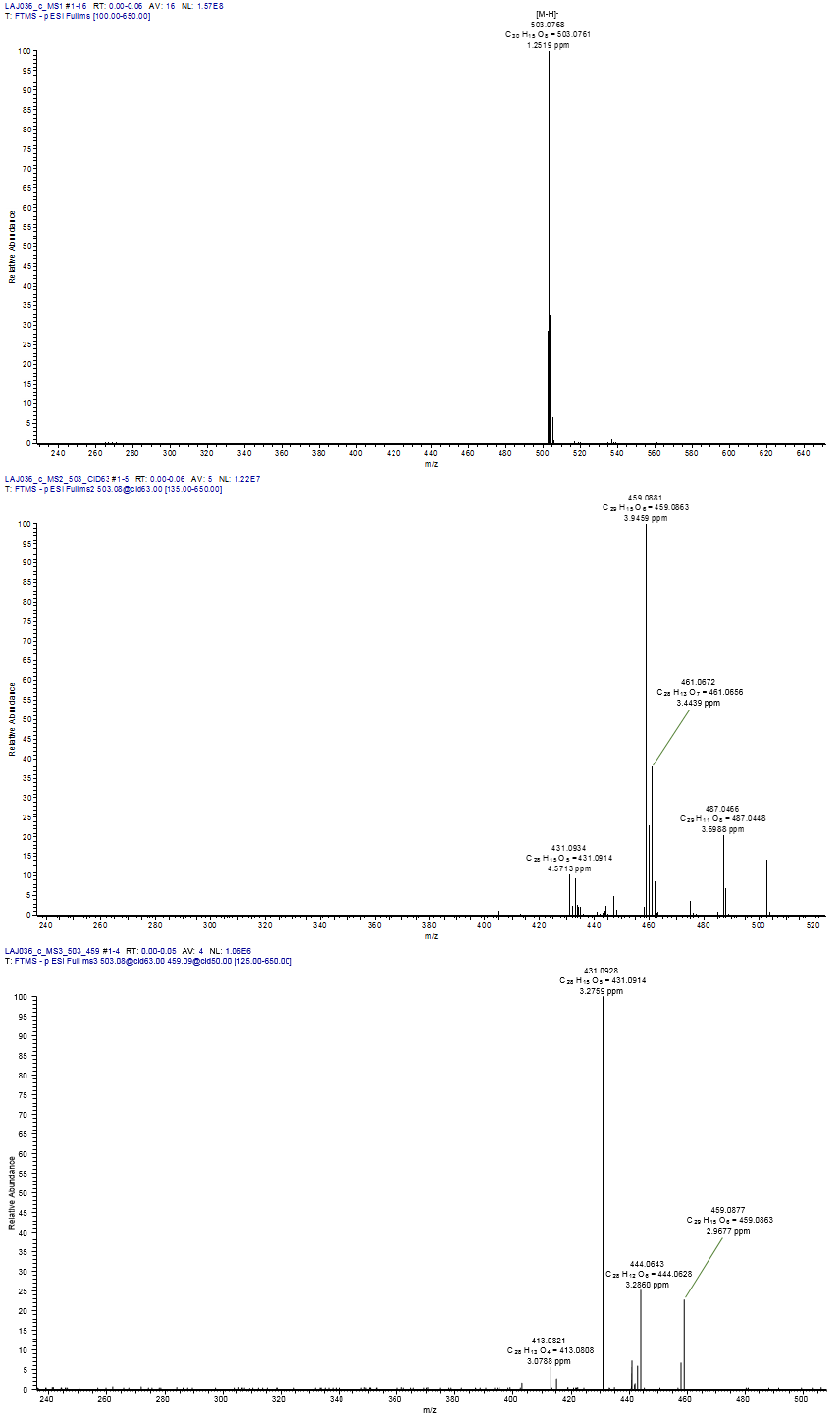


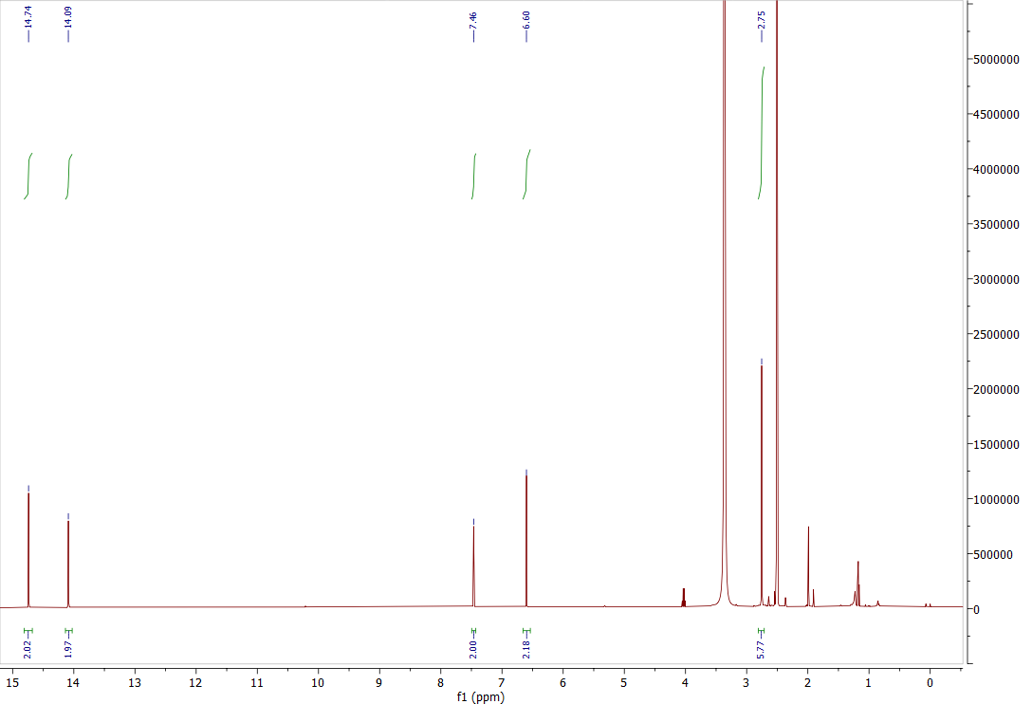
**Fig. S35** ^1^H NMR spectrum (500 MHz, DMSO-*d*_6_) of hypericin (**8**).


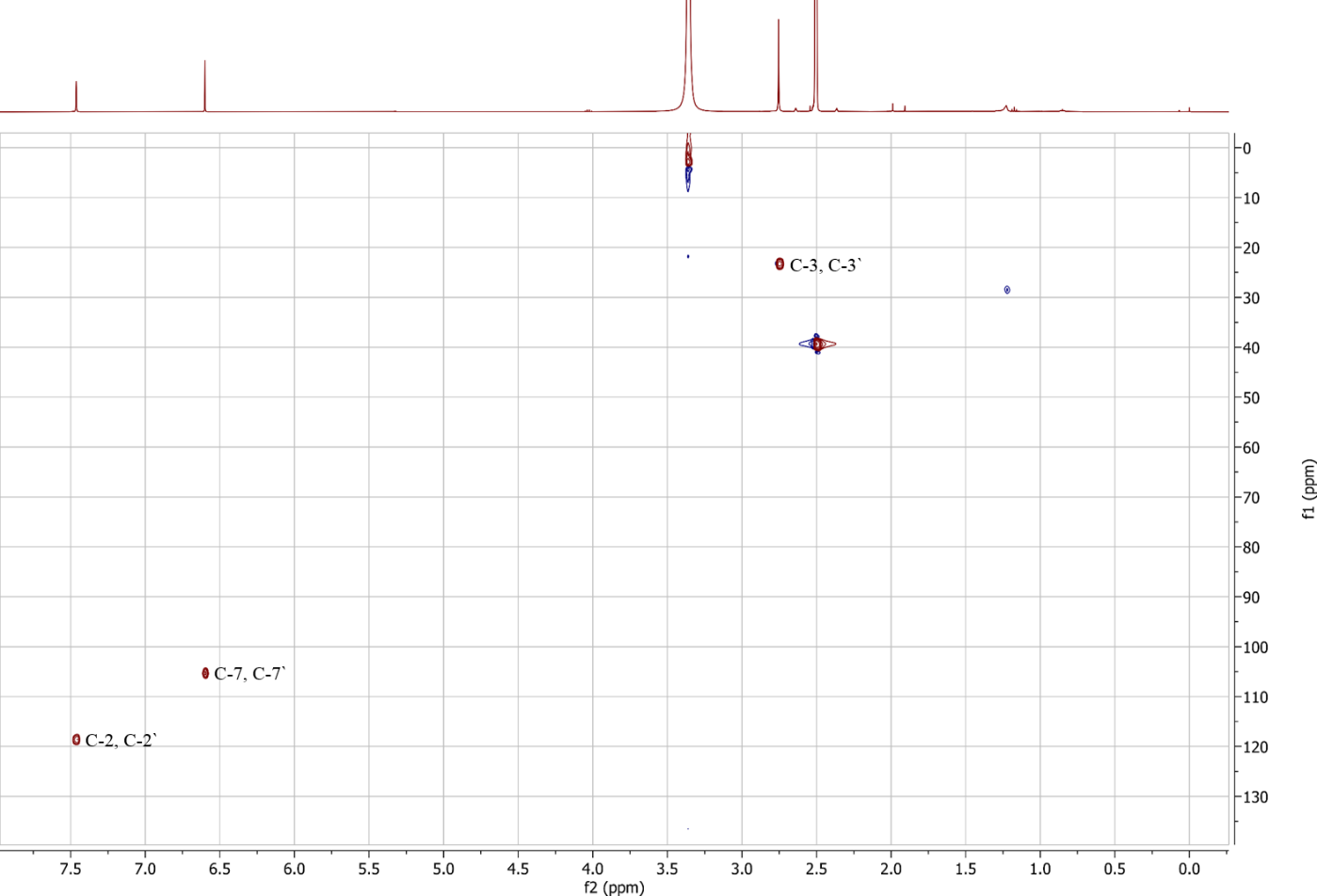
**Fig. S36** HSQC spectrum (500 MHz, DMSO-*d*_6_) of hypericin (**8**).


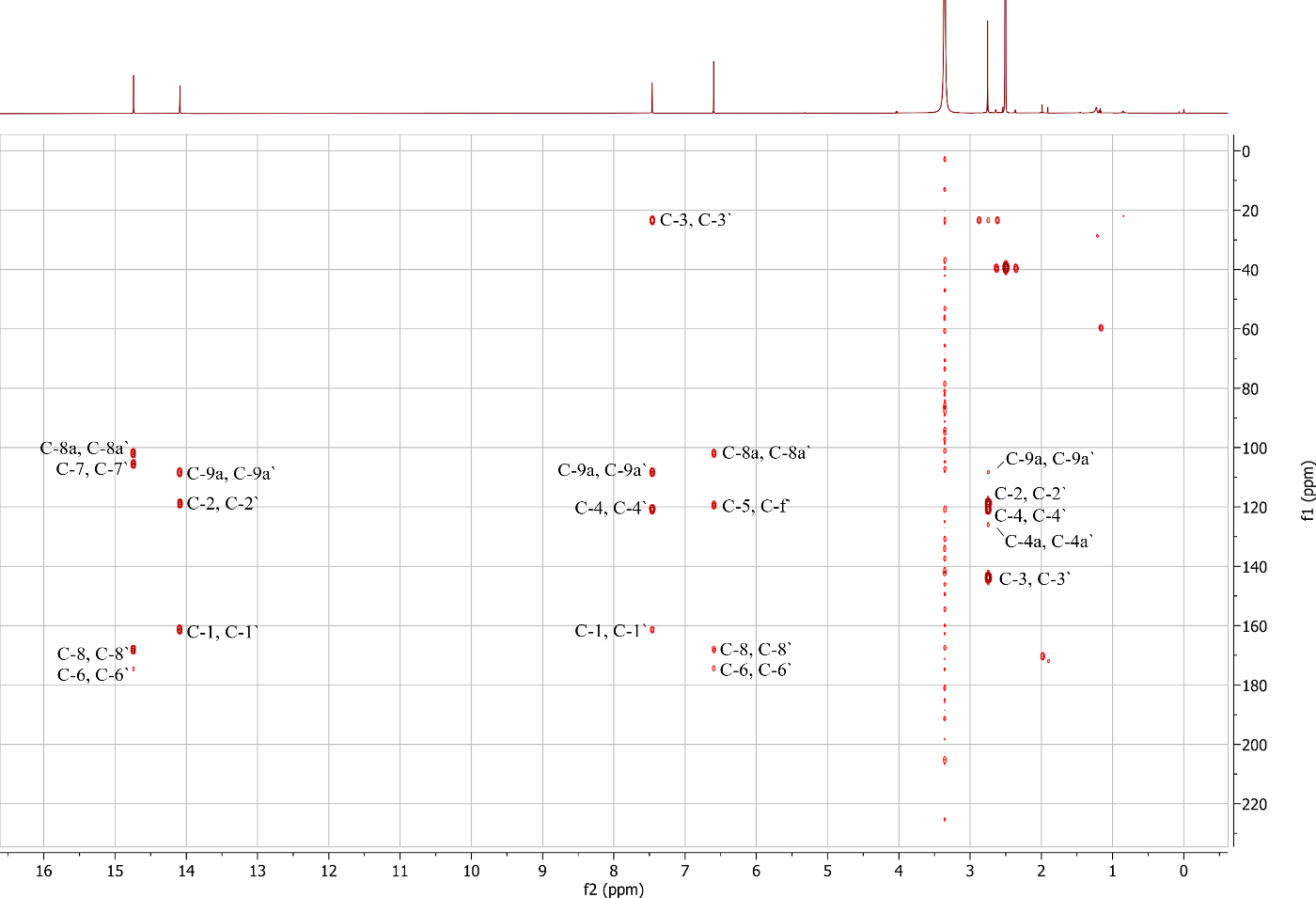
**Fig. S37** HMBC spectrum (500 MHz, DMSO-*d*_6_) of hypericin (**8**).

**Fig. S38** Negative ion HRESIMS^n^ spectra of dermolutein (**9**).

**
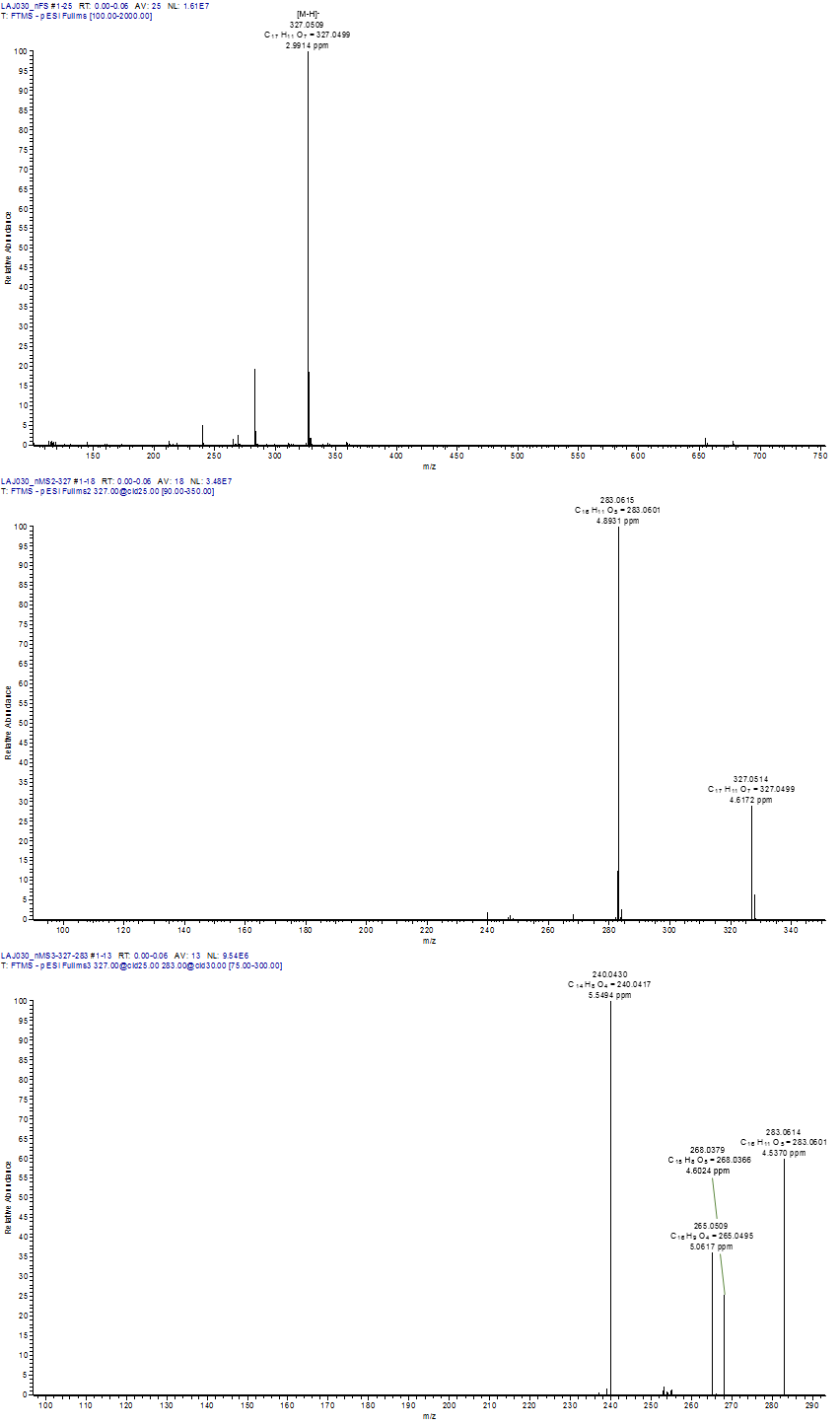
**

**Fig. S39** ^1^H NMR spectrum (500 MHz, CD_3_OD) of dermolutein (**9**).


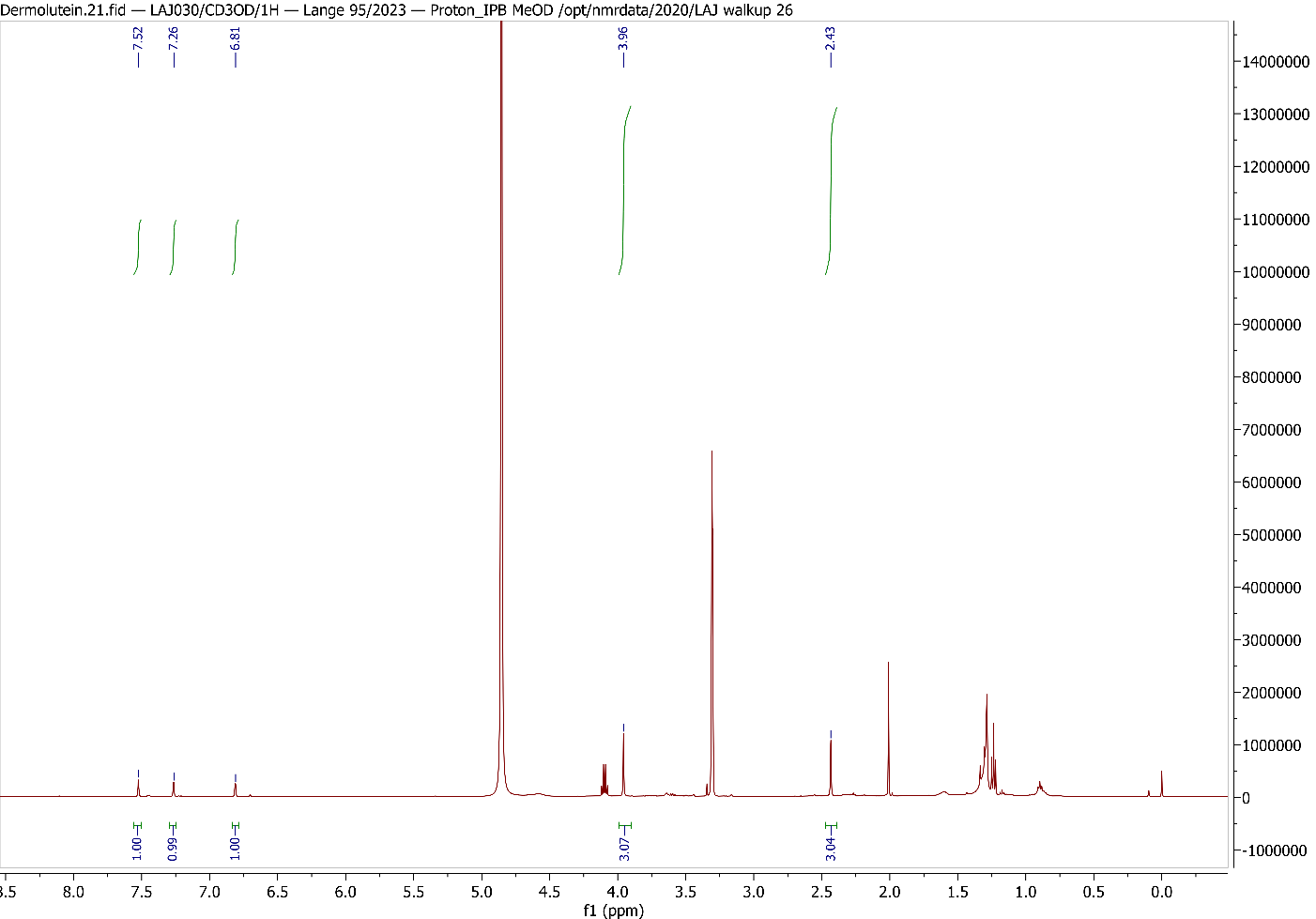


**Fig. S40** ^13^C NMR spectrum (125 MHz, CD_3_OD) of dermolutein (**9**).

**
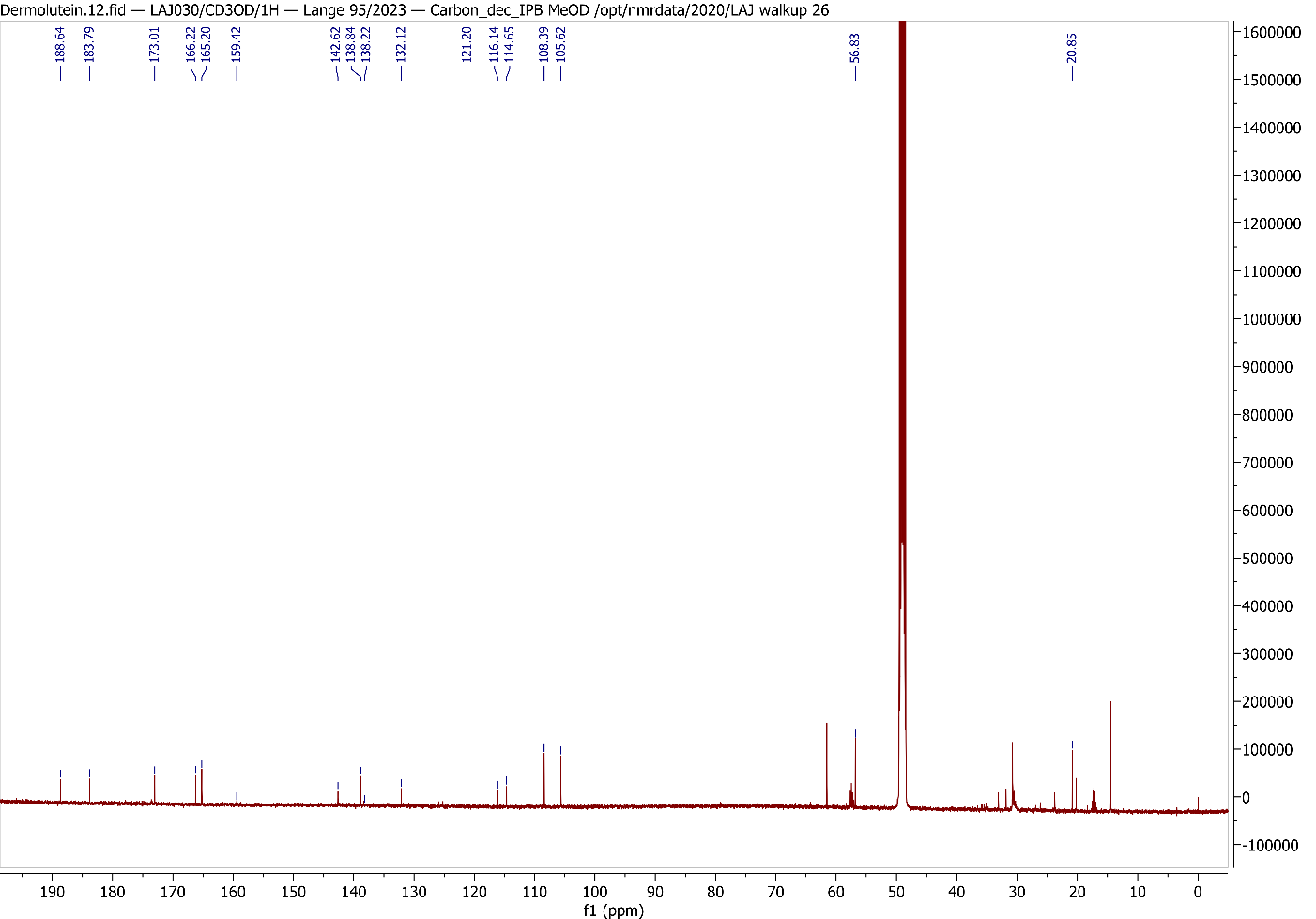
**

**Fig. S41** Negative ion HRESIMS^n^ spectra of endocrocin (**10**).


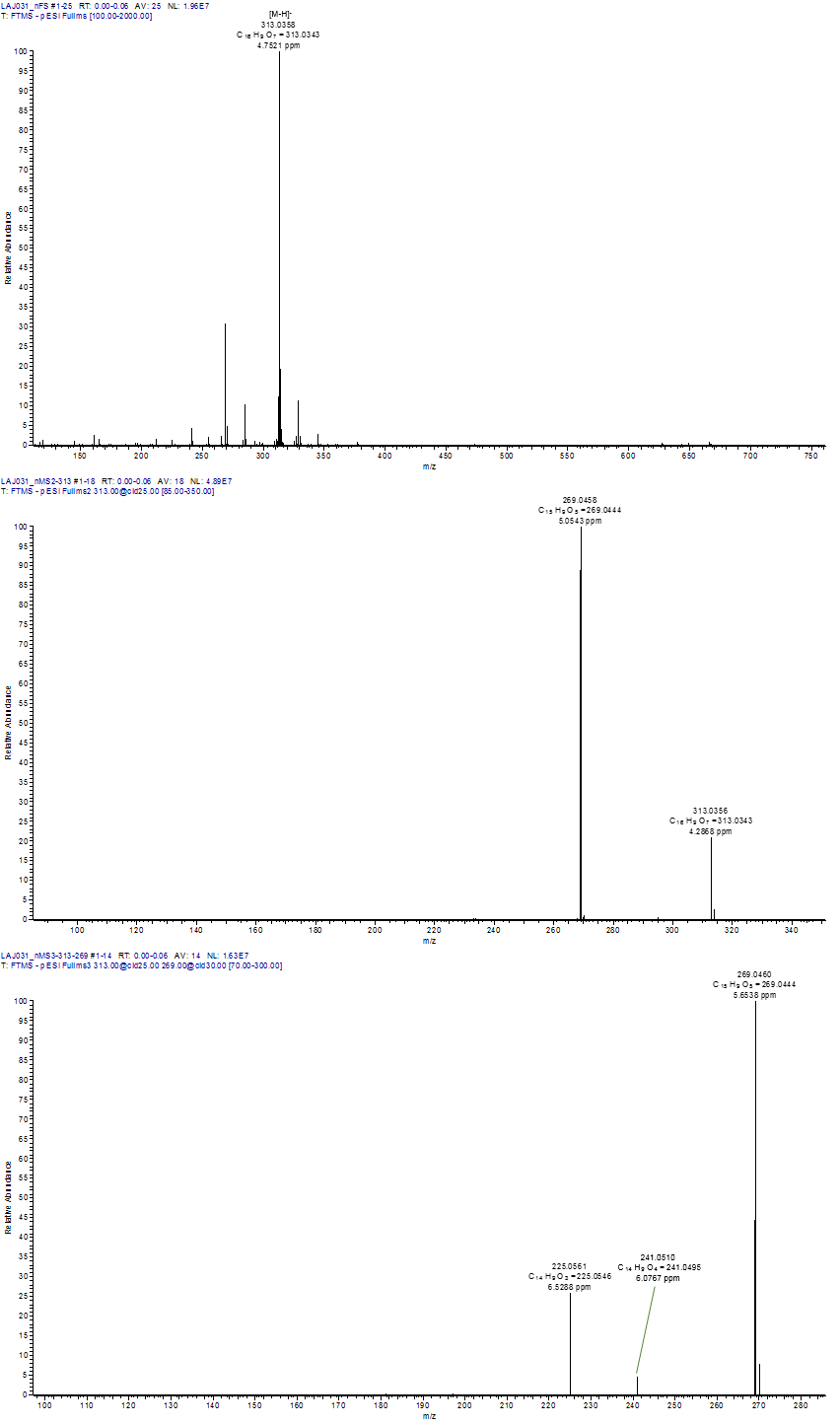


**Fig. S42** ^1^H NMR spectrum (500 MHz, CD_3_OD) of endocrocin (**10**).

**
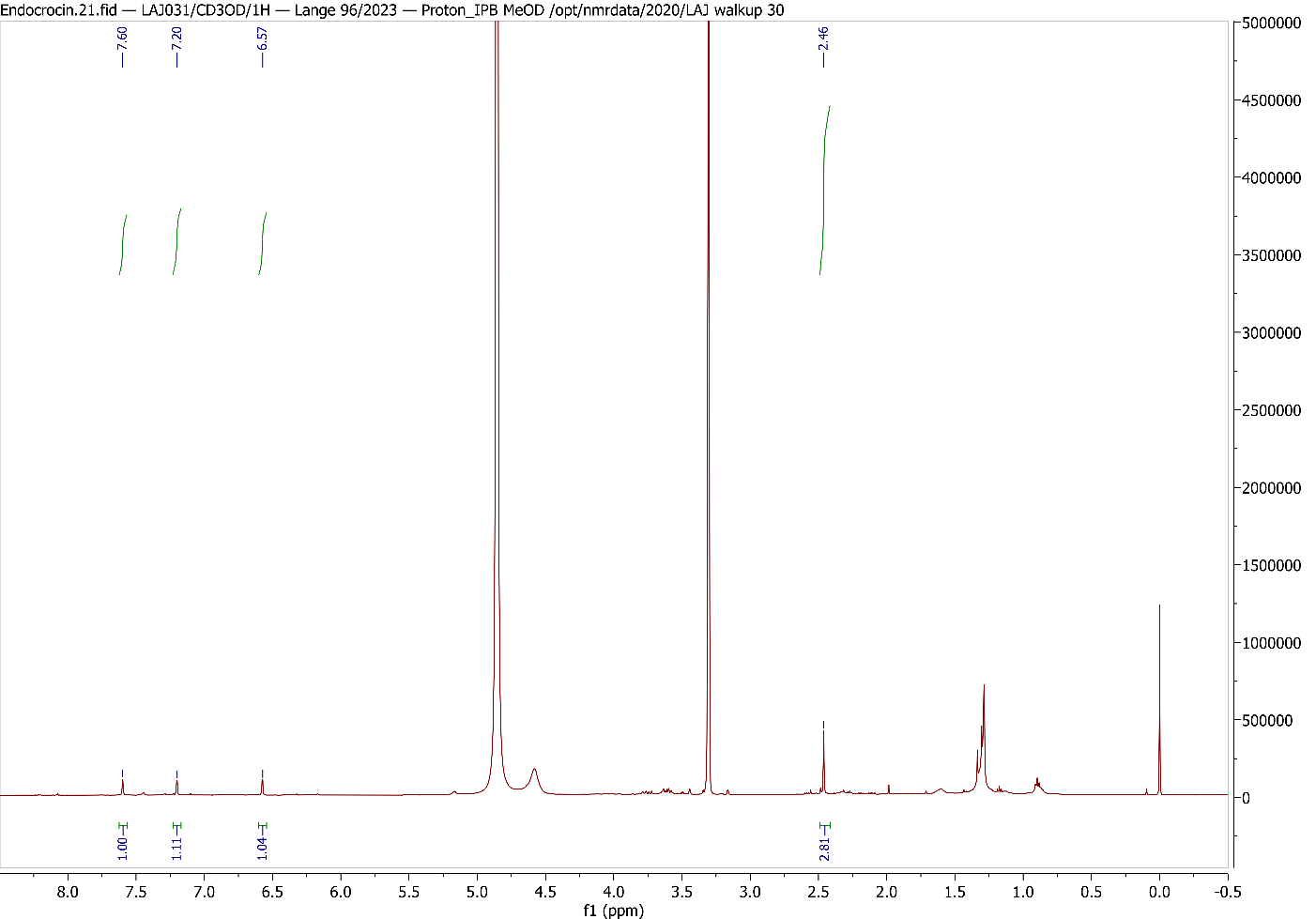
**

**Fig. S43** HSQC spectrum (500/125 MHz, CD_3_OD) of endocrocin (**10**).


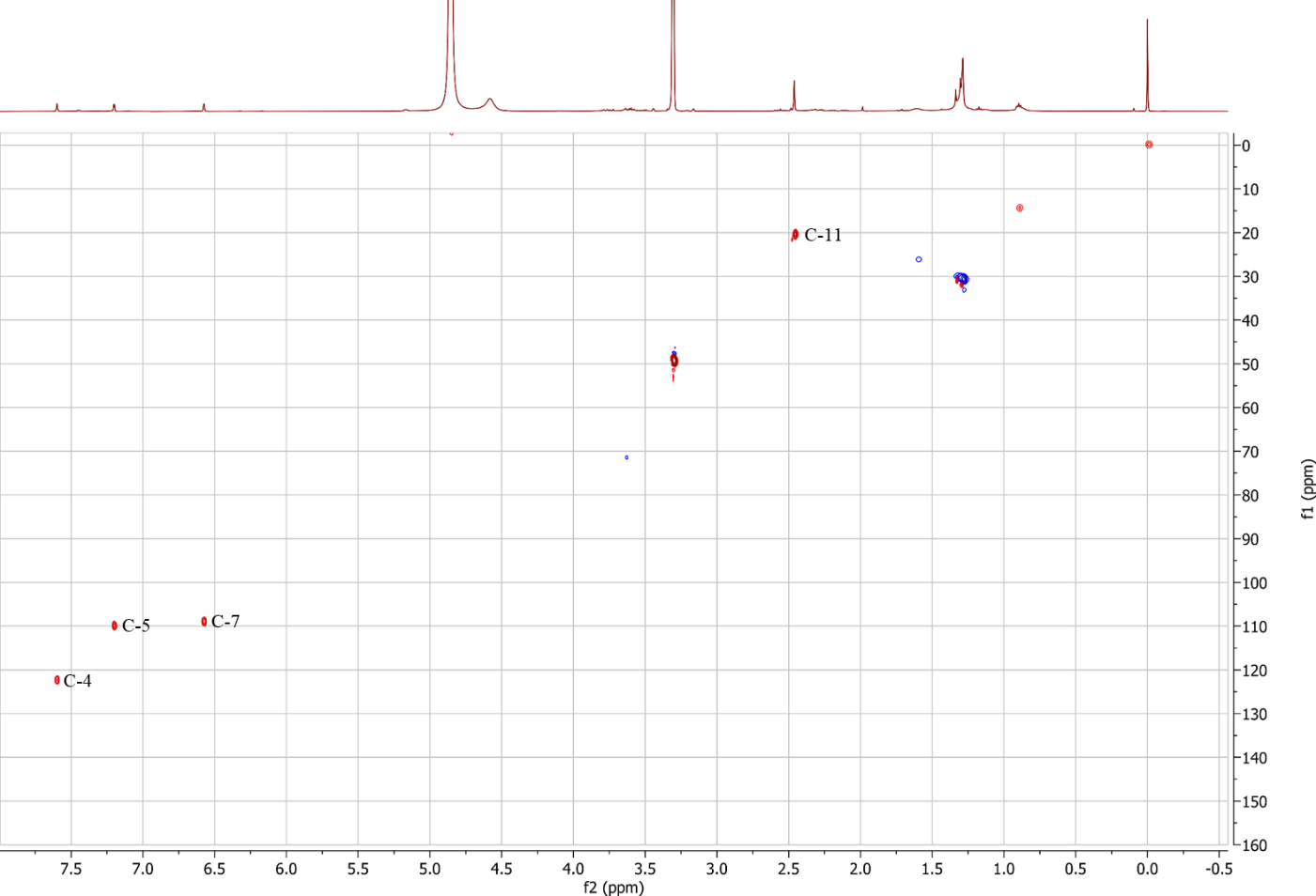


**Fig. S44** HMBC spectrum (500/125 MHz, CD_3_OD) of endocrocin (**10**).


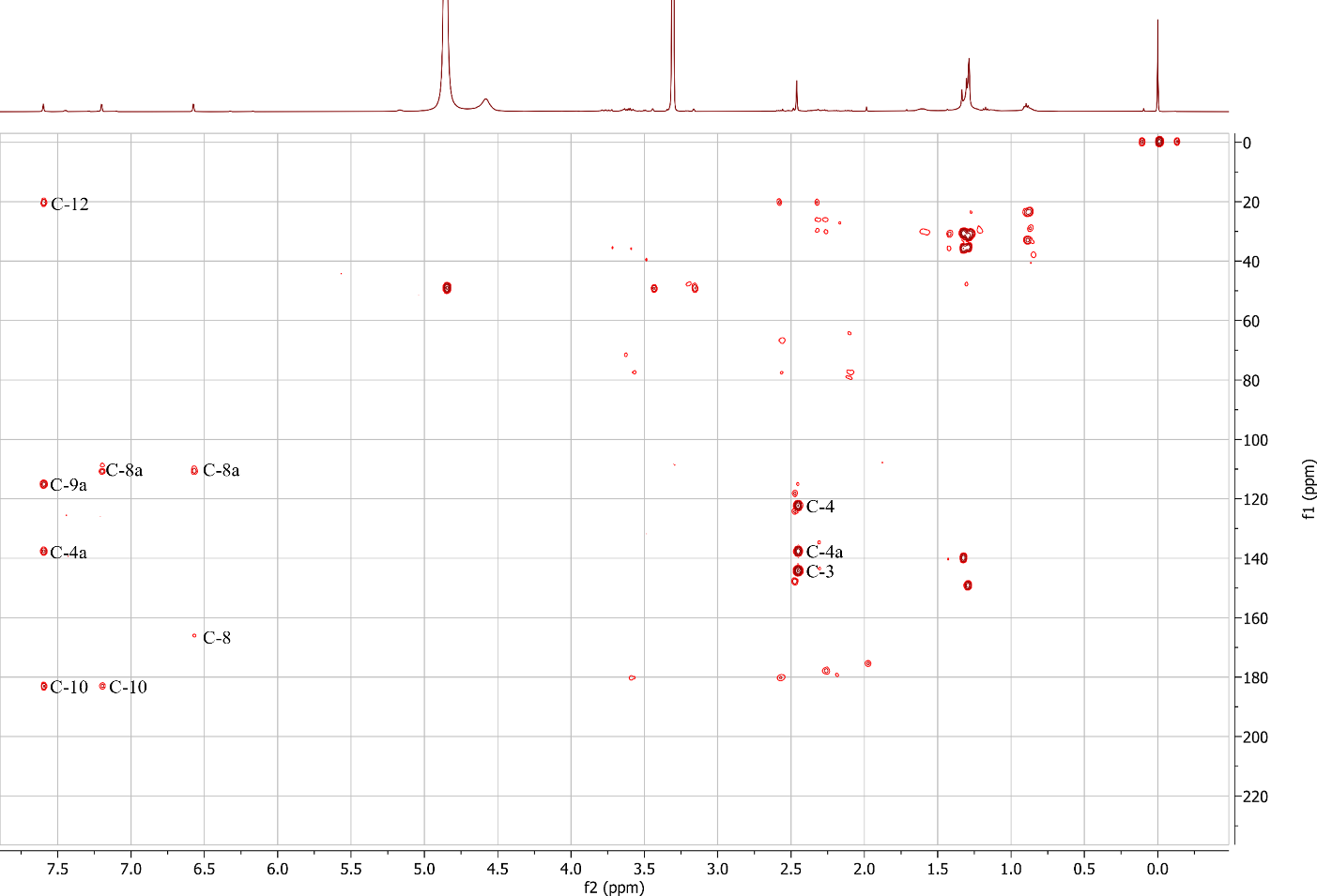


**Fig. S45** Negative ion HRESIMS^n^ spectra of clavorubin (**11**).

**
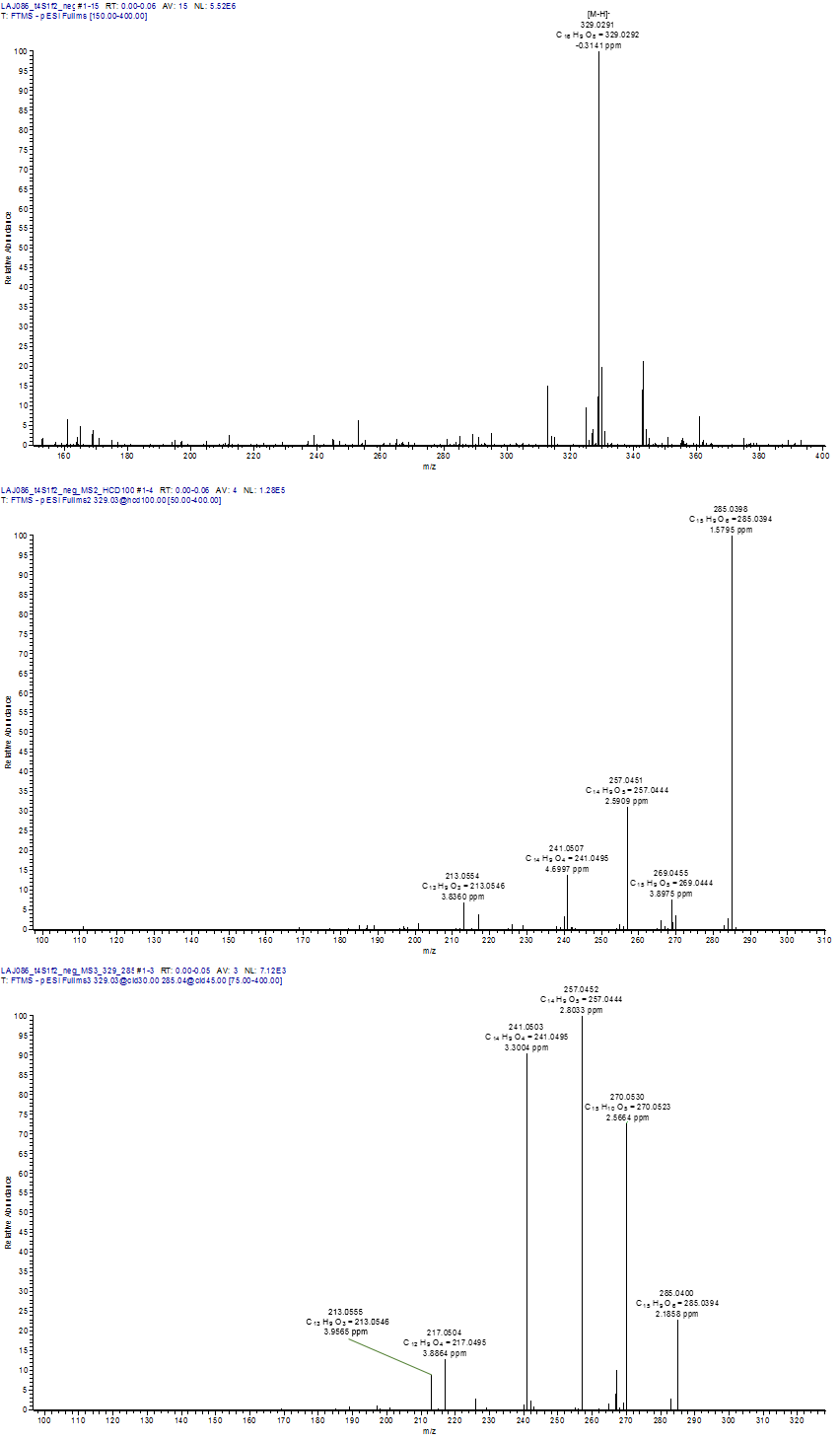
**

**Fig. S46 a** ^1^H NMR spectrum (500 MHz, DMSO-*d*_6_) of clavorubin (**11**).

**
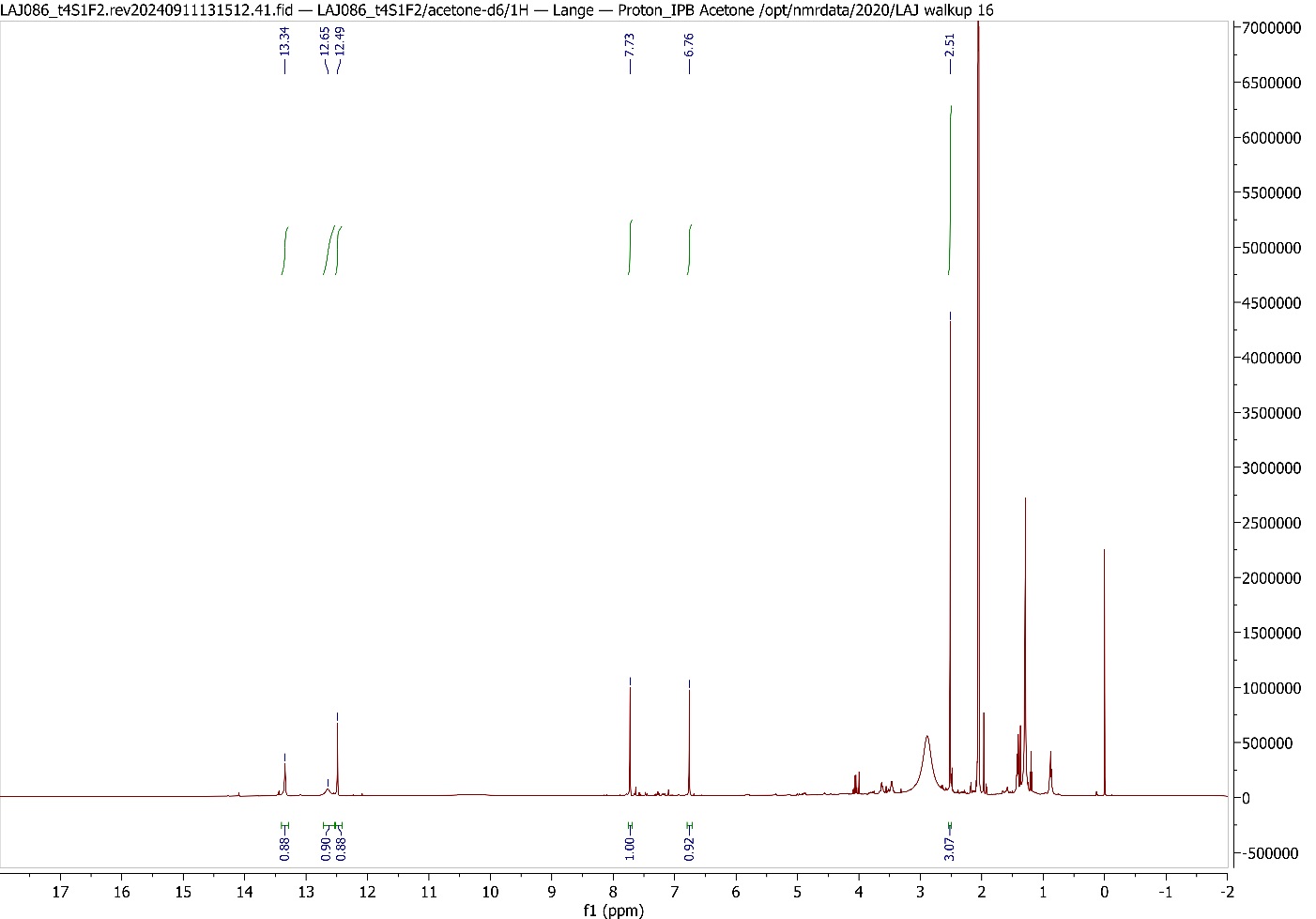
**

**Fig. S46 b** ^1^H NMR spectrum (500 MHz, THF-*d*_8_) of clavorubin (**11**).


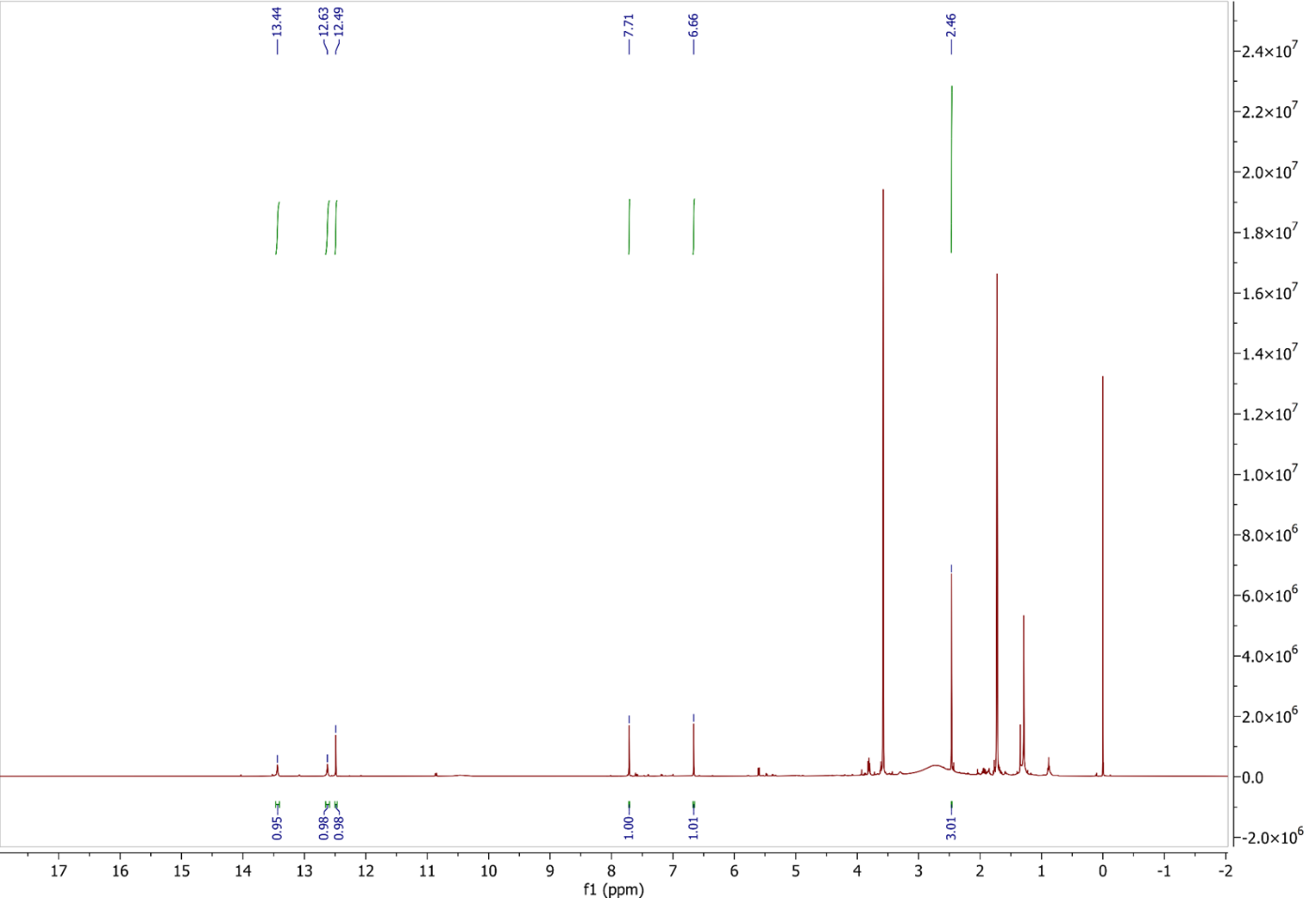


**Fig. S47** ^13^C NMR spectrum (125 MHz, THF-*d*_8_) of clavorubin (**11**).


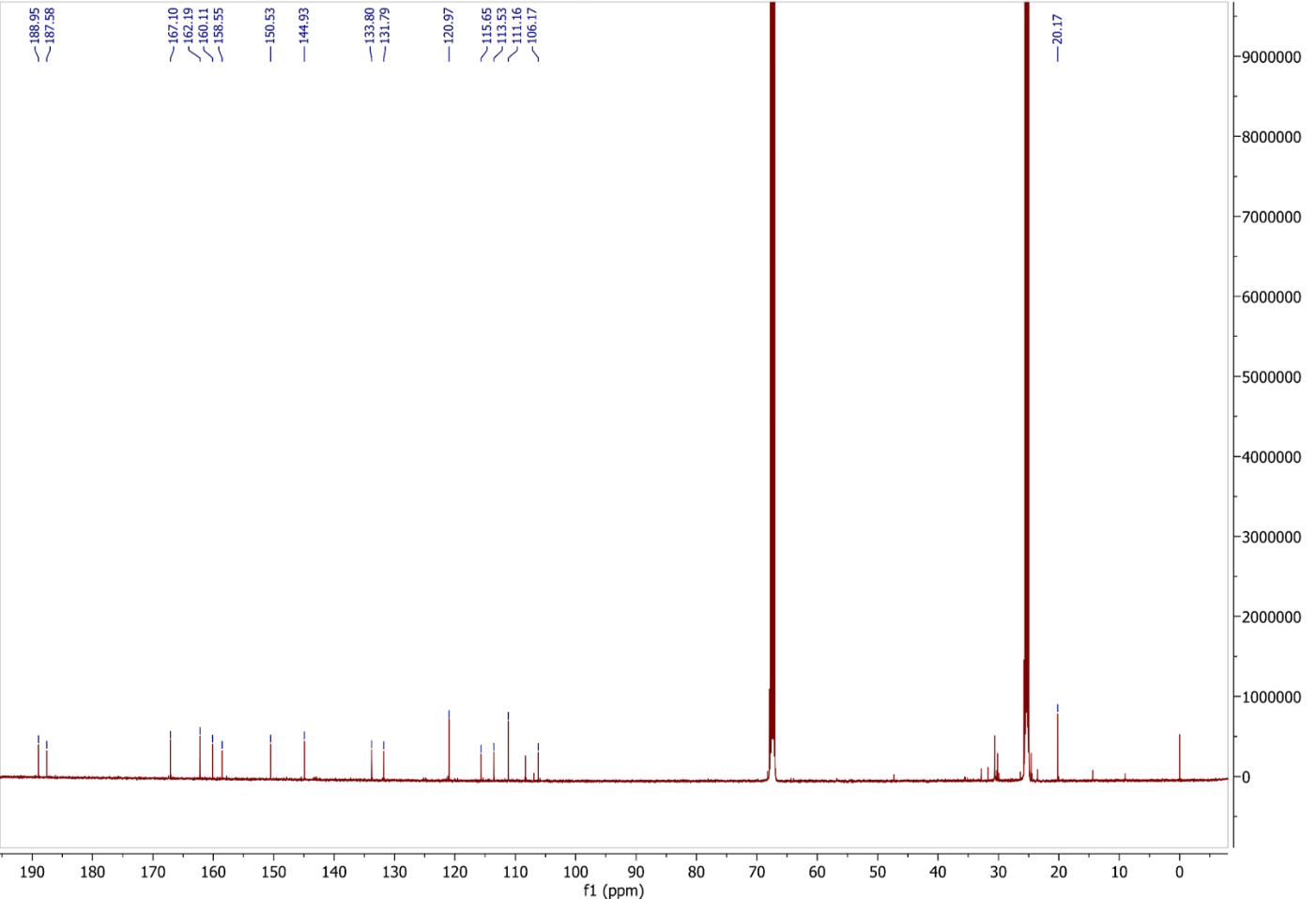


**Fig. S48** HSQC spectrum (500/125 MHz, THF-*d*_8_) of clavorubin (**11**).


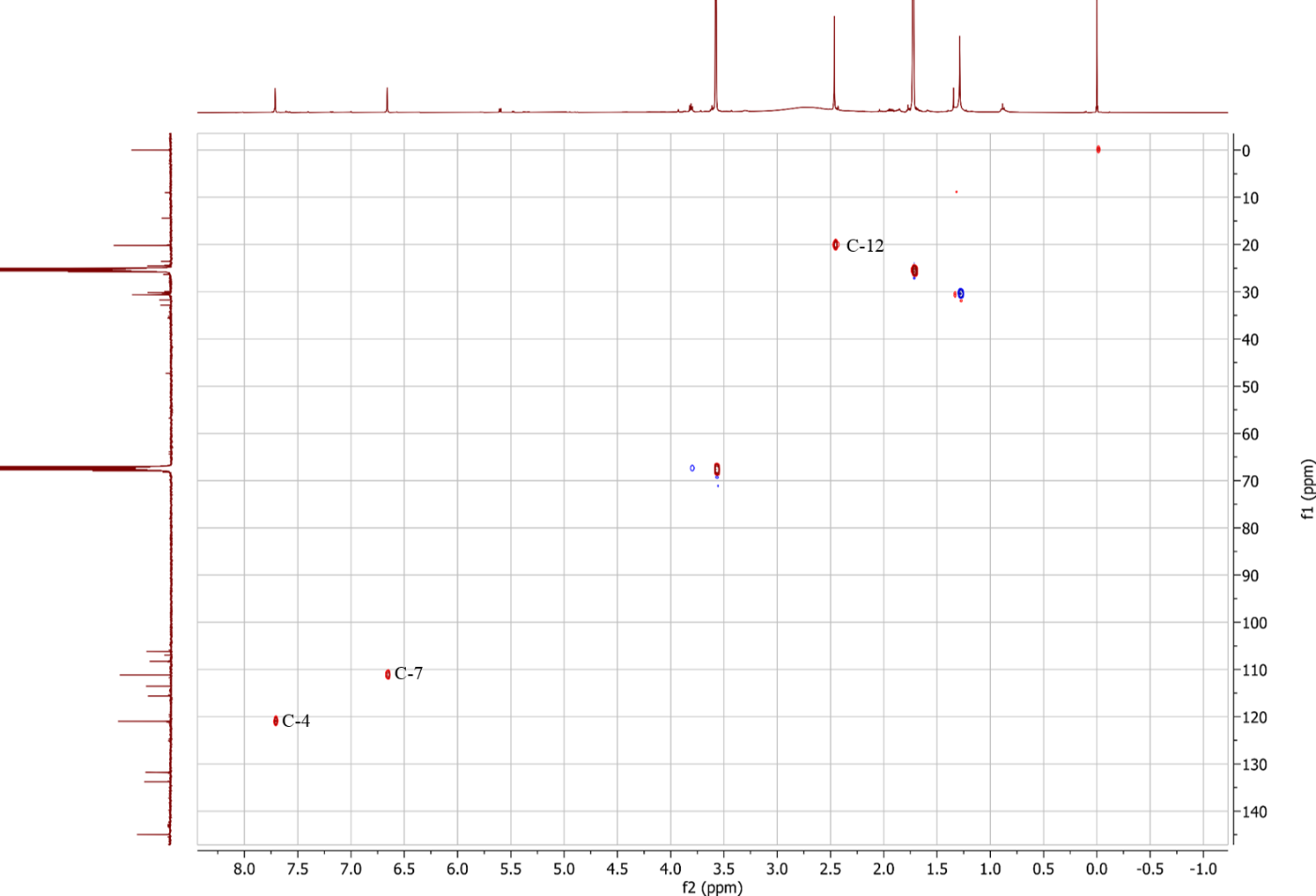


**Fig. S49** HMBC spectrum (500/125 MHz, THF-*d*_8_) of clavorubin (**11**).


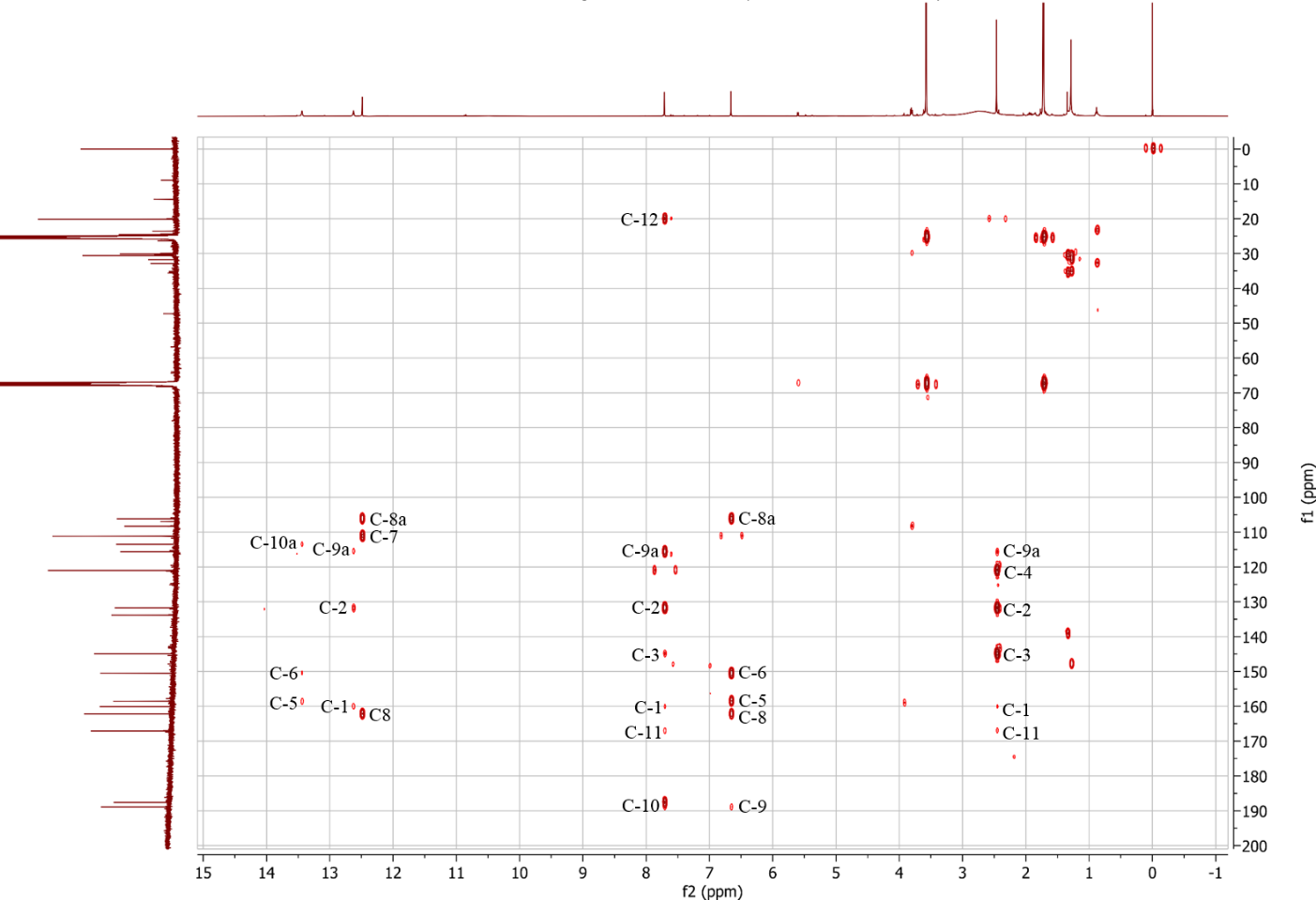


**Fig. S50** Maximum likelihood phylogenetic tree of concatenated ITS, LSU, and rpb1 data of selected *Cortinarius* taxa, for the placement of the new species. Shown is the best ML tree with bootstrap values (BS > 60) and Baysian posterior propabilities (BPP > 0.60) provided above the branches. The dataset contains 134 samples, and consist of nrITS, nrLSU, and rpb1-α sequences. The positions are: ITS: 1 – 815, LSU: 816 – 1765, rpb1: 1766 – 2282. Voucher numbers and sections are included in labels. The new species *Cortinarius mapuveronicae* is printed in red, other species sequenced within this study are printed in pink.**
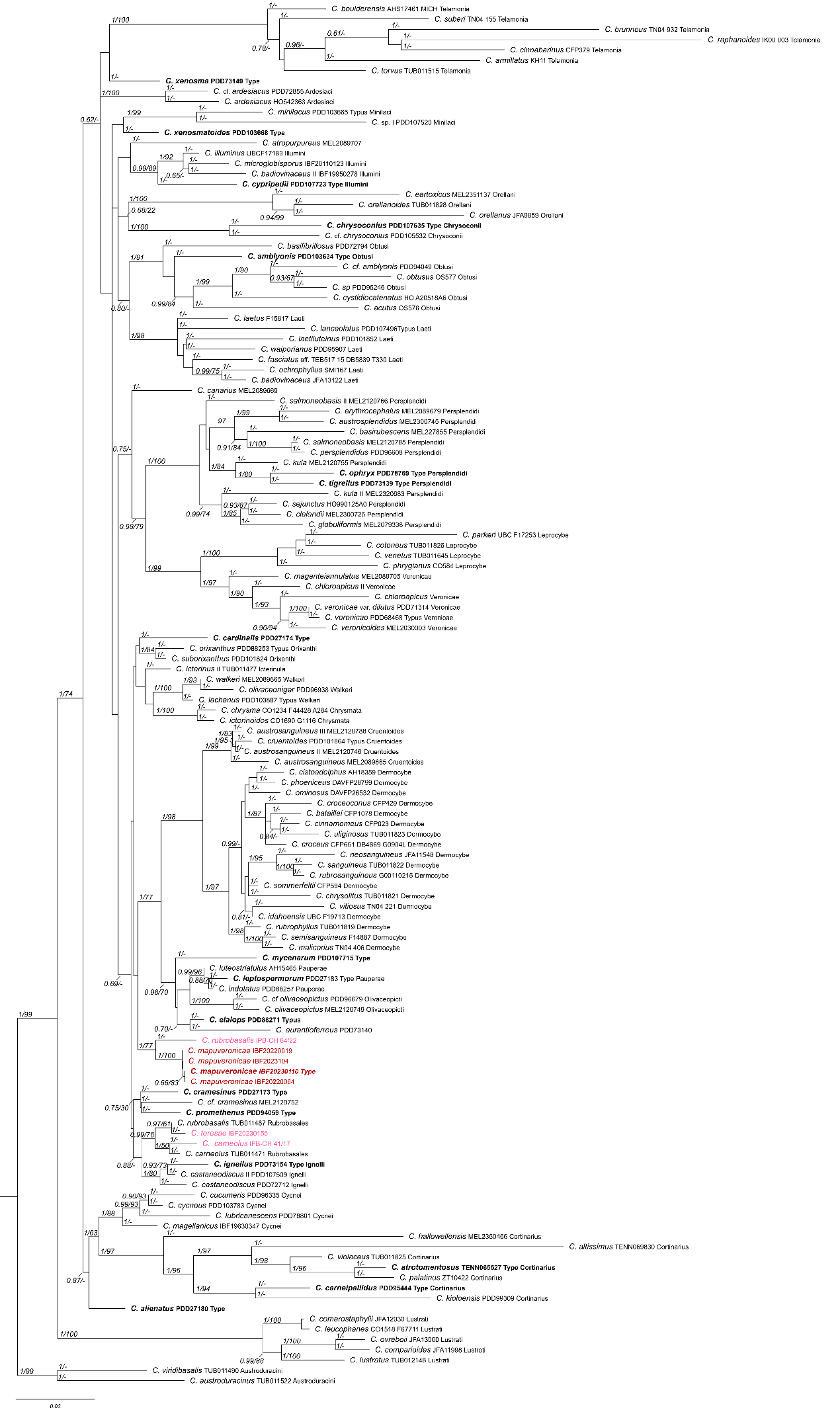
**

**Fig. S51** Isolation scheme.

**
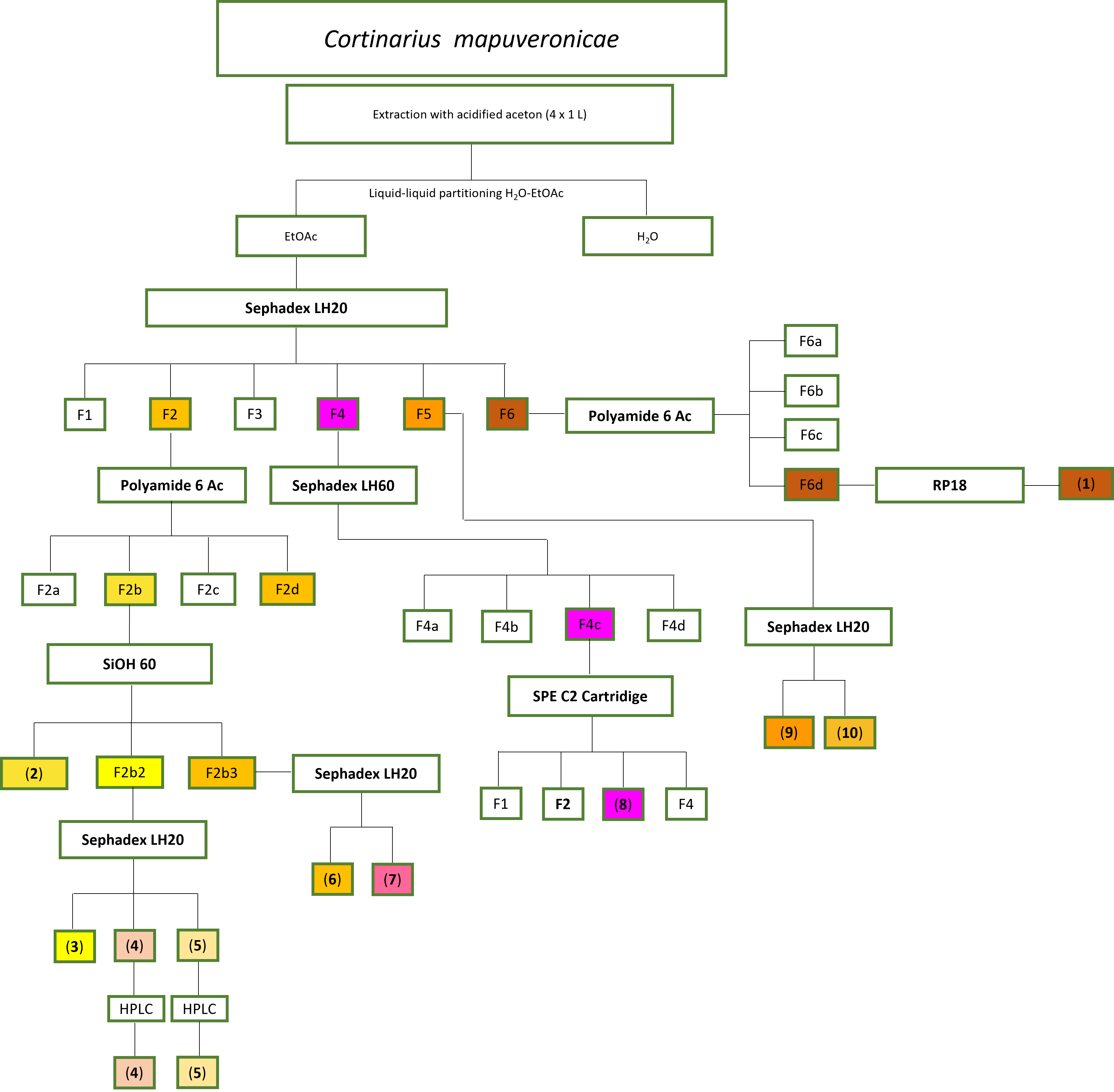
**

**Fig. S52** Cell growth inhibition curves of the active anthraquinoid compounds **1**, **3**, **7**, and **8**.


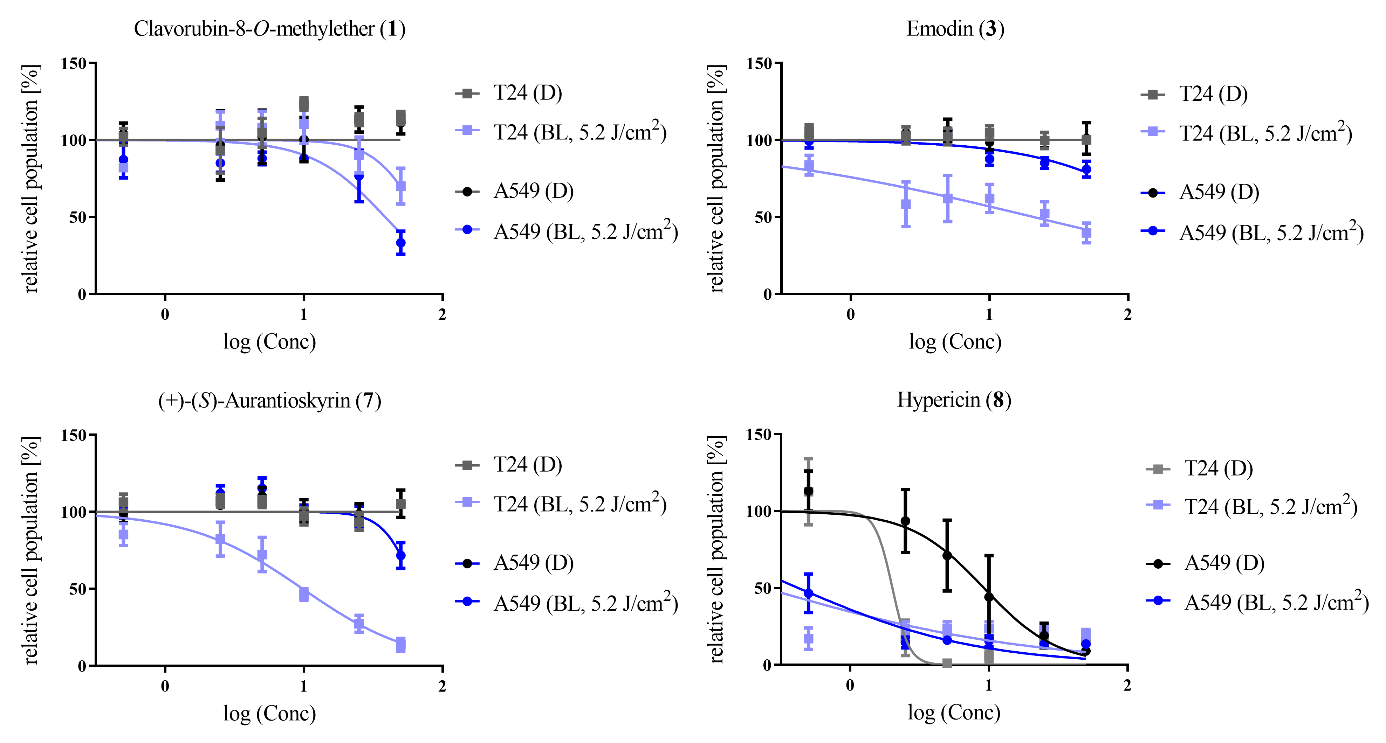


**Fig. S53** Growth inhibition curves of the active anthraquinoid compounds **3**, **7**, and **8**.


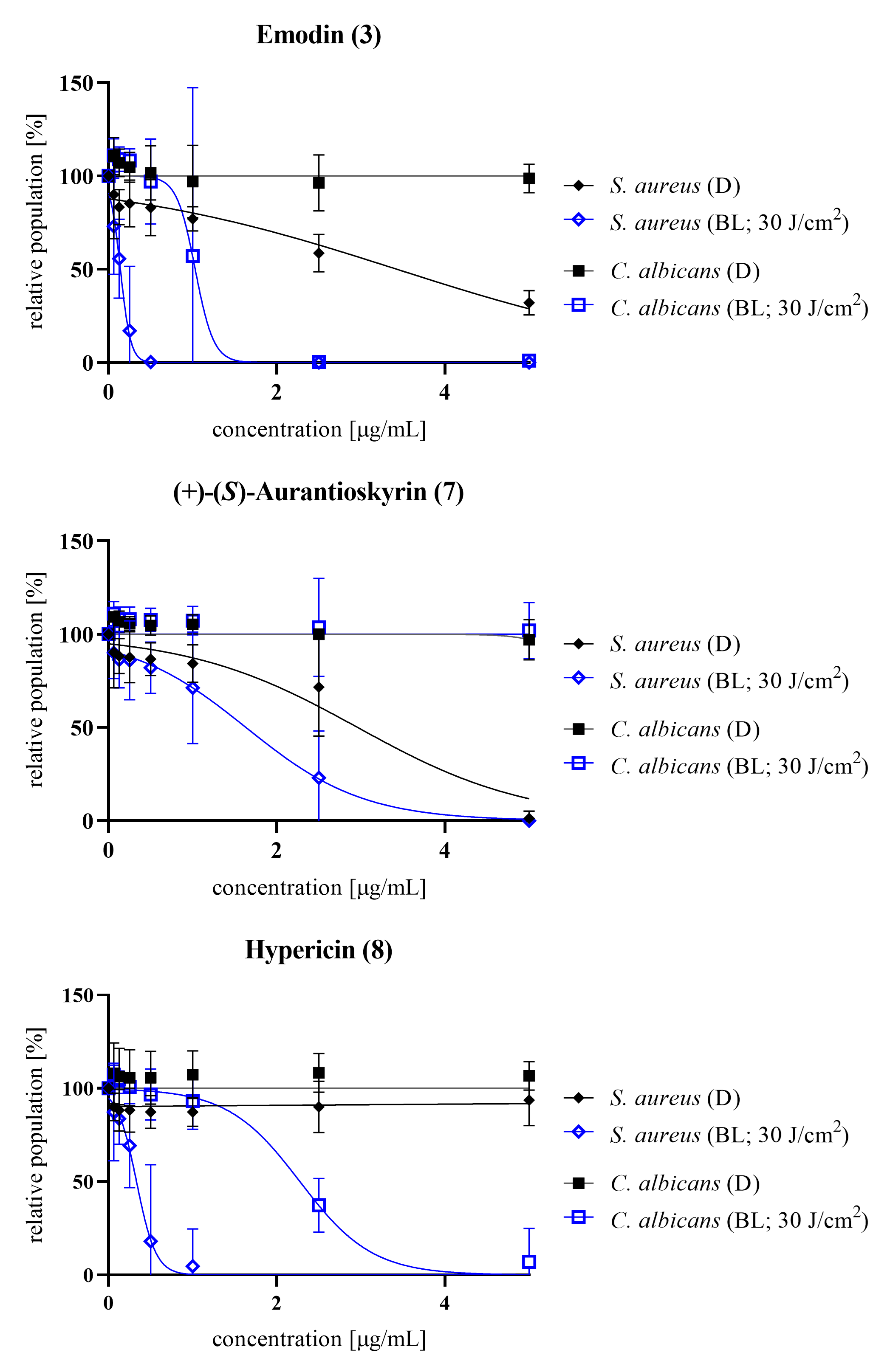


**Tab. S1** Collections examined in this study with Genbank Accession numbers, voucher numbers, and collection data.

| **Genbank number** | **Herbar number** | **Name** | **Collection date** | **Collection location** | **leg. & det.** | **GPS-lat** | **GPS-long** |
| --- | --- | --- | --- | --- | --- | --- | --- |
| ITS: PX230457, LSU: PX230475 | IPB-CHL 41/17 | *Cortinarius carneolus* | 5.2017 | Chile, Chanco, Cauquenes Province | N. Arnold | 35°50'01.9"S | 72°30'30.2"W |
| ITS: PX230439, LSU: PX230468, RPB1: PX240688 | IBF20230110 | ***Cortinarius mapuveronicae* TYPE** | 09.04.2023 | Chile, Patagonia, Chaíten | L. Huymann, B. Siewert, U. Peintner | 43°20'55.93''S | 73°10'18.73''W |
| ITS: PX230432, LSU: PX230462 RPB1: PX240687 | IBF20220019 | *Cortinarius mapuveronicae* | 14.05.2022 | Chile, Llancacura | U. Peintner, L. Huymann, N. Arnold | 40°13'32.92''S | 73°21'35.63''W |
| ITS: PX230433 | IBF20220020 | *Cortinarius mapuveronicae* | 14.05.2022 | Chile, Llancacura | U. Peintner, L. Huymann, N. Arnold | 40°13'32.92''S | 73°21'35.63''W |
| ITS: PX230434, LSU: PX230463, RPB1: PX240689 | IBF20220064 | *Cortinarius mapuveronicae* | 17.05.2022 | Chile, Curanilahue - Trongo Alto | U. Peintner, L. Huymann, N. Arnold | 37°41'23.21''S | 73°21'35.85''W |
| ITS: PX230435, LSU: PX230464 | IBF20220073A | *Cortinarius mapuveronicae* | 18.05.2022 | Chile, Cañete | U. Peintner, L. Huymann, N. Arnold | 37°41'23.21''S | 73°21'35.85''W |
| ITS: PX230436, LSU: PX230465 | IBF20220073B | *Cortinarius mapuveronicae* | 18.05.2022 | Chile, Cañete | U. Peintner, L. Huymann, N. Arnold | 37°41'23.21''S | 73°21'35.85''W |
| LSU: PX230466 | IBF20220073C | *Cortinarius mapuveronicae* | 18.05.2022 | Chile, Cañete | U. Peintner, L. Huymann, N. Arnold | 37°41'23.21''S | 73°21'35.85''W |
| ITS: PX230437 | IBF20220104 | *Cortinarius mapuveronicae* | 21.05.2022 | Chile, Curanilahue | U. Peintner, L. Huymann, N. Arnold | 37°41'23.21''S | 73°21'35.85''W |
| ITS: PX230438, LSU: PX230467 | IBF20230090 | *Cortinarius mapuveronicae* | 09.04.2023 | Chile, Patagonia, Chaíten | L. Huymann, B. Siewert, U. Peintner | 43°20'55.93''S | 73°10'18.73''W |
| ITS: PX230440, LSU: PX230469 | IBF20230111 | *Cortinarius mapuveronicae* | 09.04.2023 | Chile, Patagonia, Chaíten | L. Huymann, B. Siewert, U. Peintner | 43°20'48.41''S | 72°23'59.92''W |
| ITS: PX230441 | IBF20230112 | *Cortinarius mapuveronicae* | 09.04.2023 | Chile, Patagonia, Chaíten | L. Huymann, B. Siewert, U. Peintner | 43°20'48.41''S | 72°23'59.92''W |
| ITS: PX230442, LSU: PX230470 | IBF20230104 | *Cortinarius mapuveronicae* | 09.04.2023 | Chile, Patagonia, Chaíten | L. Huymann, B. Siewert, U. Peintner | 43°20'48.41''S | 72°23'59.92''W |
| ITS: PX230443, LSU: PX230471 | CONCF2088a | *Cortinarius mapuveronicae* | 18.06.2022 | Chile, Curanilahue, Trongol Alto | G.Palfner,J. Farias | 37°31'21.72''S | 72°23'58.16''W |
| ITS: PX230444, LSU: PX230472 | CONCF2088b | *Cortinarius mapuveronicae* | 26.06.2022 | Chile, Curanilahue, Trongol Alto | G.Palfner, J. Farias | 37°31'21.7''S | 72°23'58.16''W |
| ITS: PX230445 | CONCF2069 | *Cortinarius mapuveronicae* | 28.05.2022 | Chile, Cañete, Butamalal | G.Palfner, J. Farias | 37°31'21.7''S | 72°23'58.16''W |
| ITS: PX230446 | CONCF1787 | *Cortinarius mapuveronicae* | 21.05.2017 | Chile, Los Álamos, Caramahuida | N. Arnold | 37°31'21.7''S | 72°23'58.16''W |
| ITS: PX230447 | IPB-CHL 9/18 | *Cortinarius mapuveronicae* | 03.05.2018 | Chile, La Union, Ranco Province | N. Arnold | 40°13'32.1"S | 73°21'36.9"W |
| ITS: PX230448 | IPB-CHL 46/18 | *Cortinarius mapuveronicae* | 01.05.2018 | Chile, Nahuelbuta, Arauco Province, Parque Nacional Nahuelbuta | N. Arnold | 37°49'38.1"S | 73°00'36.6"W |
| ITS: PX230449 | IPB-CHL 61/18 | *Cortinarius mapuveronicae* | 26.05.2018 | Chile, Nahuelbuta, Arauco Province, Parque Nacional Nahuelbuta | N. Arnold | 37°49'39.1"S | 73°00'35.6"W |
| ITS: PX230450 | IPB-CHL 11/22 | *Cortinarius mapuveronicae* | 30.04.2022 | Chile, La Union, Ranco Province | N. Arnold | 40°13'32.5"S | 73°21'35.7"W |
| ITS: PX230451 | IPB-CHL 32/22 | *Cortinarius mapuveronicae* | 08.05.2022 | Chile, Caramavida, Arauco Province | N. Arnold | 37°41'42.4"S | 73°07'52.7"W |
| ITS: PX230452 | IPB-CHL 43/22 | *Cortinarius mapuveronicae* | 18.05.2022 | Chile, Canete, Bío-Bío, Cañete | N. Arnold | 37°48'31.3"S | 73°09'38.2"W |
| ITS: PX230453 | IPB-CHL 51/22 | *Cortinarius mapuveronicae* | 21.05.2022 | Chile, Trongol Alto, Arauco Province | N. Arnold | 37°33'19.1"S | 73°11'00.1"W |
| ITS: PX230454 | IPB-CHL 92/22 | *Cortinarius mapuveronicae* | 28.05.2022 | Chile, Canete, Bío-Bío, Cañete | N. Arnold | 37°49'03.9"S | 73°05'21.5"W |
| ITS: PX230455 | IPB-CHL 19/23 | *Cortinarius mapuveronicae* | 13.05.2023 | Chile, La Union, Ranco Province | N. Arnold | 40°13'33.2"S | 73°21'35.4"W |
| ITS: PX230461 | IBF19630173 | ***Cortinarius rubrobasalis* TYPE** | 24.03.1963 | Argentina, Rio Negro | M. Moser |  |  |
| ITS: PX230456, LSU: PX230474 | IPB-CHL 64/22 | *Cortinarius rubrobasalis* | 21.05.2022 | Chile, Trongol Alto, Arauco Province | N. Arnold | 37°33'18.5"S | 73°10'59.8"W |
| ITS: PX230460, LSU: PX230477, RPB1: PX240690 | IBF20230155 | *Cortinarius teresae* | 13.04.2023 | Argentina, Chubut, Cushamen | L. Huymann, B. Siewert, U. Peintner | 42°5'57.15''S | 71°42'29.8''W |
| ITS: PX230459, LSU: PX230476 | WAT26645 | *Cortinarius veronicae* | 5.1995 | New Zealand, South Otago | R. Watling |  |  |

**Tab. S2** Key ions in the negative HRESIMS^2^ (^a^) and HRESIMS^3^ (^b^) spectra of the anthraquinones (**2-11**).

| Compound | *m/z* | Molecular formula | Key ions in the negative ESI-MS^2^ (^a^) and MS^3^ (^b^) spectra, *m/z* (elemental composition, rel. intensity [%]) | Structure |
| --- | --- | --- | --- | --- |
| (+)-7,7’-Emodinphyscion (2) | 551.0995 | C_31_H_19_O_10_^-^ | 520.0797 (C_30_H_16_O_9_^●-^, [M-H-OCH_3_]^●-^, 100.00)^a^  519.0721 (C_30_H_15_O_9_^-^, [M-H-OCH_3_]^-^, 100.00)^b^  503.0773 (C_30_H_15_O_8_^-^, [M-H-OCH_3_-OH]^-^, 21.03)^b^  492.0848 (C_29_H_16_O_8_^●-^, [M-H-OCH_3_-CO]^●-^, 12.85)^b^  474.0745 (C_29_H_14_O_7_^●-^, [M-H-OCH_3_-CO-H_2_O]^●-^, 10.39)^b^  448.0952 (C_27_H_12_O_7_^●-^, [M-H-OCH_3_-CO-H_2_O-C_2_H_2_]^●-^, 6.17)^b^ |  |
| Emodin (3) | 269.0458 | C_15_H_9_O_5_^-^ | 241.0511 (C_14_H_9_O_4_^-^, [M-H-CO]^-^, 23.04)^a^  225.0562 (C_14_H_9_O_3_^-^, [M-H-CO_2_]^-^,100.00)^a^  213.0561 (C_13_H_9_O_3_^-^, [M-H-2CO]^-^, 6.00)^b^  210.0325 (C_13_H_6_O_3_^●-^, [M-H-CO_2_-CH_3_]^●-^, 71.68)^b^  197.0610 (C_13_H_9_O_2_^-^, [M-H-CO_2_-CO]^-^, 30.26)^b^  181.0661 (C_13_H_9_O^-^, [M-H-2CO_2_]^-^, 100.00)^b^ |  |
| Emodin-6,8-di-*O*-methylether (4) | 297.1535 | C_17_H_13_O_5_^-^ | 283.0614 (C_16_H_11_O_5_^-^, [M-H-CH_3_]^-^, 9.32)^a^  268.0378 (C_15_H_8_O_5_^●-^, [M-H-2CH_3_]^●-^, 33.66)^a^  240.0429 (C_14_H_8_O_4_^●-^, [M-H-CH_3_-CO]^●-^, 100.00)^a^  183.0124 (C_12_H_7_O_2_^-^, [M-H-2CH_3_-OCH_3_-2CO]^-^, 5.20)^a^ |  |
| Questin (5) | 283.0613 | C_16_H_11_O_5_^-^ | 268.0379 (C_15_H_8_O_5_^●-^, [M-H-CH_3_]^●-^, 30.61)^a^  240.0430 (C_14_H_8_O_4_^●-^, [M-H-CH_3_-CO]^●-^, 100.00)^a^  212.0479 (C_13_H_8_O_3_^●-^, [M-H-CH_3_-CO-CO]^●-^, 100.00)^b^  196.0530 (C_13_H_8_O_2_^●-^, [M-H-CH_3_-CO-CO_2_]^●-^, 4.74)^b^ |  |
| (+)-(*S*)-Skyrin (6) | 537.0826 | C_30_H_17_O_10_^-^ | 519.0723 (C_30_H_15_O_9_^-^, [M-H-H_2_O]^-^, 21.46)^a^  493.0930 (C_29_H_17_O_8_^-^, [M-H-CO_2_]^-^, 100.00)^a^  469.0929 (C_27_H_17_O_8_^-^, [M-H-C_3_O_2_]^-^, 73.98)^a^  475.0823 (C_29_H_15_O_7_^-^, [M-H-CO_2_-H_2_O]^-^, 65.09)^b^  465.0975 (C_28_H_17_O_7_^-^, [M-H-CO_2_-CO]^-^, 92.00)^b^  449.1028 (C_28_H_17_O_6_^-^, [M-H-2CO_2_]^-^, 100.00)^b^  447.0876 (C_27_H_11_O_7_^-^, [M-H-CO_2_-H_2_O-2CH_3_]^-^, 26.68)^b^  437.1029(C_28_H_17_O_6_^-^, [M-H-2CO_2_]^-^, 3.28)^b^  434.0795 (C_27_H_14_O_6_^●-^, [M-H-2CO_2_-CH_3_]^●-^, 2.97)^b^  431.0927 (C_28_H_15_O_5_^-^, [M-H-2CO_2_-H_2_O]^-^, 4.03)^b^  421.1084 (C_27_H_17_O_5_^-^, [M-H-2CO_2_-CO]^-^, 8.08)^b^  405.1132 (C_27_H_17_O_4_^-^, [M-H-3CO_2_]^-^, 19.75)^b^ |  |

| (+)-(*S*)-Aurantioskyrin (7) | 553.0774 | C_30_H_17_O_11_^-^ | 535.0670 (C_30_H_15_O_10_^-^, [M-H-H_2_O]^-^, 100.00)^a^  517.0561 (C_30_H_13_O_9_^-^, [M-H-2H_2_O]^-^, 1.49)^a^  509.0880 (C_29_H_17_O_9_^-^, [M-H-CO_2_]^-^, 22.29)^a^  507.0717 (C_29_H_15_O_9_^-^, [M-H-H_2_O-CO]^-^, 100.00)^b^  491.0775 (C_29_H_15_O_8_^-^, [M-H-H_2_O-CO_2_]^-^, 2.93)^b^ |  |
| --- | --- | --- | --- | --- |
| Hypericin (8) | 503.0768 | C_30_H_15_O_8_^-^ | 487.0466 (C_29_H_11_O_8_^-^, [M-H-CH_4_]^-^, 19.49)^a^  475.0827 (C_29_H_15_O_7_^-^, [M-H-CO]^-^, 3.04)^a^  461.0672 (C_28_H_13_O_7_^-^, [M-H-CH_2_CO]^-^, 35.21)^a^  459.0877 (C_29_H_15_O_6_^-^, [M-H-CO_2_]^-^, 100.00)^a^  447.0880 (C_28_H_11_O_6_^-^, [M-H-2CO]^-^, 4.86)^a^  444.0643 (C_28_H_12_O_6_^●-^, [M-H-CO_2_-CH_3_]^●-^, 22.35)^b^  431.0928 (C_28_H_15_O_5_^-^, [M-H-CO_2_-CO]^-^, 100.00)^b^ |  |
| Dermolutein (9) | 327.0509 | C_17_H_11_O_7_^-^ | 283.0617 (C_16_H_11_O_5_^-^, [M-H-CO_2_]^-^, 100.00)^a^  268.0379 (C_15_H_8_O_5_^●-^, [M-H-CO_2_-CH_3_]^●-^, 24.85)^b^  265.0509 (C_16_H_9_O_4_^-^, [M-H-CO_2_-H_2_O]^-^, 44.10)^b^  240.0430 (C_14_H_8_O_4_^●-^, [M-H-2CO_2_-CH_3_-CO]^●-^, 100.00)^b^ |  |
| Endocrocin (10) | 313.0358 | C_16_H_9_O_7_^-^ | 269.0458 (C_15_H_9_O_5_^-^, [M-H-CO_2_]^-^, 100.00)^a^  241.0510 (C_14_H_9_O_4_^-^, [M-H-CO_2_-CO]^-^, 18.77)^b^  225.0560 (C_14_H_9_O_3_^-^, [M-H-2CO_2_]^-^,100.00)^b^  210.0324 (C_13_H_6_O_3_^●-^, [M-H-2CO_2_-CH_3_]^●-^, 0.19)^b^  197.0611 (C_13_H_9_O_2_^-^, [M-H-CO_2_-CO-CO_2_]^-^, 0.42)^b^  181.0661 (C_13_H_9_O^-^, [M-H-3CO_2_]^-^, 0.27)^b^ |  |
| Clavorubin (11) | 329.0291 | C_16_H_9_O_8_^-^ | 311.0193 (C_16_H_7_O_7_^-^, [M-H-H_2_O]^-^, 1.15)^a^  285.0398 (C_15_H_9_O_6_^-^, [M-H-CO_2_]^-^, 100.00)^a^  269.0455 (C_14_H_5_O_6_^-^, [M-H- H_2_O-CH_3_-CO]^-^, 8.12)^a^  270.0530 (C_14_H_5_O_6_^●-^, [M-H- H_2_O-CH_3_-CO]^●-^, 78.18)^b^  257.0451 (C_14_H_9_O_5_^-^, [M-H-CO_2_-CO]^-^, 100.00)^b^  241.0503 (C_14_H_9_O_4_^-^, [M-H-CO_2_-CO_2_]^-^, 95.34)^b^  226.0632 (C_13_H_6_O_4_^●-^, [M-H-CO_2_-CO_2_-CH_3_]^●-^, 2.63)^b^  217.0504 (C_9_H_9_O_3_^-^, [M-H-CO_2_-CO-C_3_O_2_]^-^, 15.63)^b^  213.0554 (C_13_H_9_O_3_^-^, [M-H-CO_2_-CO_2_-CO]^-^, 8.66)^b^ |  |

**Tab. S3** Collections of *C. mapuveronicae* used for pigment-chemical studies.

| IPB-CHL 9/18 | *Cortinarius mapuveronicae* | 03.05.2018 | Chile, La Union, Ranco Province | N. Arnold | 40°13'32.1"S | 73°21'36.9"W |
| --- | --- | --- | --- | --- | --- | --- |
| IPB-CHL 46/18 | *Cortinarius mapuveronicae* | 01.05.2018 | Chile, Nahuelbuta, Arauco Province, Parque Nacional Nahuelbuta | N. Arnold | 37°49'38.1"S | 73°00'36.6"W |
| IPB-CHL 61/18 | *Cortinarius mapuveronicae* | 26.05.2018 | Chile, Nahuelbuta, Arauco Province, Parque Nacional Nahuelbuta | N. Arnold | 37°49'39.1"S | 73°00'35.6"W |
| IPB-CHL 11/22 | *Cortinarius mapuveronicae* | 30.04.2022 | Chile, La Union, Ranco Province | N. Arnold | 40°13'32.5"S | 73°21'35.7"W |
| IPB-CHL 32/22 | *Cortinarius mapuveronicae* | 08.05.2022 | Chile, Caramavida, Arauco Province | N. Arnold | 37°41'42.4"S | 73°07'52.7"W |
| IPB-CHL 43/22, | *Cortinarius mapuveronicae* | 18.05.2022 | Chile, Canete, Bío-Bío, Cañete | N. Arnold | 37°48'31.3"S | 73°09'38.2"W |
| IPB-CHL 51/22 | *Cortinarius mapuveronicae* | 21.05.2022 | Chile, Trongol Alto, Arauco Province | N. Arnold | 37°33'19.1"S | 73°11'00.1"W |
| IPB-CHL 92/22 | *Cortinarius mapuveronicae* | 28.05.2022 | Chile, Canete, Bío-Bío, Cañete | N. Arnold | 37°49'03.9"S | 73°05'21.5"W |
| IPB-CHL 19/23 | *Cortinarius mapuveronicae* | 13.05.2023 | Chile, La Union, Ranco Province | N. Arnold | 40°13'33.2"S | 73°21'35.4"W |

**Tab. S4** Basidiospore sizes of measured *C. mapuveronicae* collections

| Collection | Basidiospore Size |
| --- | --- |
| IBF202200104 | (7.9) 8.8 ± 0.4 (9.9) x (5.0) 5.7 ± 0.3 (6.4) (n = 71) |
| IBF20220019 | (8.7) 9.3 ± 0.4 (10.6) x (5.5) 5.9 ± 0.3 (6.9) (n = 50) |
| IBF20220020 | (8.5) 9.5 ± 0.5 (11.1) x (5.4) 5.9 ± 0.2 (6.4) (n = 63) |
| IBF20220064 | (9.0) 9.7 ± 0.4 (10.8) x (5.6) 6.0 ± 0.2 (6.6) (n = 52) |
| IBF20220073B | (8.0) 8.7 ± 0.3 (9.3) x (5.4) 5.7 ± 0.2 (6.1) (n = 36) |
| IBF20230090 | (8.7) 9.4 ± 0.4 (10.5) x (5.3) 5.9 ± 0.3 (6.5) (n = 39) |
| IBF20230110 | (8.1) 9.8 ± 0.6 (11.0) x (4.4) 5.8 ± 0.3 (6.6) (n = 171) |

**Tab. S5** EC50 with positive and negative confidence interval (95%) of the isolated compounds **1**-**10** tested against A549 and T24 cells under dark (D) and blue light (BL) irradiation conditions. Colchicine was used as positive control.

|  | EC50 values [µg/mL] | | | | | | | |
| --- | --- | --- | --- | --- | --- | --- | --- | --- |
|  | T24 (D) | | T24 (BL) | | A549 (D) | | A549 (BL) | |
| Clavorubin-8-*O*-methylether (**1**) | >50 |  | >50 |  | >50 |  | 38.6 | 61.6 |
|  |  |  |  |  |  |  |  | 11.5 |
| (+)-7,7´-Emodinphyscion (**2**) | >50 |  | >50 |  | >50 |  | >50 |  |
| Emodin (**3**) | >50 |  | 20.8 | 79.6 | >50 |  | >50 |  |
|  |  |  |  | 12.2 |  |  |  |  |
| Emodin-6,8-di-*O*-methylether (**4**) | >50 |  | >50 |  | >50 |  | >50 |  |
| Questin (**5**) | >50 |  | >50 |  | >50 |  | >50 |  |
| (+)-(*S*)-Skyrin (**6**) | >50 |  | >50 |  | >50 |  | >50 |  |
| (+)-(*S*)-Aurantioskyrin (**7**) | >50 |  | 9.8 | 3.5 | >50 |  | >50 |  |
|  |  |  |  | 2.6 |  |  |  |  |
| Hypericin (**8**) | 2.0 | n.d | 0.2 | 0.9 | 9.2 | 5.9 | 0.4 | 0.4 |
|  |  | 2.0 |  | 0.2 |  | 3.6 |  | 0.2 |
| Dermolutein (**9**) | >50 |  | >50 |  | >50 |  | >50 |  |
| Endocrocin (**10**) | >50 |  | 9.8 | 3.5 | >50 |  | >50 |  |
|  |  |  |  | 2.6 |  |  |  |  |
| Colchicine | 0.1 | 0.5 | 0.1 | 0.5 | 0.2 | 0.3 | 0.2 | 0.4 |
|  |  | 0.1 |  | 0.1 |  | 0.1 |  | 0.1 |
